# Transcriptomic Analysis of Fumarate Compounds Identifies Unique Effects of Isosorbide Di-(Methyl Fumarate) on NRF2, NF-kappaB and IRF1 Pathway Genes

**DOI:** 10.3390/ph15040461

**Published:** 2022-04-11

**Authors:** William R. Swindell, Krzysztof Bojanowski, Ratan K. Chaudhuri

**Affiliations:** 1 Department of Internal Medicine, The Jewish Hospital, Cincinnati, OH 45236, USA; 2 Sunny BioDiscovery Inc., Santa Paula, CA 93060, USA; kbojanowski@sunnybiodiscovery.com; 3 Symbionyx Pharmaceuticals Inc., Boonton, NJ 07005, USA; ratan@sytheonltd.com; 4 Sytheon Ltd., Boonton, NJ 07005, USA

**Keywords:** astrocyte, dimethyl fumarate, diroximel fumarate, glial cells, Interferon regulatory factor, multiple sclerosis, neurodegeneration, neuroinflammation, NF-kappaB, NRF2

## Abstract

Dimethyl fumarate (DMF) has emerged as a first-line therapy for relapsing-remitting multiple sclerosis (RRMS). This treatment, however, has been limited by adverse effects, which has prompted development of novel derivatives with improved tolerability. We compared the effects of fumarates on gene expression in astrocytes. Our analysis included diroximel fumarate (DRF) and its metabolite monomethyl fumarate (MMF), along with a novel compound isosorbide di-(methyl fumarate) (IDMF). Treatment with IDMF resulted in the largest number of differentially expressed genes. The effects of DRF and MMF were consistent with NRF2 activation and NF-κB inhibition, respectively. IDMF responses, however, were concordant with both NRF2 activation and NF-κB inhibition, and we confirmed IDMF-mediated NF-κB inhibition using a reporter assay. IDMF also down-regulated *IRF1* expression and IDMF-decreased gene promoters were enriched with IRF1 recognition sequences. Genes altered by each fumarate overlapped significantly with those near loci from MS genetic association studies, but IDMF had the strongest overall effect on MS-associated genes. These results show that next-generation fumarates, such as DRF and IDMF, have effects differing from those of the MMF metabolite. Our findings support a model in which IDMF attenuates oxidative stress via NRF2 activation, with suppression of NF-κB and IRF1 contributing to mitigation of inflammation and pyroptosis.

## 1. Introduction

Multiple sclerosis (MS) is a chronic immune-mediated demyelinating disease affecting the central nervous system [1]. The estimated worldwide number of MS patients is 2.8 million and prevalence has increased in every region for which estimates are available [2]. MS is a significant source of mortality accounting for approximately 19,000 deaths worldwide in 2016 [3] with disease burden greatest at higher latitudes [4]. The pathophysiology is initiated by breakdown of basement membrane, endothelial cells, and tight junctions that comprise the blood–brain barrier (BBB) [5]. This leads to entry of T-cells into the CNS [6], which then undergo activation to release cytokines, such as TNF, IL-1, IL-6, IL-17, and IL-22 [7]. These cytokines have direct toxic effects on myelin surrounding axons [8], but additionally can enhance BBB degradation to promote an influx of B cells and macrophages [9]. Oligodendrocytes counter this neuroinflammatory cascade, by actively promoting remyelination, but over time disease progression occurs due to loss of oligodendrocytes and worsening inflammation [10]. This progression is seen on MRI by development of white matter plaques [11], along with symptoms, such as dysarthria, nystagmus, tremor, and autonomic dysfunction [12]. In the most common disease subtype, relapse-remitting multiple sclerosis (RRMS), alternating periods of demyelination, and remyelination occur with exacerbation and remission of symptoms [13]. There is no definitive MS cure, but steroids can treat acute exacerbations, and disease-modifying therapies, such as beta-interferon, natalizumab, glatiramer acetate, and ocrelizumab, may prolong remission [14].

Fumaric acid esters have an immunomodulatory mechanism of action with demonstrated efficacy for treatment of autoimmune diseases, such as psoriasis and multiple sclerosis [15]. The effectiveness of dimethyl fumarate (DMF) in the setting of multiple sclerosis (RRMS) was initially demonstrated by two phase III trials (DEFINE and CONFIRM) [16,17]. These trials demonstrated reduced rates of disease relapse among adults (ages 18–55) with recent disease activity. In both trials, flushing was the most reported adverse effect (24–38%), although gastrointestinal (GI) adverse effects occurred in 7 to 19% of patients. It has subsequently been reported that 36% of patients in these trials experienced adverse GI effects after 1 year [18], and in real-world settings, GI side effects led to DMF discontinuation in 5–19% of patients [19,20]. To address this issue, a novel agent diroximel fumarate (DRF) has been developed, which undergoes esterase cleavage in the GI tract to generate monomethyl fumarate (MMF), 2-hydroxyethyl succinimide (HES), RDC-8439, and methanol [21]. MMF is believed to be the active metabolite of DMF and DRF 462 mg and DMF 240 mg given twice-daily (BID) appear bioequivalent in terms of metabolic MMF generation. A 96-week study (EVOLVE-MS-1) showed that 30.9% of patients given DRF 462 mg BID still developed adverse GI effects, but such effects led to discontinuation in only 6.3% of patients [18]. Consistent with this, a 5-week study showed that the number of days with at least moderate GI symptoms is reduced by 46% in patients given DRF 462 mg BID as compared to DMF 240 mg BID [22].

The improved tolerability of DRF appears to be related to its unique chemical structure, which is mostly (>90%) metabolized to HES and MMF, with RDC-8439 and methanol being generated as minor products (<10%) [21]. The generation of high methanol concentrations in the small intestine is likely responsible for adverse GI effects seen with DMF and DRF treatment [23]. This has raised the possibility that other fumarate esters with alternative chemical structures may also, like DRF, demonstrate improved tolerability based upon differences in their metabolic byproducts. We recently developed a novel fumarate compound, isosorbide di-(methyl fumarate) (IDMF), consisting of two methyl fumarate groups attached to a central isosorbide moiety [24,25]. IDMF is predicted to be less reactive than DMF due to its chemical structure and higher activation free energy barrier [24]. When topically applied to skin, IDMF is less harsh compared to DMF, without evidence of irritation or sensitization in multiple animal models (i.e., rat, rabbit, guinea pig) [24]. A prior transcriptome study showed that treatment of astrocytes with IDMF decreased expression of genes associated with a proliferative reactive astrocyte phenotype as well as NF-κB target genes [25]. No prior study, however, has compared transcriptional effects of IDMF to those of MMF or DRF and, thus, it remains unclear whether these fumarate compounds differentially impact key disease pathways associated with MS progression.

This study used microarray profiling to evaluate the effects of three fumarate compounds on gene expression in cultured astrocytes (MMF, DRF, and IDMF). Astrocytes are critical for maintaining BBB integrity and interact with oligodendrocytes to influence remyelination and neuroinflammation [26]. We identify individual genes and co-expression modules regulated by each fumarate and evaluate their effects on genes linked to the nuclear factor-erythroid factor 2-related factor 2 (NRF2), nuclear factor kappa B (NF-κB), and hydroxycarboxylic acid receptor 2 (HCAR2/GPR109A) pathways [27]. Expression responses relevant to MS are established by comparisons to literature-based gene–disease association databases [28,29,30,31,32], loci previously associated with MS in genome-wide association studies (GWAS) [33], and expression profiles of astrocytes isolated from MS patient white matter using laser capture microdissection [34]. Our results uncover unique effects of IDMF not replicated by other fumarates and provide insights into cellular mechanisms by which such compounds may influence MS disease activity. 

## 2. Results

### 2.1. The MMF Expression Profile Differs from That of IDMF and DRF

**Figure 1:**
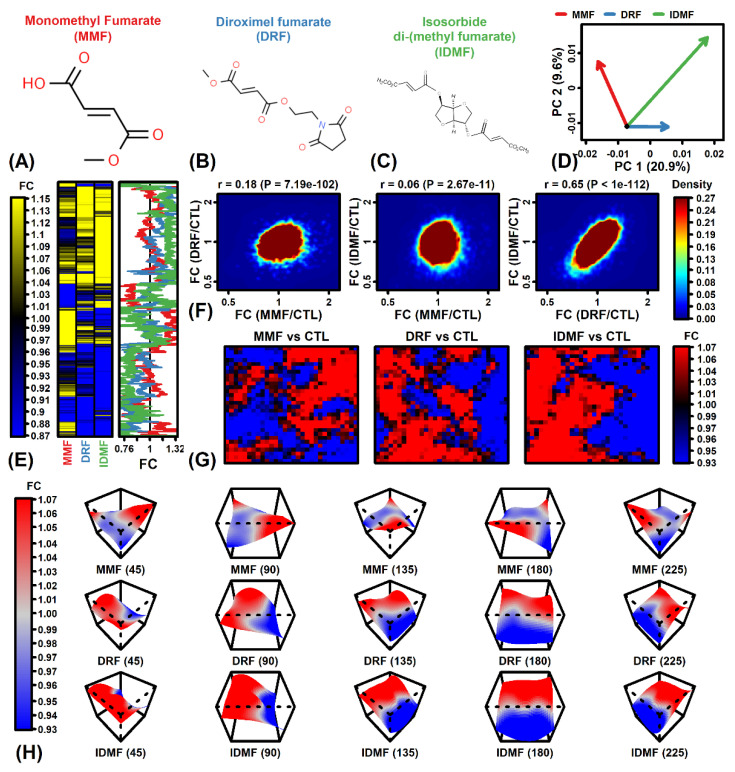
Differential expression comparison (MMF vs. DRF vs. IDMF). (**A**–**C**) Fumarate compound molecular structures. (**D**) PC differential expression vectors. Arrows start at the CTL treatment (bivariate mean) and terminate at the bivariate mean for each treatment (MMF, DRF, or IDMF). Longer arrows correspond to a stronger treatment effect. (**E**) Heatmap. FC change estimates are shown for the top-ranked 50% of genes (*n* = 6493) having the largest absolute FC estimate (|FC|) among the three differential expression comparison. (**F**) Scatterplots. FC estimates are plotted to compare effects of MMF, DRF, and IDMF. The density of genes in each region is indicated (see color scale) and the Spearman correlation coefficient and *p*-value are shown (top margin). (**G**) Self-organizing maps (SOMs). Genes were assigned to regions within an SOM and the average fold-change of genes within each region is shown (see color scale). (**H**) SOM surface plots. The three-dimensional surface indicates the average FC among genes assigned to each SOM region. Plots are shown with varying rotations for viewing at multiple angles (45, 90, 135, 180, and 225 degrees).

We treated human fetal astrocytes with sterile distilled water (CTL), MMF, DRF, or IDMF (2.5 µM each) for 24 h and evaluated gene expression using in situ oligonucleotide microarrays (see Methods). The three fumarate compounds (Figure 1A–C) had disparate effects on gene expression. This could be discerned from evaluation of differential expression vectors in a two-dimensional principal component space, which showed that DRF and IDMF shifted expression in a direction opposite from that of MMF (Figure 1D). Consistent with this, we identified groups of genes for which the estimated FC (MMF/CTL) differed from those calculated for DRF and IDMF (Figure 1E). Among all astrocyte-expressed genes, DRF/CTL and IDMF/CTL FC estimates were highly correlated with each other (r_s_ = 0.65), but weakly correlated with MMF/CTL FC estimates (r_s_ ≤ 0.18; Figure 1F). These trends were further supported by inspection of self-organizing maps color-coded based upon FC estimates, which revealed similar patterns for DRF and IDMF differing from that of MMF. 

### 2.2. IDMF Leads to Extensive Down-Regulation of Genes Associated with Metabolism and Post-Transcriptional Regulation

**Figure 2:**
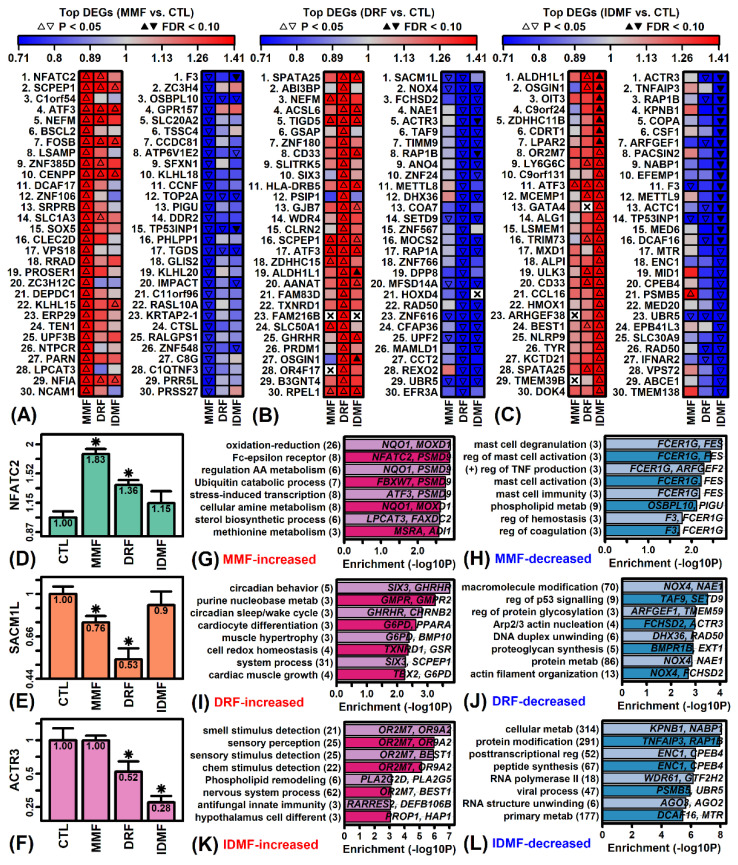
Top-ranked DEGs. (**A**–**C**) Top 30 DEGs most strongly altered by (**A**) MMF, (**B**) DRF, and (**C**) IDMF. The top-ranked 30 increased and decreased genes are shown for each compound (i.e., lowest *p*-values with FC > 1.25 or FC < 0.80). (**D**) *NFATC2*. (**E**) *SACM1L*. (**F**) *ACTR3*. In (**D**–**F**), average expression (log_2_ scale) is shown for each treatment (±1 standard error; * *p* < 0.05, comparison to CTL treatment). Expression is normalized to the average value in the CTL treatment for each gene. (**G**–**L**) GO BP terms enriched among (**G**,**I**,**K**) increased and (**H**,**J**,**L**) decreased DEGs. The number of DEGs associated with each GO term is given in parentheses (left margin) and example DEGs are listed within each figure.

Differentially expressed genes (DEGs) were identified using generalized least square linear models with empirical Bayes moderated t-statistics (see Methods) [35]. We identified 450, 379, and 1576 DEGs with respect to the MMF vs. CTL, DRF vs. CTL and IDMF vs. CTL comparisons, respectively (*p* < 0.05 with FC > 1.25 or FC < 0.80; Figure S1K–P; Tables S1–S6, Supplementary Materials). Most of the 30 genes most strongly increased or decreased by MMF were not correspondingly regulated by DRF and IDMF (Figure 2A). The strongest overall effect of MMF (i.e., lowest *p*-value) was up-regulation of nuclear factor of activated T cells 2 (NFATC2) (Figure 2D), although DEGs uniquely up-regulated by MMF included C1orf54, BSCL2, and LSAMP, and DEGs uniquely down-regulated by MMF included ZC3H4, GPR157 and SLC20A2 (Figure 2A). The complete set of MMF-increased DEGs was most strongly associated with oxidation–reduction, FC-epsilon receptor and amino acid metabolism (Figure 2G), whereas MMF-decreased DEGs were associated with mast cell degranulation/activation, positive regulation of TNF production, and phospholipid metabolism (Figure 2H). 

Most DRF-increased DEGs were not correspondingly altered by MMF or IDMF, although in contrast DRF-decreased genes were often correspondingly decreased by IDMF (Figure 2B). Examples of genes uniquely up-regulated by DRF include ABI3BP, GSAP, and ZNF180, while genes uniquely down-regulated by DRF include METTL8, COA7, and ZNF567 (Figure 2B). Overall, down-regulation of SAC1 like phosphatidylinositide phosphatase (SACM1L) was the strongest transcriptional effect of DRF (Figure 2E). The set of DRF-increased DEGs was associated with circadian behavior, purine nucleobase metabolism, and cardiocyte differentiation (Figure 2I), while DRF-decreased DEGs were associated with macromolecule modification, p53 regulation, and regulation of glycosylation (Figure 2J). 

The majority of genes strongly regulated by IDMF were not significantly altered by MMF or DRF (Figure 2C). Unique effects of IDMF include up-regulation of OIT3, C9orf24, and ZDHHC11B, and down-regulation of TNFAIP3, KPNB1, and COPA (Figure 2C). The strongest overall effect of IDMF was down-regulation of actin related protein 3 (ACTR3) (Figure 2F). The complete set of IDMF-increased DEGs was most strongly associated with olfactory detection, phospholipid remodeling, and antifungal innate immunity (Figure 2K). The set of IDMF-decreased DEGs included approximately 300 genes associated with cellular metabolism and protein modification, along with 50 genes associated with post-transcriptional gene regulation (Figure 2L).

Enrichment of motifs in 5 kb sequences upstream of DEGs was assessed using semiparametric generalized additive logistic models (Figure S2, Supplementary Materials) [36]. IDMF-increased DEG promoters were enriched with 5-CATG/CATG-3 and 5-ATGGG/CCCAT-3 elements (Figure S2D). MMF-increased DEGs and DRF/IDMF-decreased DEGs were enriched for AT-rich motifs (Figure S2B,C,G). Both DRF- and IDMF-decreased DEG promoters were significantly enriched for a 5-TAATT/AATTA-3 element recognized by paired related homeobox 1 and 2 (PRRX1 and PRRX2) (Figure S2G).

### 2.3. IDMF Up-Regulates Co-Expressed Tight Junction Genes and Represses Groups of Co-Expressed Genes Localized to Mitochondria

**Figure 3:**
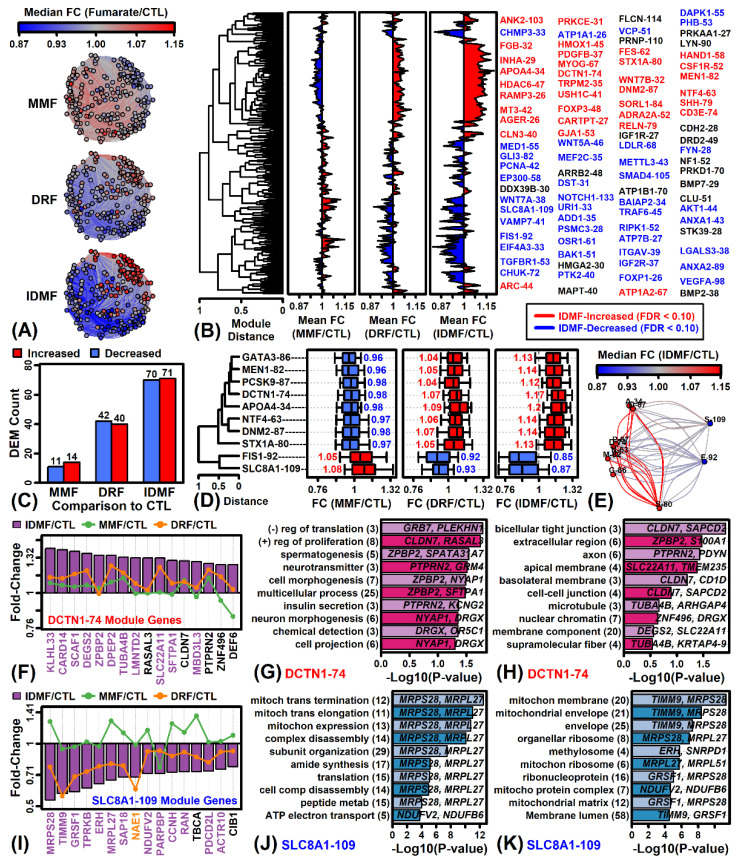
Gene expression module analysis. (**A**) Module co-expression networks. Each vertex represents one of 234 modules. Connections are drawn between modules for which medoids are correlated (*r*_s_ ≥ 0.80). Vertex color reflects the average FC of module genes. Edge color reflects the average FC of the two modules joined. (**B**) Module cluster analysis. The 234 module medoids were clustered based upon the Euclidean distance (normalized to the [0, 1] interval). Average FC for each module is shown and module IDs are listed (right margin). (**C**) Number of differentially expressed modules (DEMs) for each comparison (MMF vs. CTL, DRF cv. CTL, IDMF vs. CTL). (**D**) IDMF-responsive modules (cluster analysis). The 10 modules most strongly altered by IDMF are shown. Cluster analysis was performed using Euclidean distance (normalized to the [0, 1] interval). Boxplots outline the middle 50% of FC estimates for each module (whiskers: 10th to 90th percentiles). (**E**) IDMF-responsive modules (network). The 10 modules from (**D**) are shown (vertex labels are abbreviated). Network parameters and color-coding are as described for part (**A**) above. (**F**) Module DCTN1-74 genes. The 16 genes most strongly increased by IDMF are shown (i.e., lowest *p*-values). (**G**) GO BP terms enriched among module DCTN1-74 genes. (**H**) GO CC terms enriched among module DCTN1-74 genes. (**I**) Module SLC8A1-109 genes. The 16 genes most strongly decreased by IDMF are shown (i.e., lowest *p*-values). (**J**) GO BP terms enriched among module SLC8A1-109 genes. (**K**) GO CC terms enriched among module SLC8A1-109 genes. In (**G**,**H**,**J**,**K**), the number of genes associated with each term is listed in parentheses (left margin) and example genes are listed within the figure.

We next identified 234 gene expression modules (25–196 genes/module) by clustering genes based upon their expression across an independent set of astrocyte microarray samples (see Methods). Genes within each module had a stronger tendency to be altered in the same direction by IDMF as compared to DRF or MMF (Figure 3A,B). Consequently, we identified 141 modules differentially expressed by IDMF, whereas only 82 and 25 were identified by DRF and MMF, respectively (Figure 3C). Modules most strongly altered by IDMF were similarly altered but to a lesser degree by DRF, although usually MMF had opposite effects (Figure 3B,D,E). The module DCTN1-74 was most strongly up-regulated by IDMF (*p* = 9.77 × 10^−19^; FDR = 2.29 × 10^−16^; Figure 3D,E). Genes belonging to this module included KLHL33, CARD14, and SCAF1 (Figure 3F), and such genes were often localized to tight junctions (Figure 3H) and associated with inhibition of translation and positive regulation of proliferation (Figure 3G). Likewise, module SLC8A1-109 was most strongly down-regulated by IDMF (*p* = 1.33 × 10^−14^; FDR = 1.55 × 10^−12^; Figure 3D,E). Genes belonging to this module included MRPS28, TIMM9, and GRSF1 (Figure 3I), and such genes were frequently localized to the mitochondrial membrane (Figure 3K) and associated with mitochondrial translation and gene expression (Figure 3J).

### 2.4. The Effects of DRF and IDMF on Gene Expression Are Consistent with NRF2 Pathway Activation

**Figure 4:**
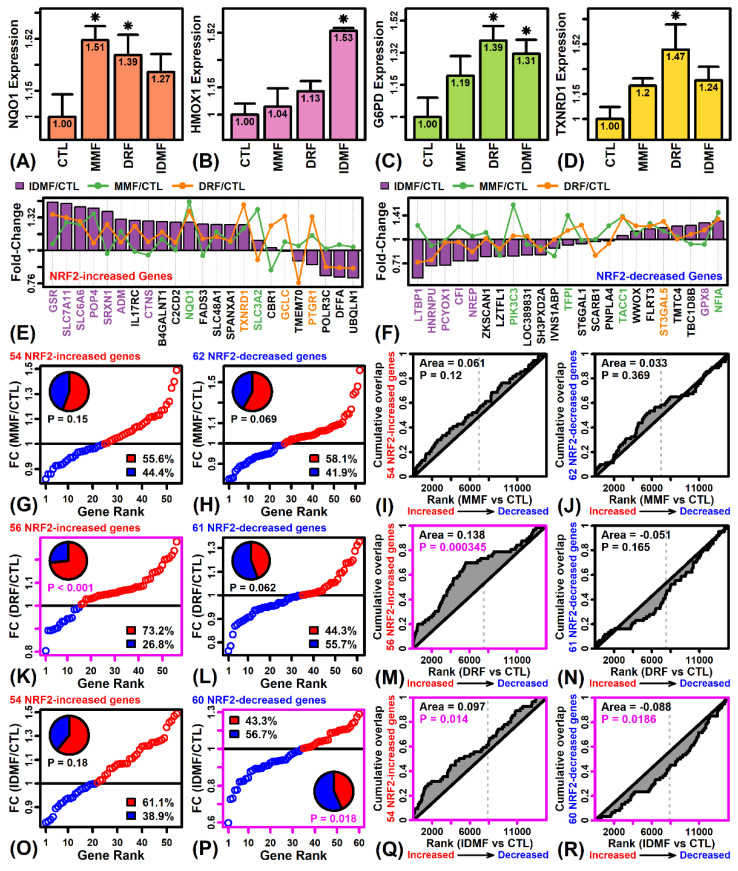
NRF2 target genes. (**A**) *NQO1*. (**B**) *HMOX1*. (**C**) *G6PD*. (**D**) *TXNRD1*. In (**A**–**D**), average expression (log_2_ scale) is shown for each treatment (± 1 standard error; * *p* < 0.05, comparison to CTL treatment). Expression is normalized to the average value in the CTL treatment for each gene. (**E**) NRF2-increased genes. (**F**) NRF2-decreased genes. In (**E**,**F**), the value of |FC|_max_ was calculated for each gene, where |FC|_max_ is defined as max [abs(FC_MMF_, FC_DRF_, FC_IDMF_)], and the 23 NRF2-increased or NRF2-decreased genes with highest value of |FC|_max_ are shown. Non-black gene labels are used if there is significant differential expression (*p* < 0.05) for any of the three comparisons, in which case the label color matches the comparison associated with the lowest differential expression *p*-value. (**G**,**H**,**K**,**L**,**O**,**P**) FC estimates for NRF2-responsive genes. FC estimates are plotted and the proportion of fumarate-increased (red) fumarate-decreased (blue) genes is shown (*p*-value: Fisher’s exact test). The percentage of increased/decreased genes is also indicated (see legend). (**I**,**J**,**M**,**N**,**Q**,**R**) The cumulate overlap is shown between NRF2-regulated genes and genes ranked based upon their response to each fumarate compound. A positive area statistic denotes enrichment among fumarate-increased genes, and a negative area statistic indicates enrichment among fumarate-decreased genes (*p*-value: Wilcoxon rank sum test).

MMF significantly increased expression of the NRF2 target gene NAD(P)H quinone dehydrogenase 1 (NQO1) (Figure 4A) but did not significantly increase expression of other NRF2 targets, such as HMOX1, G6PD, and TXNRD1 (Figure 4B–D). Each of these NRF2 targets, however, was significantly increased by DRF and/or IDMF (Figure 4A–D). To evaluate NRF2 regulation more systematically, we evaluated 143 genes recently identified as belonging to a NRF2 expression signature, including 68 genes up-regulated with NRF2 activation (Figure 4E) and 75 genes down-regulated with NRF2 activation (Figure 4F) [37]. There was no significant trend towards increased or decreased expression of signature genes with MMF treatment (Figure 4G–J). However, among 56 astrocyte-expressed genes up-regulated by NRF2 activation, 73% were correspondingly increased by DRF (*p* < 0.001; Figure 4K) and such genes were enriched among DRF-increased genes (*p* < 0.001; Figure 4M). On the other hand, there was no significant trend to indicate DRF-decreased expression of genes down-regulated with NRF2 activation (Figure 4L,N). Expression patterns associated with IDMF treatment were slightly more consistent with NRF2 activation. Most genes up-regulated by NRF2 activation were IDMF-increased (61%; *p* = 0.18; Figure 4O) and such genes were enriched among IDMF-increased genes (*p* = 0.014; Figure 4Q). Likewise, most genes down-regulated by NRF2 activation were IDMF-decreased (56%, *p* = 0.018; Figure 4P) and such genes were enriched among IDMF-decreased genes (*p* = 0.0186; Figure 4R).

### 2.5. The Effects of MMF and IDMF on Gene Expression Are Consistent with NF-κB Suppression

**Figure 5:**
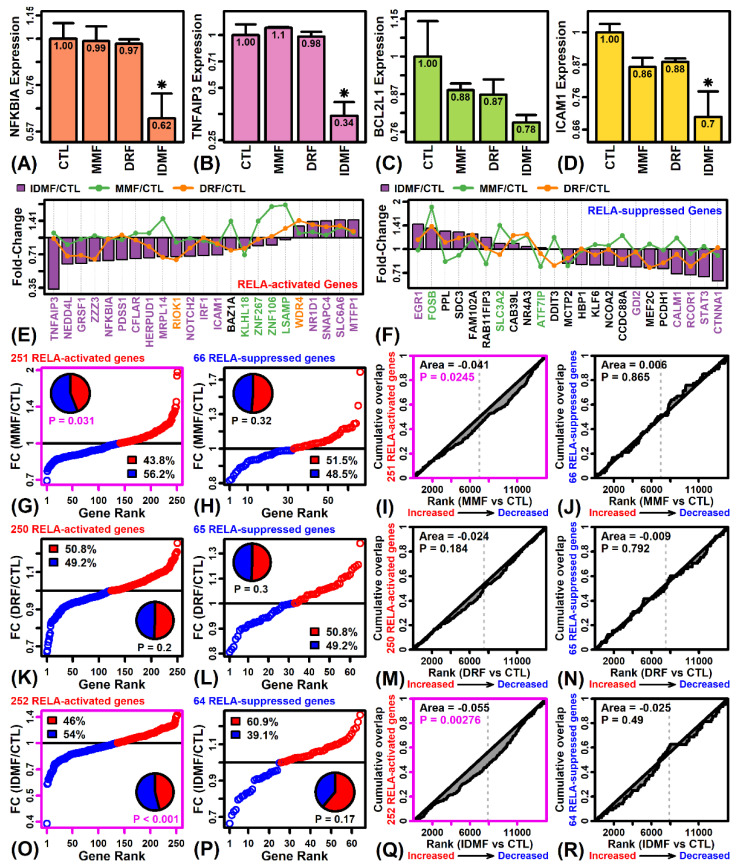
NF-kB (RELA) target genes. (**A**) *NFKBIA*. (**B**) *TNFAIP3*. (**C**) *BCL2L1*. (**D**) *ICAM1*. In (**A**–**D**), average expression (log_2_ scale) is shown for each treatment (±1 standard error; * *p* < 0.05, comparison to CTL treatment). Expression is normalized to the average value in the CTL treatment for each gene. (**E**) RELA-activated genes. (**F**) RELA-suppressed genes. In (**E**,**F**), the value of |FC|_max_ was calculated for each gene, where |FC|_max_ is defined as max [abs(FC_MMF_, FC_DRF_, FC_IDMF_)], and the 23 RELA-activated or RELA-suppressed genes with highest value of |FC|_max_ are shown. Non-black gene labels are used if there is significant differential expression (*p* < 0.05) for any of the three comparisons, in which case the label color matches the comparison associated with the lowest differential expression *p*-value. (**G**,**H**,**K**,**L**,**O**,**P**) FC estimates for RELA-activated genes. FC estimates are plotted and the proportion of fumarate-increased (red) fumarate-decreased (blue) genes is shown (*p*-value: Fisher’s exact test). The percentage of increased/decreased genes is also indicated (see legend). (**I**,**J**,**M**,**N**,**Q**,**R**) The cumulate overlap is shown between RELA-activated genes and genes ranked based upon their response to each fumarate compound. A positive area statistic denotes enrichment among fumarate-increased genes, and a negative area statistic indicates enrichment among fumarate-decreased genes (*p*-value: Wilcoxon rank sum test).

An initial analysis of four well-validated NF-κB targets (NFKBIA, TNFAIP3, BCL2L1, ICAM1) showed that each was more strongly down-regulated by IDMF compared to MMF and DRF (Figure 5A–D). We next focused on the expression of 389 NF-κB (RELA) targets recently identified from the genomic analysis of a B-cell lymphoma cell line (BJAB) [38]. This gene set included 304 RELA-activated direct targets induced by NF-κB-stimulating treatments, associated with RELA binding (ChIP-Seq), and down-regulated by the dominant negative NF-κB inhibitor alpha (dnIκBα) (Figure 5E) [38]. The gene set included additional 85 RELA-suppressed targets up-regulated by dnIκBα expression (Figure 5F) [38]. 

There was no significant evidence to support inhibition of NF-κB signaling by DRF (*p* ≥ 0.184; Figure 5K–N). However, most RELA-activated genes were down-regulated by MMF and IDMF (*p* ≤ 0.031; Figure 5G–O), and consistent with this such genes were enriched among genes decreased by MMF and IDMF (*p* ≤ 0.024; Figure 5I–Q). There was no significant evidence to demonstrate up-regulation of RELA-suppressed genes by MMF or IDMF (Figure 5H,P,J,R), although the majority of such genes were up-regulated by IDMF (*p* = 0.17; Figure 5P). 

We did not observe significant changes in the expression of genes encoding NF-κB subunit proteins (i.e., NFKB1, NFKB2, REL, RELA, RELB; Figure S3D–I, Supplementary Materials). Of six different NF-κB binding site motifs examined (Figure S3A), there was one motif significantly enriched in sequences upstream of IDMF-increased genes (*p* < 0.05; Figure S3B), but such motifs were not enriched in sequences upstream of genes decreased by any fumarate compound (Figure S3C). 

### 2.6. MMF Significantly Up-Regulates the Expression of Genes Induced via the Niacin-HCAR2 Pathway

The response to DMF is also mediated by the niacin-activated HCAR2 (GPR109A) receptor [27]. We did not observe significant changes in the expression of HCAR2 (Figure S4A, Supplementary Materials) or suspected niacin-HCAR2 pathway targets (Figure S4B–D) [27,39,40]. We thus focused on the expression of genes with altered expression in monocytes treated with niacin (100 μM) for 6 h (GSE103381) [41]. MMF was the only fumarate for which niacin-increased genes were biased towards up-regulated expression, consistent with activation of the niacin-HCAR2 pathway (Figure S4G–R). Nonetheless, we identified some individual niacin-responsive genes significantly altered by each fumarate compound (Figure S4E–F). 

### 2.7. IDMF Down-Regulates IRF1 Expression and IRF1 Binding Sites Are Enriched in Sequences Upstream of IDMF-Decreased Genes

Interferon regulatory factor 1 (IRF1) expression was significantly down-regulated by IDMF (Figure S5D, Supplementary Materials) although genes encoding other IRF factors were not significantly altered by any fumarate (Figure S5E,F,H–J). We evaluated enrichment of 19 IRF family motifs (Figure S5A) within 5 kb upstream regions of genes significantly altered by each fumarate (Figure S5B,C). An IRF1 motif was significantly enriched (FDR < 0.05) in regions upstream of MMF-increased DEGs, and there was marginal enrichment (*p* < 0.05) of IRF4 motifs in regions upstream of genes increased by MMF or IDMF (Figure S5B). Promoters of IDMF-decreased genes were strongly enriched (FDR < 0.05) with motifs linked to IRF1, IRF4, IRF6, and IRF7, and likewise promoters of DRF-increased genes were enriched for motifs associated with IRF1 and IRF7 (Figure S5C). Predicted IRF1 targets among IDMF-decreased genes included MID1, PSMB5, and GTF2H3 (Figure S5G). Putative IRF1 binding sites identified near such genes were concentrated approximately 500 kb upstream from each gene’s transcription start site (Figure S5K).

### 2.8. IDMF Decreases the Expression of A1 Reactive Astrocyte Marker Genes Associated with Cell Death, Catabolism, and Lysosomes

Astrocytes develop a reactive phenotype in response to CNS injury or disease [42]. The A1 and A2 sub-phenotypes are two reactive astrocyte subtypes described previously, with the A1 phenotype viewed as neurotoxic and the A2 phenotype viewed as neuroprotective [43]. The expression of two A1 marker genes, complement C3 (C3) and guanylate binding protein 2 (GBP2), were not significantly altered by MMF, DRF, or IDMF (Figure S6A,B, Supplementary Materials). Likewise, expression of A2 markers S100 calcium binding protein A10 (S100A10) and transforming growth factor beta 1 (TGFB1) were not significantly altered by any fumarate (Figure S6C,D). 

We next evaluated the expression of gene sets associated with the A1 phenotype (*n* = 161 genes), A2 phenotype (*n* = 75), and pan-reactive astrogliosis (A1 + A2) [25,44]. MMF led to a significant (*p* = 0.03) but slight (3%) increase in the average expression of A2 genes (Figure S6E) but only altered expression of some A1 and no A2 marker genes (Figure S6F,G). DRF altered the expression of some A1 and A2 marker genes (Figure S6I,J) but there was no significant systemic pattern among all marker genes (Figure S6H). IDMF decreased expression of A2 marker genes by only 1% on average although the median FC differed significantly from other astrocyte-expressed genes (*p* = 0.04; Figure S6K). Notably, we identified 19 A1 and 6 A2 marker genes significantly increased or decreased by IDMF (*p* < 0.05; Figure S6L,M). Of the A1 marker genes, 11 were IDMF-decreased and these were associated with catalytic activity, positive regulation of programmed cell death, and lysosomes (Figure S6N–P).

### 2.9. IDMF Alters the Expression of More MS-Associated Genes Compared to MMF and DRF

**Figure 6:**
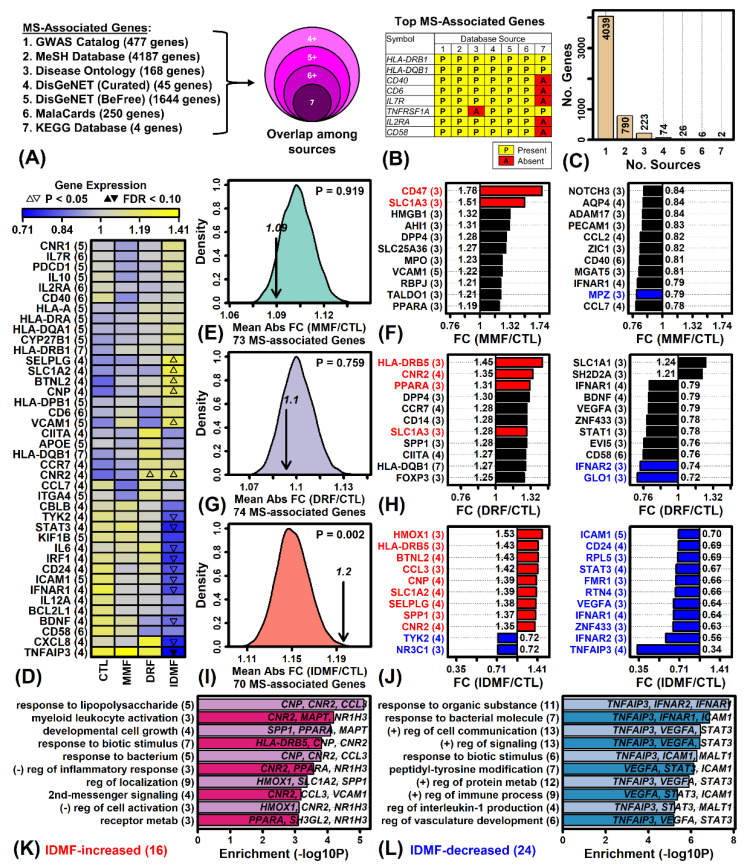
MS-associated genes. (**A**) MS gene databases. (**B**) Top MS-associated genes (six or more database sources). (**C**) Number of sources for MS-associated genes. (**D**) MS-associated genes (four or more sources) and their response to MMF, DRF, and IDMF. The 40 genes most strongly altered by one of the three compounds are shown (i.e., lowest *p*-value). (**E**,**G**,**I**) Average absolute FC (|log_2_(FC)|) of MS-associated genes (four or more sources). The null distribution shown was obtained by calculating the absolute FC in randomly sampled gene sets of the same size (10,000 simulation trials). (**F**,**H**,**J**) MS-associated genes most strongly altered by each compound (four or more sources). The number of database sources linking each to MS is shown in parentheses. Significantly altered genes (*p* < 0.05) are shown in red (increased) or blue (decreased) font. (**K**,**L**) GO BP terms enriched among MS-associated genes (three or more sources) altered by IDMF (*p* < 0.05 with FC > 1.25 or FC < 0.80). The number of genes associated with each GO BP term is indicated in parentheses (left margin) and example genes are listed within each figure.

MS-associated genes were identified from seven database sources (Figure 6A). Only two genes were linked to MS by all seven sources (HLA-DRB1 and HLA-DQB1) while six genes were linked to MS by six of the seven sources (Figure 6B). Only 6% of the MS-associated genes were linked to MS by three or more database sources (i.e., 331 of 5160 genes; Figure 6C). Several genes linked to MS by four or more sources were significantly altered by IDMF but not by MMF or DRF (e.g., TNFAIP3, CXCL8, VCAM1; Figure 6D). On average, MMF and DRF altered expression of MS-associated genes (4+ sources) by 9% and 10%, respectively, which was not significantly different compared to other genes with detectable expression (*p* ≥ 0.759; Figure 6E,G). However, IDMF altered the expression of MS-associated genes by 20% on average, which was significantly greater than that seen for other expressed genes (*p* = 0.002; Figure 6I). A larger number of MS-associated genes were also significantly altered by IDMF compared to MMF and DRF (*p* < 0.05; Figure 6F,H,I). For example, IDMF significantly increased expression of HMOX1, HLA-DRB5, and BTNL2, and decreased expression of TNFAIP3, IFNAR1, and ICAM1 (*p* < 0.05; Figure 6J). As a group, IDMF-increased genes (*p* < 0.05, FC > 1.25) associated with MS (3+ sources) were associated with response to LPS, myeloid leukocyte activation, and negative regulation of inflammatory response (Figure 6K). Likewise, IDMF-decreased genes (*p* < 0.05, FC < 0.80) associated with MS (3+ sources) were associated with bacterial response, positive regulation of immune process, and IL-1 production (Figure 6L).

### 2.10. Genes Most Strongly Altered by MMF, DRF, and IDMF Overlap Significantly with Genes near MS GWAS Loci

**Figure 7:**
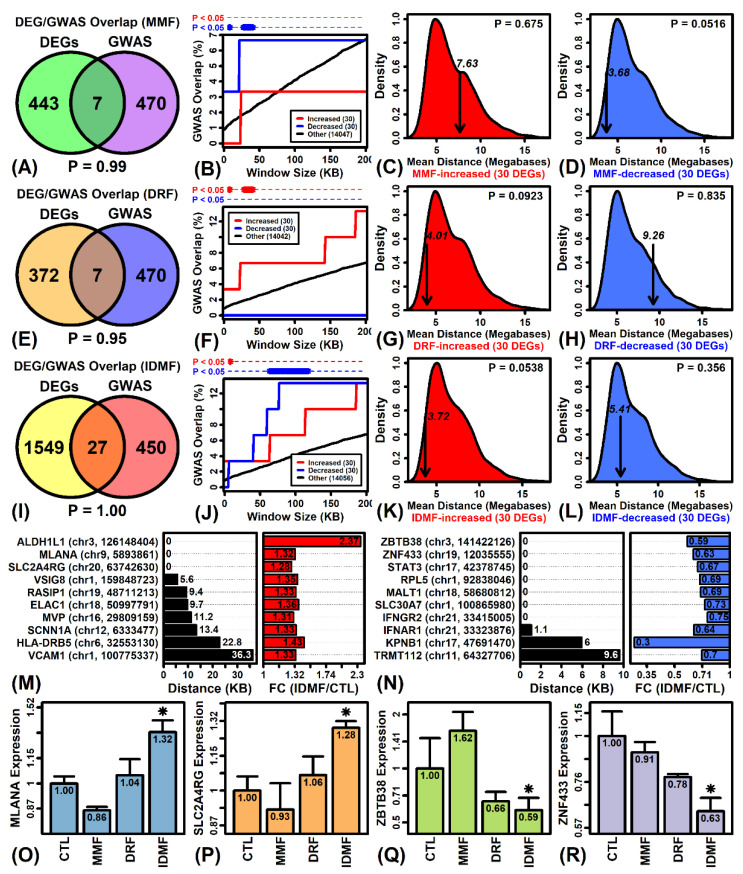
MS-associated genes from GWAS studies and their overlap with DEGs. (**A**,**E**,**I**) Overlap between DEGs and MS-associated genes from the NHGRI-EBI GWAS Catalog (reported, mapped, upstream, or downstream). DEGs were identified based on the same threshold in each analysis (*p* < 0.05, FC > 1.25 or FC < 0.80; bottom *p*-value: Fisher’s exact test for overlap). (**B**,**F**,**J**) Genes at varying distances from MS GWAS loci (horizontal axis) and their overlap with the top 30 genes most strongly increased/decreased by MMF, DRF, or IDMF (black line: overlap with respect to all other genes). Cases of significant overlap are indicated in the top margin (*p* < 0.05, Fisher’s exact test). (**C**,**D**,**G**,**H**,**K**,**L**) Average distance between the top 30 genes most strongly altered by MMF, DRF, or IDMF and the nearest MS GWAS locus (arrow). The distribution is obtained from 1000 simulation trials in which 30 genes were selected at random, with the average distance to the nearest MS GWAS locus calculated in each trial. (**M**,**N**) IDMF-increased/decreased genes nearest to an MS GWAS locus. For each gene, the distance to the nearest MS GWAS locus is shown (left) along with estimated fold-change (IDMF/CTL) (right). (**O**–**R**) Selected IDMF-responsive genes near MS GWAS loci. Average expression (±1 standard error) is shown for each treatment (* *p* < 0.05, comparison to CTL).

We next evaluated overlap between DEGs and those genes near MS-associated loci from the NHGRI-EBI GWAS catalog. Genes significantly altered by MMF, DRF, and IDMF (increased and decreased) did not overlap significantly with MS-associated genes from GWA studies, although such genes were approximately four times more frequent among genes altered by IDMF compared to MMF or DRF (Figure 7A,E,I). However, among the 30 genes most strongly altered by MMF, DRF, or IDMF (i.e., lowest *p*-value), there was significant overlap between such DEGs and genes located varying distances from MS GWAS loci (Figure 7B,F,J). For IDMF, the top 30 increased and decreased genes overlapped significantly with genes near GWAS loci (Figure 7J), whereas for other compounds overlap was only significant for decreased (MMF) or increased (DRF) genes (Figure 7B,F). As an alternative approach, we examined the average distance between the top 30 DEGs and MS GWAS loci (Figure 7C,D,G,H,K,L). This average distance was marginally less than expected (*p* < 0.10) in the case of MMF-decreased, DRF-increased, and IDMF-increased genes (Figure 7D,G,K). We further identified several IDMF-regulated genes that were overlapping with or near MS-associated GWAS loci (Figure 7M,N), including ALDH1L1, MLANA, SLC2A4RG, ZBTB38, and ZNF433 (Figure 7O–R).

### 2.11. IDMF Stimulates the Expression of Transcriptional Activation Pathways Correspondingly Up-Regulated in MS Patient Astrocytes

We next evaluated overlap between DEGs and genes with altered expression in astrocytes obtained from normal appearing white matter within brain tissue sections of MS patients (GSE83670) [34]. The effects of MMF on gene expression were negatively correlated with those seen in MS astrocytes (r = −0.03), with a significant overabundance (33.5%) of genes increased by MMF and decreased in MS astrocytes (Figure S7A, Supplementary Materials). Consistent with this, there was no significant overlap between genes altered by MMF and genes correspondingly altered in MS astrocytes (Figure S7B–D). Gene expression shifts in DRF- and IDMF-treated astrocytes were positively correlated with those seen in MS astrocytes (Figure S7E,I). There was significant overlap between DRF-decreased DEGs and MS-decreased genes (Figure S7G), but not with respect to DRF-increased genes or genes altered by IDMF (Figure S7F,J,K). GSEA analysis, however, showed that the top 100 genes increased by IDMF overlapped significantly with those genes increased in MS astrocytes, while the top 100 genes decreased by IDMF overlapped significantly with genes decreased in MS astrocytes (Figure S7L). This significant pattern was otherwise only seen with respect to DRF-decreased genes (not DRF-increased genes; Figure S7H). Genes increased by both IDMF and in MS astrocytes included MYO15B, TP53INP2, and RASSF2 (Figure S7M), and such genes were associated with positive regulation of transcription, developmental process, and cell differentiation (Figure S7O). Genes decreased by both IDMF and in MS astrocytes included SPPL2A, ABCE1, and PRKCI, and such genes were associated with macromolecule methylation, ephrin receptor signaling, and response to wounding (Figure S7P).

### 2.12. IDMF Represses NF-κB Induction in TNF-Stimulated Astrocytes

**Figure 8:**
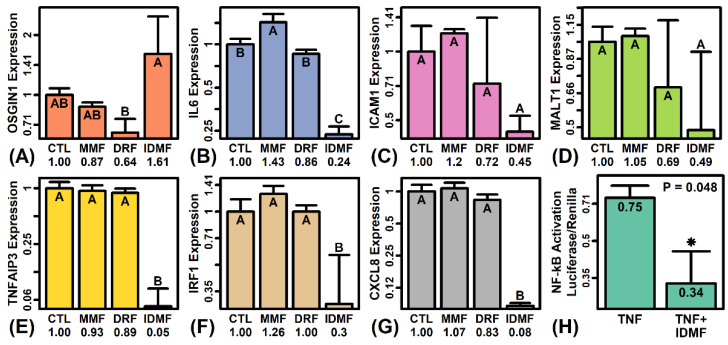
RT-PCR analyses and NF-κB luciferase reporter assay. (**A**–**G**) RT-PCR analyses (*OSGIN1*, *IL6*, *ICAM1*, *MALT1*, *TNFAIP3*, *IRF1,* and *CXCL8*). Average relative expression is shown for each gene (±1 standard error; *n* = 3 replicates per treatment). Relative expression was calculated using the 2^−∆∆Ct^ method and ∆Ct values were further normalized to the CTL treatment. Average relative expression is shown for each treatment (bottom margin). Treatments with different letters have significantly different average expression (*p* < 0.05, Fisher’s least significant difference). (**H**) NF-κB reporter assay. Human fetal astrocytes were transfected with firefly/Renilla luciferase constructs and treated with TNF-α (20 ng/ml) alone (*n* = 6) or TNF-α (20 ng/mL) + IDMF (2.5 µM) (*n* = 3) for 6 h. The ratio of luciferase and Renilla (internal control) signals was used to monitor NF-κB induction. The average ratio value is shown (±1 standard error) for each treatment (*p*-value: two-sample Wilcoxon rank sum test).

RT-PCR analyses were done to confirm microarray findings for selected IDMF-regulated genes (Figure 8B–H). IDMF increased expression of OSGIN1 by 60% based upon PCR, consistent with array findings (*p* < 0.05, Fisher’s LSD; Figure 8B). IDMF repressed expression of ICAM1 and MALT1, although this effect did not reach statistical significance (*p* > 0.05, Figure 8D,E). However, IDMF significantly decreased expression of IL6, TNFAIP3, IRF1 and CXCL8 (*p* < 0.05 each; Figure 8C,F,G,H). In the case of TNFAIP3 and CXCL8, expression in IDMF-treated cells was less than 10% of that in the CTL treatment.

Repression of TNFAIP3 (A20) and CXCL8 (IL8) expression by IDMF may be due to inhibition of the NF-κB pathway [45,46], which was suggested by our microarray analysis of NF-κB-responsive genes (Figure 5). To address this possibility, we transfected human fetal astrocytes with NF-κB reporter assay firefly/Renilla luciferase constructs and treated these cells with TNF-α alone (*n* = 6) or TNF-α + IDMF (*n* = 3) for 6 h. NF-κB induction was monitored using a dual-luciferase reporter assay system (i.e., luciferase/Renilla ratios) (Figure 8A). The NF-κB activation signal was reduced by approximately 50% in astrocytes treated with TNF + IDMF as compared to cells treated with TNF alone (*p* = 0.048, Wilcoxon rank sum test) (Figure 8A). IDMF thus repressed NF-κB induction in TNF-stimulated astrocytes.

### 2.13. IDMF Undergoes In Vitro Hydrolysis by Carboxylesterase to Generate MMF and IMMF

Carboxylesterase 2 (CES-2) is an endoplasmic reticulum serine esterase mediating first-pass hydrolysis of drugs in the small intestine [47]. We performed an in vitro study to assess fatty acid ester hydrolysis of IDMF by carboxylesterase-2. HPLC analyses showed that IDMF yielded MMF and isosorbide mono-(methyfumarate) (IMMF) as hydrolyzed products (Figure S8A, Supplementary Materials). IDMF was completely converted to MMF and IMMF after 15 min under assay conditions (Figure S8B–E). IDMF can therefore act as an MMF prodrug, similar to DRF, although it also generates a structurally similar compound (IMMF) as a secondary hydrolysate.

## 3. Discussion

**Figure 9:**
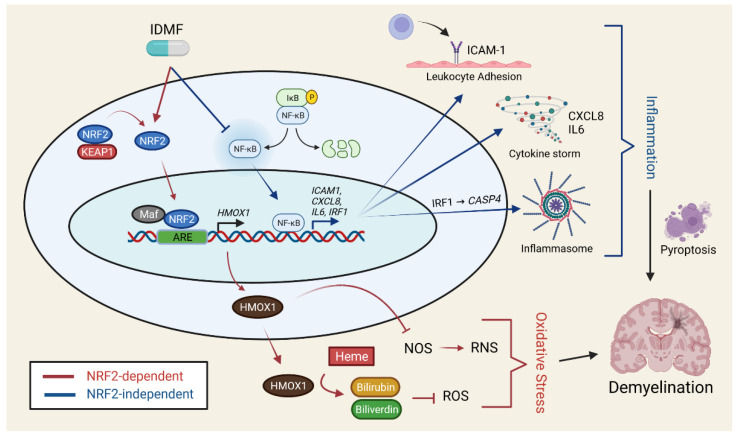
IDMF hypothesized mechanisms of therapeutic effect. IDMF activates NRF2-dependent signaling (red arrows) while inhibiting NF-κB activity and transcription of downstream genes (blue). IDMF triggers dissociation of NRF2 from KEAP1 to allow nuclear translocation of NRF2. Nuclear NRF2 dimerizes with musculoaponeurotic fibrosarcoma (Maf) and binds antioxidant response elements (ARE) to induce the transcription of target genes, such as *HMOX1*. The HMOX1 protein then acts as an enzyme to catalyze the formation of antioxidants such as bilirubin and biliverdin, while also inhibiting nitric oxide synthase (NOS). Together, these effects of HMOX1 reduce accumulation of reactive oxygen and reactive nitrogen species (ROS and RNS) to attenuate oxidative stress. The inhibition of NF-κB by IDMF prevents nuclear translocation and up-regulation of target genes such as *ICAM1*, *CXCL8*, *IL6*, and *IRF1*. Reduced expression of these targets leads to reduced inflammation via attenuation of ICAM1-dependent leukocyte adhesion, cytokine production with cytokine storm, and IRF1/*CASP4*-dependent inflammasome activation. Overall dampening of these inflammatory processes inhibits pyroptosis and, thus, limits progression of demyelination. See text for details. (Figure created with BioRender.com).

This study used a transcriptomics approach to evaluate direct effects of MMF, DRF, and a novel fumarate (IDMF) on gene expression in cultured astrocytes. The effects of DRF and IDMF differed from those of MMF, although we could identify unique responses to each fumarate, with IDMF having the largest overall effect in terms of the number of differentially expressed genes and modules identified (Table 1). Gene expression responses to IDMF were consistent with NRF2 activation as well as inhibition of the NF-κB and IRF1 pathways (Figure 9 and Table 1). Genes most strongly altered by each fumarate overlapped significantly with genes near loci implicated in MS by genetic association studies, although genes altered by IDMF overlapped more strongly with such genes (Figure 7), and our analysis identified several MS-associated genes uniquely altered by IDMF alone (e.g., down-regulation of *TNFAIP3*, *CXCL8*, *IL6*, and *IRF1*) (Figure 6). These results demonstrate ways in which fumarate compounds lack bioequivalence in terms of their direct effect on CNS glial cells. This may have implications regarding therapeutic efficacy and potential for adverse effects when different fumarate compounds are used in vivo for treatment of MS or other autoimmune diseases. 

The DMF mechanism of action has frequently been understood in terms of NRF2-dependent and NRF2-independent pathways, with the former postulated to mediate a neuroprotective effect on CNS cell types. NRF2 activation by DMF leads to transduction via the ERK1/2 MAPK pathway [48] with binding of NRF2 to antioxidant response elements (ARE) and induction of cytoprotective antioxidant genes having direct neuroprotective effects [49]. The importance of this mechanism was emphasized by early studies revealing that beneficial effects of DMF in a mouse model of chronic experimental autoimmune encephalitis (EAE) are abolished on the *Nrf2*(−/−) background [50]. DMF and MMF also lead to NRF2-dependent improvement in astrocyte viability following oxidative stress [49]. These effects of the NRF2 pathway are mediated by NRF2 target genes [51], but the current study showed that such targets respond differently to each fumarate compound. This was demonstrated using an externally validated NRF2 gene signature [37]. IDMF was the only fumarate to significantly increase expression of NRF2-increased genes and decrease expression of NRF2-decreased genes (Figure 4). IDMF also led to a potentially synergistic increase in *G6PD* and *GSR* expression (Figure 4). Glucose-6-phosphate dehydrogenase (G6PD) encodes a pentose phosphate pathway enzyme responsible for generation of NADPH [52], which is then required by glutathione disulfide reductase (GSR) to generate reduced glutathione [53]. Joint upregulation of both genes by IDMF may thus enhance GSR-dependent glutathione recycling, which is proposed to be a mechanism underlying the antioxidant activity of DMF [49,54,55]. IDMF also uniquely upregulated expression of the NRF2 target heme oxygenase-1 (*HMOX1*) (also HO-1) (Figure 4B). This gene encodes another NADPH-dependent enzyme, which catalyzes heme degradation to generate the antioxidants biliverdin and bilirubin [56] and further inhibits nitric oxide synthase to limit reactive nitrogen species production [51] (Figure 9). Fibroblasts lacking *HMOX1* thus demonstrate hypersensitivity to oxidative stress agents [57] and induction of *HMOX1* in astrocytes appears to mediate antioxidant effects of compounds such as resveratrol [58]. Fumarates we tested may thus have differing effects on redox homeostasis via such mechanisms.

The NRF2-independent effects of fumarates comprise a second set of mechanisms associated with inhibition of neuroinflammation with immunomodulatory effects on peripheral immune cells. These mechanisms are highlighted by the ability of DMF to ameliorate symptoms in an *acute* EAE mouse model on either a wild-type or *Nrf2*(−/−) genetic background [59]. Inhibition of NF-κB is an effect of DMF that is at least partly NRF2-independent, and we showed that an independently defined and validated group of NF-κB-activated genes [38] is down-regulated by MMF and IDMF but not by DRF (Figure 5). Several of such NF-κB target genes were down-regulated only by IDMF, such as *TNFAIP3*, *NFKBIA*, and *ICAM1* (Figure 5A,B,D). Down-regulation of NF-κB targets by IDMF is consistent with findings from our earlier study also performed in astrocytes [25]. Both *TNFAIP3* and *NFKBIA* encode inhibitory proteins that suppress the NF-κB pathway, and their down-regulation by IDMF is likely a compensatory response to loss of NF-κB signaling. *TNFAIP3* (A20) encodes a deubiquitinating enzyme previously linked to MS by genetic association studies [60]. TNFAIP3 protein abundance is increased in white matter and cortical lesion astrocytes from MS patient brains [61], although mRNA expression is decreased in peripheral monocytes from MS patients [62]. Similarly, *NFKBIA* (IκBα) encodes an NF-κB inhibitor for which promoter polymorphisms have been associated with MS [63,64], and abundance of this protein is prominent in macrophage nuclei of MS plaques undergoing active demyelination [65]. The unique down-regulation of *ICAM1* (CD54) by IDMF in this study may be a direct anti-inflammatory effect not seen with other fumarates [66] (Figure 9 and Table 1). *ICAM1* encodes an endothelial adhesion molecule that promotes leucocyte extravasation, and its expression in astrocytes is regulated by NF-κB downstream of stimulation by cytokines such as TNF-α and IL-1β [67,68]. These findings support NF-κB inhibition as a NRF2-independent mechanism by which IDMF may have an anti-inflammatory effect (Figure 9).

Pro-inflammatory cytokines are downstream mediators within the NF-κB signaling pathway inhibited by DMF in a NRF2-independent fashion [69]. Consistent with this, a unique effect of IDMF in this study was down-regulation of the gene encoding IL-6 (Figure 9 and Table 1), which has now emerged as a therapeutic target in multiple autoimmune diseases [70]. In MS patients, IL-6 within the central nervous system is localized to astrocytes and concentrated in demyelinated regions [71,72]. This localized expression of IL-6 in the CNS may be synergistically enhanced by elevated IL-6 production in circulating blood cells [73,74], which can be inhibited by DMF in some cell types (e.g., B cells) [75]. Consistent with these results, a neutralizing IL-6 antibody delayed disease progression in EAE mice by inhibiting microglia activation, CNS inflammation and myelin loss [76], and astrocyte-specific knockout of IL-6 in such mice reduced inflammatory cell infiltration and demyelination [77]. Toxic effects of astrocyte-generated IL-6 have also been supported by mouse models with transgenic over-expression of IL-6 in astrocytes, which exhibit a neurologically diseased phenotype characterized by tremors, ataxia, and seizures, with loss of neuronal subpopulations and increased reactive astrocytosis and neovascularization [78]. These pathological effects of local IL-6 persist in the absence of IL-6 production by circulating blood cells [79]. In this study, neither MMF nor DRF inhibited *IL6* mRNA levels in cultured astrocytes (Figure 6). In prior work, MMF was shown to increase *IL6* expression in spinal cord from mice following ventral root crush lesion [80], although MMF was also reported to decrease *IL6* expression in hippocampus [81], B cells [75], cardiomyocytes [82], liver [83], and intervertebral disk nucleus pulposus cells [84]. Effects of MMF and other fumarates on IL-6 production may therefore vary across contexts and cell types, although our work supports *IL6* mRNA down-regulation as a unique effect of IDMF in astrocytes.

Genetic IRF1 variants have been associated with MS [85] and some treatments such as glatiramer acetate block cytokine-stimulated IRF1 up-regulation [86]. Multiple studies have reported resistance of IRF1(−/−) mice to EAE, although the precise cell type mediating this effect in unclear. Prior work has suggested that EAE progression in mice is mediated by the activity of IRF1 in spinal cord [87], myelin-specific T cells [88], and glial cells within the CNS, such as oligodendrocytes [89,90,91]. In this study, IDMF down-regulated IRF1 expression and multiple motifs recognized by IRF1 were enriched in upstream regions of genes down-regulated by DRF and IDMF (Figure S5). Down-regulation of IRF1 may attenuate cytotoxicity in oligodendroglial progenitor cells resulting from IFN-gamma pathway signaling [92,93]. Repression of the IRF1 pathway may also limit pyroptosis in oligodendrocytes by preventing pro-inflammatory caspase protein activation [94]. Along these lines, *CASP4* expression was significantly down-regulated by IDMF but not by MMF or DRF. Finally, IRF1 repression is expected to down-regulate BAFF expression, which may attenuate non-canonical NF-κB activation [95] and induce apoptosis of autoreactive T cells [96]. Down-regulation of IRF1 signaling by IDMF may therefore have anti-inflammatory effects at multiple CNS and peripheral immune system sites (Figure 9).

Reactive astrocytosis is a stress-triggered process leading to a spectrum of astrocyte phenotypes with different functional capacities [26]. This spectrum has been conceptually characterized as polarization between inflammation-induced neurotoxic A1 and ischemia-induced neuroprotective A2 phenotypes [97]. Treatments that improve the clinical phenotype of EAE mouse models appear to promote A1 to A2 polarization [98,99,100], which in certain contexts may be mediated by NF-κB inhibition [98,101,102,103,104]. In this study, the best-supported A1 and A2 marker genes were not significantly altered by fumarate compounds (Figure S6A–D) and we did not see consistent directional changes among other genes linked to the A1 and A2 astrocyte phenotypes (Figure S6E,H,K). Indeed, IDMF both increased and decreased the expression of genes linked to the A1 phenotype (Figure S6L) and several such IDMF-decreased genes were localized to the lysosome and associated with catalytic activity (Figure S6N–P). Fumarate compounds thus appear to alter the expression of genes linked to reactive astrogliosis, although the overall pattern of change may not fit neatly within the A1 vs. A2 paradigm. This paradigm may be revised in future work if novel astrocyte sub-phenotypes can be established from studies of the neurodegenerative process [105]. 

The clinical use of DMF in MS patients has been limited by gastrointestinal (GI) adverse effects, which has prompted development of delayed-release formulations and novel agents such as DRF [22]. These adverse effects are predominantly due to the generation of methanol following first-pass hydrolysis in the small intestine [21]. Whereas orally administered DMF is hydrolyzed to MMF and methanol, DRF metabolism yields additional metabolites including metabolite 2-hydroxyethyl succinimide and RDC-8439 [23]. The generation of methanol following DRF hydrolysis appears to be a minor product, which may explain the improved GI tolerability of DRF as compared to MMF [23]. Our in vitro analysis shows that IDMF is hydrolyzed by carboxylesterase-2 to generate MMF and IMMF (Figure S8). This latter compound is structurally similar to IDMF. IDMF can therefore act as an MMF prodrug, similar to DRF, but may generate additional metabolites that may have IDMF-like bioactivity. Future in vivo studies are needed to evaluate systemic absorption of orally administered IDMF and the pharmacokinetics of its metabolites. This work can guide development of optimal delivery vehicles and further elucidate effects of IDMF and its metabolites on the CNS and peripheral immune cells.

## 4. Materials and Methods

### 4.1. Astrocyte Cell Cultures

Experiments were performed using human fetal astrocytes derived from cerebral cortex (cat. no. 882A-05f; Cell Applications, San Diego, CA, USA). Cells were grown to subconfluence in human astrocyte growth medium (cat. no. 820-500, Cell Applications). Stock solutions of MMF, DRF, and IDMF were prepared in DMSO (50 mM) and subsequent dilutions were made using distilled water. The experiment was performed using 12 replicate cultures, each of which was assigned to one of four experimental treatments (CTL, *n* = 3; MMF, *n* = 3, DRF, *n* = 3; IDMF, *n* = 3). CTL cells were treated with sterile distilled water. Test materials were added to subconfluent cultures at a concentration of 2.5 µM and cells were incubated for 24 h. 

### 4.2. RNA Processing

RNA was extracted following this incubation period using the RNAeasy Mini Plus kit (Qiagen, Hilden, Germany) and robotic Qiacube Connect station (Qiagen). Samples were shipped on dry ice to the Thermo Fisher Microarray Research Services Laboratory (Santa Clara, CA, USA) for transcriptome analysis. RNA quality analysis was performed upon sample receipt using the NanoDrop Lite (Thermo Fisher Scientific, Waltham, MA, USA). All samples had 260/280 nm absorbance ratios higher than 1.90, consistent with high-purity RNA (Figure S8M). 

### 4.3. Microarray Normalization and Quality Control

Gene expression profiling was performed using Clariom S Assays (*n* = 12 samples). Microarray pseudo-images generated from raw CEL files did not demonstrate evidence for spatial artifact with respect to 11 microarray samples (Figure S9B–L), although there was slight spatial artifact seen with respect to sample CTL-1 (Figure S9A, Supplementary Materials). However, eukaryotic hybridization spike-in controls showed expected trends for each array (Figure S9N), and likewise, 3 out of 4 poly-A RNA labeling controls spiked into RNA samples at varying concentrations showed expected trends (Figure S9O). Area under the curve (AUC) statistics evaluating the degree to which signals differed between exons and introns were close to 1.00 for all samples (≥0.93), consistent with good separation between these two probe set groups (Figure S9P). The distribution of probe-level model residuals was consistent among samples (Figure S9Q). The normalized unscaled standard error (NUSE) [106] median and interquartile range was higher for CTL-1 compared to other samples (Figure S9R,S). The relative log expression (RLE) [106] median was lower for CTL-1 compared to other samples (Figure S9T), whereas the RLE interquartile range was higher (Figure S9U). 

The robust multichip average (RMA) algorithm (R package: oligo; R function: rma) [107] was used to generate normalized expression intensities for 27,189 probes. Of these, 19,937 probes were annotated with a protein coding gene, although in some cases more than one probe was annotated with the same human gene symbol. In such cases, we selected the single probe for each gene having the highest average expression across the 12 samples. This yielded a final set of 18,088 probes included in further analyses, with each probe associated with a unique protein-coding human gene symbol. Hierarchical cluster analysis of the 12 samples was performed using expression estimates from these 18,088 genes. This showed that MMF samples grouped together, and likewise IDMF samples were similar to one another (Figure S9V). Cluster analysis did suggest that CTL-1 was a possible outlier (Figure S9V). However, CTL-1 did not emerge as a strong outlier when samples were plotted with respect to the first two principal component axes (Figure S9W). Consistent with this, sample CTL-1 was not a statistically significant outlier with respect to the first principal component axis (*p* = 0.18, maximum normalized residual test, i.e., Grubb’s test; Figure S9X). Differential expression testing was thus performed using all 12 samples, with exclusion of any genes meeting outlier criteria with respect to the CTL-1 sample (see below). 

### 4.4. Differential Expression Analysis

Tests for differential expression were performed with respect to three two-group comparisons (MMF vs. CTL, DRF vs. CTL, and IDMF vs. CTL). Differential expression tests were performed only for genes with detectable expression in at least 2 of the 6 samples involved in each two-sample comparison, with genes having an expression intensity above the 20th percentile on a given array considered to have detectable expression. Genes were also excluded if their standard deviation across all 12 samples was below the 5th percentile. Finally, given that sample CTL-1 had emerged as a borderline outlier in preliminary analyses (Figure S9), we excluded 132 genes for which expression estimates met outlier criteria with respect to the CTL-1 sample (i.e., absolute z-score greater than 2.50 with a value lower or higher than all other samples). After applying these filters, there remained 14,109, 14,847, and 14,118 genes that were included in differential expression analyses for the MMF vs. CTL, DRF vs. CTL, and IDMF vs. CTL comparisons, respectively. 

Differential expression analysis was performed using generalized least square linear models with empirical Bayes moderated t-statistics (R package: limma; R functions: lmFit and eBayes) [35]. To control the false discovery rate, raw *p*-values were adjusted for multiple hypothesis testing using the Benjamini–Hochberg method [108]. The distributions of raw *p*-values for the MMF vs. CTL and DRF vs. CTL comparisons were right-skewed, suggesting weak treatment effects (Figure S1A,B). In contrast, the raw *p*-value distribution for the IDMF vs. CTL comparison was left-skewed, consistent with a stronger treatment effect (Figure S1C). The stronger treatment effect of IDMF compared to MMF and DRF could also be discerned from QQ plots of moderated T statistics (Figure S1D–F) and from raw *p*-value empirical CDFs (Figure S1G).

Applying a stringent significant threshold (FDR < 0.10 with FC > 1.25 or FC < 0.80), we identified 23 DEGs with respect to the IDMF vs. CTL comparison but did not identify any DEGs with respect to the MMF vs. CTL or DRF vs. CTL comparisons. In further analyses, therefore, we identified DEGs based upon a less stringent threshold (*p* < 0.05 with FC > 1.25 or FC < 0.80) (Figure S1H–J). We identified approximately twice as many MMF-increased DEGs compared to MMF-decreased DEGs (Figure S1H,K,N), but the numbers of increased and decreased DEGs were similar for both other comparisons (Figure S1I,J,L,M,O,P). For all comparisons, fold-change estimates were not biased among genes having low or high expression (Figure S1N–P).

### 4.5. Gene Expression Module Analysis

Gene expression modules were evaluated as an alternative to focusing on individual genes. This allowed us to identify differentially expressed modules (DEMs), defined as groups of co-expressed genes consistently altered in the same direction by each fumarate compound [109]. Expression modules were identified based upon an independent set of 24 microarray samples generated from prior studies of iPSC-derived astrocytes treated with TNF-α or α-synuclein proteins (GSE166768 and GSE166769) [110]. This study had utilized the same microarray platform design as in our current study (i.e., Clariom S Assays). Module analyses included only matching probes associated with 12,986 genes having detectable expression such that they were included in each differential expression comparison (MMF vs. CTL, DRF vs. CTL, IDMF vs. CTL). 

Raw CEL files were normalized as above using the RMA algorithm [107] and the normalized expression intensity of each gene was centered (by subtracting its average expression across the 24 samples). The resulting normalized/centered expression values were then scaled to have the same mean (0) and standard deviation (1) across all array samples. The 12,986 genes were then clustered using a hierarchical approach with Euclidean distance and average linkage (R function: hclust). The resulting dendrogram was analyzed using a variable height branch pruning technique to detect 233 gene modules (R package: dynamicTreeCut; function: cutreeDynamicTree) [111]. The size of gene modules ranged from 25 to 196 genes. Approximately 7% of genes (929) were not assigned to any module and were thus excluded from further analyses. The 233 modules were labeled using the module size and the single member gene associated with the largest number of Gene Ontology terms. For example, module DCTN1-74 contained 74 genes and of these *DCTN1* was associated with the most Gene Ontology terms. To assess differential module expression, a two-sample Wilcoxon rank sum test was used to assess whether FC estimates for module genes differed significantly from all other detectable genes [109]. To correct for multiple hypothesis testing among the 233 modules, raw *p*-values were adjusted using the Benjamini–Hochberg approach [108].

### 4.6. DEG Analysis and Annotation

DEGs analysis was performed using enrichment analyses with annotations drawn from several existing databases, including Gene Ontology (GO) and the Kyoto Encyclopedia of Genes and Genomes (KEGG) [32,112]. Annotation-based gene set enrichment analysis was performed using a conditional hypergeometric test (R package: GOstats; function: hyperGTest) [113]. We used pre-compiled sets of target genes to evaluate the effects of fumarate compounds on the NRF2 and NF-kB pathways [37,38]. Effects of fumarates on the niacin-HCAR2 pathway were evaluated using data from a prior microarray study in which monocytes were treated with niacin (100 μM) for 6 h (GSE103381) [41]. Enrichment of motifs in DEG upstream regions was assessed using semiparametric generalized additive logistic models [36] with a pre-compiled set of 2935 empirically-determined binding sites (position weight matrix models) associated with mouse or human transcription factors or unconventional DNA binding proteins (uDBPs) [114]. Genes linked to MS via GWA studies were identified using the NHGRI-EBI GWAS catalog [33]. A more comprehensive set of MS-associated genes was identified using an aggregative approach described previously [25], which incorporated MS-associated genes included in 7 database sources (i.e., NHGRI-EBI GWAS catalog, MeSH, disease ontology, DisGeNet, MalaCards, and KEGG) [28,29,30,31,32,33]. DEGs identified in this study were further compared with those with altered expression in astrocytes from normal appearing white matter within brains obtained from MS patients (GSE83670) [34]. Finally, DEGs were compared against those previously identified as markers of A1 and A2 astrogliosis [42], which were identified as the human orthologues of genes previously identified from microarray analyses of astrocytes isolated from brains of mice subjected to inflammatory or ischemic injury, respectively [44].

### 4.7. Real-Time Quantitative PCR (RT-PCR)

The expression of selected genes was further evaluated using RT-PCR. cDNA was prepared using the AzuraQuant cDNA kit (Azura Genomics, Raynham, MA, USA) and PCR reactions were performed using the BioRad iCycler iQ Detection System with Fast Green qPCR Master Mix—Fluor (Azura Genomics). PCR primers were purchased from Realtimeprimers (Elkins Park, PA, USA). The ΔΔCt method [115] was used to estimate fold changes with cycle threshold values normalized based upon the expression of glyceraldehyde-3-phosphate dehydrogenase (*GAPDH*).

### 4.8. NF-κB Reporter Assay

Experiments were performed using human fetal astrocytes (HA) derived from cerebral cortex (cat no. 882A-05f; Cell Applications, San Diego, CA, USA) grown to subconfluence for 48 h in HA growth medium (cat. no. 820-500, Cell Applications) in a 96-well black wall tissue culture plate. HA growth medium was replaced by Opti-MEM (Thermo Fisher Scientific, Waltham, MA, USA) on the day of transfection and cells were transfected using NF-κB reporter assay firefly/Renilla luciferase constructs (cat. no. 60616; BPS Bioscience, San Diego, CA, USA) combined with plasmid transfection reagent (Santa Cruz Biotechnology, Dallas, TX, USA) for 18 h. The medium was changed back to HA growth medium and TNF (20 ng/mL) (*n* = 6 replicates) or TNF (20 ng/mL) + IDMF (2.5 µM) (*n* = 3) was added. NF-κB induction was quantified 6 h later using a dual-luciferase reporter assay system (cat. no. E1960; Promega, Madison, WI, USA). Signal quantification was obtained using a Thermo Fisher Scientific Luminoskan Ascent Microplate Luminometer. The instrument had passed DLReady™ validation at Promega Corporation and was certified as dual-luciferase assay-ready. NF-κB inhibition was quantified based upon the ratio between luciferase and Renilla signals, with the latter signal providing an internal control for transfection efficiency and cell viability. 

### 4.9. Fatty Acid Ester Hydrolysis of IDMF with Carboxylesterase-2

IDMF (1 mM) was dissolved in DMSO (2%) and ethylene glycol (2%) in 20 mM HEPES (pH 7.4). We added 20 μL of enzyme (1 mg/mL) and the solution was incubated at 37 °C for 15, 30, 60, and 120 min. The reaction was stopped using Methanol. IDMF and MMF were quantified using a UV detector at a wavelength of 210 nm. HPLC was performed with 0.1% formic acid in water as the weak solvent and methanol as the strong solvent. The analysis was performed using a Luna C18 4.6 mm × 100 mm column with a flow rate of 1.0 mL/min (Phenomenex, Torrance, CA, USA). 

The molecular weight of IMMF was determined using the API3000 triple-quadrupole mass spectrometer (AB Sciex, Framingham, MA, USA) and electrospray ionization (ESI). The Q1 scan was performed from 100–1500 Da with negative mode. MS analysis conditions were as follows: curtain gas (10 arbitrary units), nebulizer gas (14 arbitrary units), ion spray voltage (−4500 V), channel electron multiplier (CEM) at 2800 V. Declustering and entrance potentials were −130 V and −10 V, respectively.

## 5. Conclusions

Fumarate esters have been approved as treatments for RRMS and psoriasis but work is now underway to explore their utility as treatments for diverse other conditions, such as chronic pain, Parkinson’s disease, Alzheimer’s disease, stroke, glioblastoma multiforme, chronic lymphocytic leukemia, cutaneous T cell lymphoma, obstructive sleep apnea, rheumatoid arthritis, and atherosclerosis [27,116,117]. Development of next-generation fumarate compounds with improved properties is also ongoing although little work has been done so far to provide a side-by-side comparison of their effects. This study used transcriptomics to show that fumarates have differing effects on the antioxidant and inflammatory genes acting downstream of the NRF2, NF-κB, IRF1, and HCAR2 (GPR109A) pathways (Figure 9 and Table 1). Likewise, fumarates had differing effects on the expression of key genes that have been linked to multiple sclerosis (e.g., *TNFAIP3*, *CXCL8*, *IL6*, and *IRF1*). These findings highlight distinguishing features of fumarate compounds that may be consequential for their use as treatments for multiple sclerosis or other autoimmune diseases. This side-by-side comparison helps to define functional properties of different fumarate compounds with the goal of developing new therapies to meet the needs of MS patients.

## Figures and Tables

**Table 1:** Summary table. The table lists the number of DEGs identified (*p* < 0.05 with FC > 1.25 or FC < 0.80), top biological process (BP) terms most strongly enriched among DEGs, top sequence motifs enriched in DEG 5 kb upstream regions, DEGs correspondingly regulated by NRF2 and niacin, DEGs inversely regulated by NF-κB, IRF factors associated with enriched motifs in DEG upstream regions, A1 astrocyte marker down-regulated DEGs, MS-associated DEGs, and DEGs near MS GWAS loci. Results are summarized for increased DEGs (▲), decreased DEGs (▼), and the combined set of increased and decreased DEGs (▲ + ▼). See footnotes for further details.

	DEG Group	MMF	DRF	IDMF
No. DEGs identified ^1^	▲	308	181	673
	▼	142	198	903
	▲ + ▼	450	379	1576
No. DEMs identified ^2^	▲	14	40	71
	▼	11	42	70
	▲ + ▼	25	82	141
Top GO BP term ^3^	▲	Oxidation–reduction	Circadian behavior	Smell stimulus detection
	▼	Mast cell degranulation	Macromolecule modification	Cellular metabolism
Top motif ^4^	▲	5-AAATT/AATTT-3	5-CTAGCA/TGCTAG-3	5-CATG/CATG-3
	▼	5-GGGCG/CGCCC-3	5-TAATT/AATTA-3	5-TAAT/ATTA-3
NRF2-increased DEGs ^5^	▲	*NQO1*, *SLC3A2*, *POP4*	*TXNRD1*, *GCLC*, *PTGR1*	*HMOX1*, *GSR*, *SLC7A11*, *SLC6A6*
NRF2-decreased DEGs ^5^	▼	None	None	*LTBP1*, *HNRNPU*, *PCYOX1*
NF-kB-increased DEGs ^6^	▼	*KLHL18*	*ZZZ3*, *RIOK1*, *RHOBTB3*	*NFKBIA*, *TNFAIP3*, *IRF1*, *NEDD4L*
NF-kB-decreased DEGs ^6^	▲	*FOSB*, *SLC3A2*	None	*EGR1*
Niacin-increased DEGs ^7^	▲	*CCNL1*	None	*SLC22A14*
Niacin-decreased DEGs ^7^	▼	None	*CNIH1*, *ALG8*	*HIGD1A*, *DHX9*, *AKT2*
IRF motif enrichment ^8^	▲	None	IRF1, IRF6	IRF1, IRF4, IRF6, IRF7
A1 astrocyte DEGs ^9^	▼	*CD82*, *CYP27A1*	*TXNIP*	*TERF1*, *GOT1*, *BPGM*, *SORT1*
MS-associated DEGs ^10^	▲	*CD47*, *SLC1A3*	*HLA-DRB5*, *CNR2*, *PPARA*	*HMOX1*, *HLA-DRB5*, *BTNL2*
	▼	*MPZ*	*GLO1*, *IFNAR2*	*TNFAIP3*, *IFNAR2*, *ZNF433*
DEGs near MS GWAS loci ^11^	▲	*POPDC3*, *COG6*, *VWA8*	*ALDH1L1*, *ACTRT3*, *ELAC1*	*ALDH1L1*, *MLANA*, *SLC2A4RG*
	▼	*PRR5L*, *PLEC*, *ZC3H4*	*PHGDH*, *PFDN4*, *EPPK1*	*ZBTB38*, *ZNF433*, *STAT3*

^1^ DEG criteria: *p* < 0.05 with FC > 1.25 or FC < 0.80. ^2^ DEM criteria: FDR < 0.05 (two-sample Wilcoxon rank sum test) (Figure 3). ^3^ GO BP term most strongly enriched among DEGs (Figure 2). ^4^ Motif most strongly enriched in regions 5 KB upstream of DEGs (Figure S2). ^5^ NRF2-regulated genes correspondingly altered by each fumarate (Figure 4). ^6^ NF-κB-regulated genes inversely altered by each fumarate (Figure 5). ^7^ Niacin-regulated genes correspondingly altered by each fumarate (Figure S4). ^8^ IRF transcription factors associated with recognition sequences enriched in sequences upstream of DEGs (Figure S5). ^9^ A1 neurotoxic astrocyte marker genes down-regulated by each fumarate (Figure S6). ^10^ MS-associated DEGs associated with each fumarate (Figure 6). ^11^ DEGs overlapping with or nearest to MS-associated GWAS loci (Figure 7).

## Data Availability

The whole genome microarray data have been submitted to Gene Expression Omnibus (GEO) and are available under the accession GSE200539.

## Supplementary Materials

The following supporting information can be downloaded at: https://www.mdpi.com/article/10.3390/ph15040461/s1, Figure S1: Differential expression analyses; Figure S2: Motifs enriched in 5 kb sequences upstream of DEGs; Figure S3: NF-κB motif enrichment; Figure S4: Niacin-HCAR2 pathway genes; Figure S5: Interferon regulatory factor (IRF) motif enrichment; Figure S6: Genes associated with A1/A2 reactive astrocyte polarization; Figure S7: Genes altered in MS patient astrocytes (GSE83670) and their overlap with DEGs; Figure S8: In vitro kinetics of IDMF hydrolysis by carboxylesterase-2; Figure S9: Microarray quality control; Table S1. MMF-increased genes (*p* < 0.05 with FC > 1.25); Table S2. MMF-decreased genes (*p* < 0.05 with FC < 0.80); Table S3. DRF-increased genes (*p* < 0.05 with FC > 1.25); Table S4. DRF-decreased genes (*p* < 0.05 with FC < 0.80); Table S5. IDMF-increased genes (*p* < 0.05 with FC > 1.25); Table S6. IDMF-decreased genes (*p* < 0.05 with FC < 0.80).
