# Supplementary material for: Transcriptomic Analysis of Fumarate Compounds Identifies Unique Effects of Isosorbide Di-(Methyl Fumarate) on NRF2, NF-kappaB and IRF1 Pathway Genes
Source: Pharmaceuticals (Basel). 2022 Apr 11;15(4):461. doi: 10.3390/ph15040461 (PMC9026097; doi:10.3390/ph15040461)
Supplement: Supplementary file 1 [file pharmaceuticals-15-00461-s001.zip › pharmaceuticals-1637159-supplementary.pdf]

Supplemental Figures and Tables

Transcriptomic analysis of fumarate compounds identifies unique effects of isosorbide di-(methyl fumarate) on NRF2, NF-kappaB and IRF1 pathway genes

William R. Swindell[1*], Krzysztof Bojanowski[2], Ratan K. Chaudhuri[3]

[1]The Jewish Hospital, Department of Internal Medicine, Cincinnati, OH, 45236, USA; ws277814@ohio.edu
[2]Sunny BioDiscovery Inc., Santa Paula, CA, USA; Symbionyx Pharmaceuticals Inc., Boonton, NJ, USA; kbojanowski@sunnybiodiscovery.com
[3]Sytheon Ltd., Boonton, NJ, USA; Symbionyx Pharmaceuticals Inc., Boonton, NJ, USA; ratan@sytheonltd.com
[*]Corresponding Author.

Supplemental figure legends

Figure S1. Differential expression analyses. (A-C) P-value distributions. Plots show the raw p-value distribution among all genes for each differential expression analysis. The number of DEGs identified is shown (top margin; $P < 0.05$ with $FC > 1.25$ or $FC < 0.80$). (D-F) QQ plots for moderated T statistics. Deviation of sample quantiles (black points) from a straight line (null) reflects more differential expression among genes tested. (G) P-value empirical cumulative distribution functions (CDFs). CDF curves are shown for each comparison (to the CTL treatment). Deviation from a straight line indicates a non-uniform p-value distribution. (H-J) Number of DEGs identified at varying FC thresholds ($P < 0.05$; yellow arrow: $FC > 1.25$ or $FC < 0.80$). (K-M) Volcano plots. The -log10-transformed p-value (vertical axis) is plotted with respect to the estimated FC (horizontal axis). (N-P) MA plots. The FC (vertical axis) is plotted against average expression (horizontal axis) for each gene. The solid green line is the LOESS curve fit. In (K) - (P), red and blue symbols correspond to genes significantly increased or decreased, respectively ($P < 0.05$, $FC > 1.25$ of $FC < 0.80$).

Figure S2. Motifs enriched in 5 kb sequences upstream of DEGs. (A) Z statistic interpretation. Z statistics were calculated using semiparametric generalized additive logistic models. Positive Z statistics indicate a relative increase in the frequency of a binding site in DEG upstream sequences (as compared to all non-DEGs). Negative Z statistics indicate a relative decrease in the frequency of a binding site in DEG upstream sequences (as compared to all non-DEGs). (B - G) Top motifs enriched in DEG upstream sequences. The primary and reverse complement sequence logos are shown for each motif along with the enrichment Z statistic (*$P < 0.05$; **$FDR < 0.05$).

Figure S3. NF-κB motif enrichment. (A) NF-κB motif sequence logos. (B, C) NF-κB motif enrichment. The Z statistic (vertical axis) quantifies enrichment of each motif in 5 kb upstream regions (positive value: motif enrichment; negative value: motif depletion; black asterisk: $P < 0.05$; red asterisk: $FDR < 0.05$). (D) NF-κB subunits. NF-κB signaling occurs through both canonical and non-canonical pathways. The canonical pathway is activated by stimuli such as TNF-α and IL-1 and leads to formation of p50/p65 dimers. The non-canonical pathway is triggered by stimuli such as lymphotoxin beta (LT-β) and B-cell activating factor (BAFF) to promote p52/RELB dimer formation. (E – I) NF-κB subunit gene expression. Average expression (log2 scale) is shown for each treatment (± 1 standard error; *$P < 0.05$, comparison to CTL treatment). Expression is normalized to the average value in the CTL treatment for each gene.

Figure S4. Niacin-HCAR2 pathway genes. (A) *HCAR2*. (B) *PTGDS*. (C) *SREBF1*. (D) *ABCA1*. In (A) - (D), average expression (log2 scale) is shown for each treatment (± 1 standard error; *$P < 0.05$, comparison to CTL treatment). Expression is normalized to the average value in the CTL treatment for each gene. (E) Niacin-activated genes. (F) Niacin-suppressed genes. In (E) and (F), the value of $|FC|_{max}$ was calculated for each gene, where $|FC|_{max}$ is defined as $\max[\text{abs}(FC_{MMF}, FC_{DRF}, FC_{IDMF})]$, and the 23 niacin-activated or niacin-suppressed genes with highest value of $|FC|_{max}$ are shown. Non-black gene labels are used if there is significant differential expression ($P < 0.05$) for any of the three comparisons, in which case the label color matches the comparison associated with the lowest differential expression p-value. (G, H, K, L,

O, P) FC estimates for niacin-activated genes. FC estimates are plotted and the proportion of fumarate-increased (red) fumarate-decreased (blue) genes is shown (P-value: Fisher's exact test). The percentage of increased/decreased genes is also indicated (see legend). (I, J, M, N, Q, R) The cumulative overlap is shown between niacin-activated genes and genes ranked based upon their response to each fumarate compound. A positive area statistic denotes enrichment among fumarate-increased genes, and a negative area statistic indicates enrichment among fumarate-decreased genes (P-value: Wilcoxon rank sum test).

Figure S5. Interferon regulatory factor (IRF) motif enrichment. (A) IRF motif sequence logos. (B, C) IRF motif enrichment. The Z statistic (vertical axis) quantifies enrichment of each motif in 5 kb upstream regions (positive value: motif enrichment; negative value: motif depletion; black asterisk: $P < 0.05$; red asterisk: $FDR < 0.05$). (D-F, H-J) IRF factor gene expression. Average expression (log2 scale) is shown for each treatment (± 1 standard error; *$P < 0.05$, comparison to CTL treatment). Expression is normalized to the average value in the CTL treatment for each gene. (G) IRF1 predicted targets (motif #1 in part A). (K) IRF1 binding sites in regions upstream of IDMF-decreased genes. Motif sequence matches are shown at varying degrees of stringency (60-90%, see legend). The FC (IDMF/CTL) and differential expression p-value are shown (right margin).

Figure S6. Genes associated with A1/A2 reactive astrocyte polarization. (A) *C3*. (B) *GBP2*. (C) *S100A10*. (D) *TGFB1*. In (A) - (D), average expression ($\log_2$ scale) is shown for each treatment (± 1 standard error; *$P < 0.05$, comparison to CTL treatment). Expression is normalized to the average value in the CTL treatment for each gene. (E, H, K) Median expression of genes associated with the A1 phenotype ($n = 161$ genes), A2 phenotype ($n = 75$), and pan-reactive astrogliosis (A1 + A2) ($n = 127$). Boxes outline FC estimates for the middle 50% of genes (middle line: median) and whiskers span the 10th to 90th percentiles. (F, G, I, J, L, M) Genes associated with A1 and A2 phenotypes. Figures show marker genes most strongly altered by (F, G) MMF, (I, J) DRF, or (L, M) IDMF. Genes shown in red and blue font are significantly up- and down-regulated by (F, G) MMF, (I, J) DRF, or (L, M) IDMF, respectively. (N, O, P) Gene ontology (GO) biological process (BP), molecular function (MF), and cell component (CC) terms associated with IDMF-decreased A1 astrocyte marker genes ($P < 0.05$). Enrichment is shown on the horizontal axis ($-\text{Log}_{10}$(p-value), conditional Fisher's Exact Test) and the number of genes associated with each term is given in parentheses (left margin). The single gene associated with each term and most strongly down-regulated by IDMF is listed within each figure.

Figure S7. Genes altered in MS patient astrocytes (GSE83670) and their overlap with DEGs. (A, E, I) Fold-change scatterplots. Plots compare FC estimates from the current analysis (horizontal axis) with those from MS astrocytes (vertical axis). Each point represents an individual gene. The proportion of genes within each quadrant is indicated in the top margin (red font: $P < 0.05$, Fisher's Exact Test). The yellow ellipse outlines the middle 90% of genes closest to the bivariate mean. (B, C, F, G, J, K) Venn diagrams. Diagrams show the overlap between genes altered by MMF, DRF or IDMF and those altered in astrocytes from MS patients ($P < 0.05$, $FC > 1.25$ or $FC < 0.80$). (D, H, L) GSEA analysis. Genes were ranked based upon expression change in MS astrocytes (horizontal axis) and cumulative overlap is shown (vertical axis) with respect to the top 100 genes increased (red) or decreased (blue) by each compound.

The area between each cumulative overlap curve and the diagonal is indicated (Area > 0: enrichment among MS-increased genes; Area < 0: enrichment among MS-decreased genes; p-value: two-sample Mann Whitney U test). (M, N) Genes altered in the same direction by IDMF ($P < 0.05$) and in astrocytes from MS patients ($P < 0.15$). (O, P) GO BP terms enriched among genes (O) increased by IDMF and in MS astrocytes or (P) decreased by IDMF and in MS astrocytes. The number of genes associated with each term is given in parentheses (left margin) and example genes for each term are listed within each figure.

Figure S8. *In vitro* kinetics of IDMF hydrolysis by carboxylesterase-2. (A) High-performance liquid chromatography (HPLC) chromatograms. Overlaid chromatograms are shown for IDMF and the metabolites MMF and IMMF. (B - D) Calibration curves. Calibration curves are shown for IDMF and its MMF and IMMF derivatives. The least-squares regression line is shown (dotted line) with associated R2 value (top-left) and estimated regression equation (top margin). (E) IDMF degradation and product accumulation during hydrolysis. Estimated concentrations for each compound following hydrolysis are shown over two hours.

Figure S9. Microarray quality control. (A – L) Microarray pseudoimages. Darker colors correspond to regions of increased hybridization. (M) 260/280 absorbance ratios. (N) Eukaryotic hybridization controls. Controls were spiked into the hybridization cocktail at different relative concentrations (cre > BioD > BioC > BioB). The average expression of control probes is shown for each array. (O) Labeling controls. Poly-A RNA control probes target *B. subtilis* genes absent in eukaryotic samples, which were spiked into the RNA sample at varying concentrations during sample preparation (dap > thr > phe > lys). (P) Positive vs. negative AUC. Area under the curve (AUC) values were calculated from receiver operator curves (ROC), which were generated by evaluating how well probe set signals separate exons (positive control) from introns (negative control). A value of 1.00 is consistent with complete separation between exon- and intron-derived signals, whereas a value of 0.50 suggests overlap between these two probe set groups. (Q) Probe-level model (PLM) residuals. Boxplots outline the middle 50% of residual values for each array (whiskers: 10th to 90th percentiles). (R) Normalized unscaled standard error (NUSE) median. (S) NUSE interquartile range (IQR). (T) Relative log expression (RLE) median. (U) RLE IQR. (V) Hierarchical cluster analysis. Samples were clustered based upon the Euclidean distance with groups joined by average linkage. (W) Principal component (PC) analysis. Samples are plotted with respect to the first two PC axes. The percentage of variation accounted for by each PC axis is indicated in axis labels. (X) PC axis 1 standard score (Z-score). The p-value obtained from the univariate maximum normalized residual test for outliers is shown (i.e., Grubb’s test).

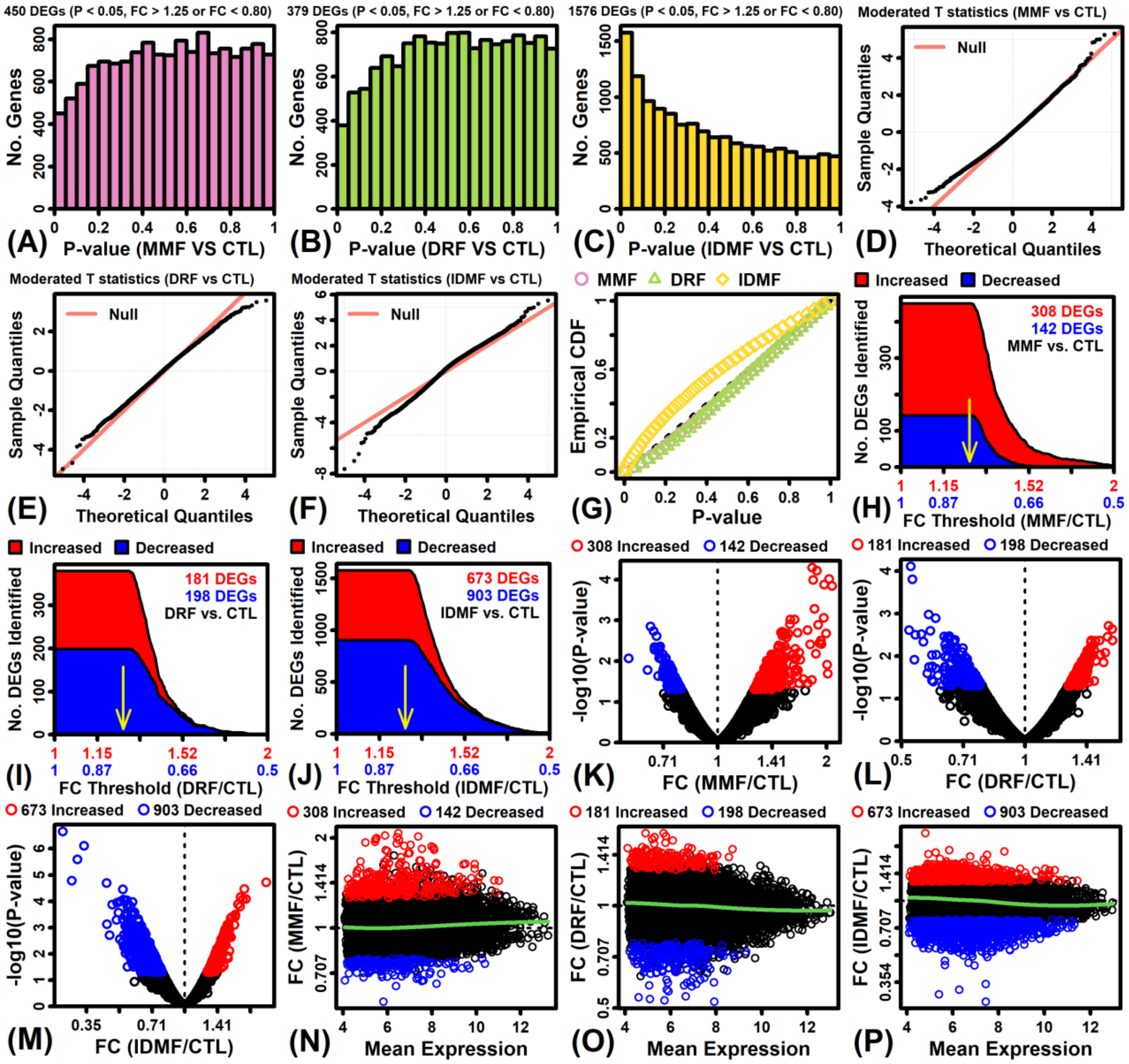

Figure S1. Differential expression analyses.

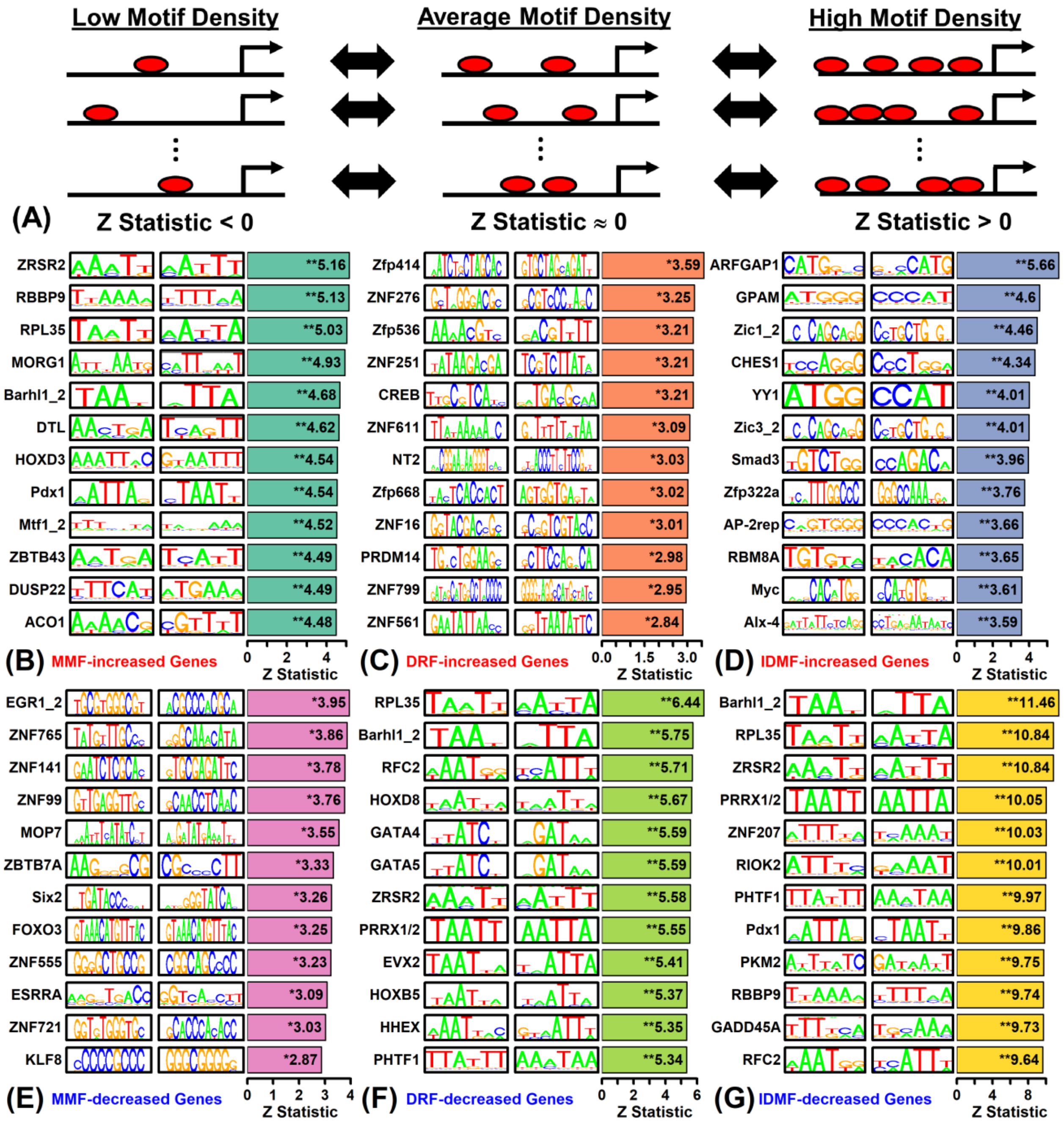

Figure S2. Motifs enriched in 5 kb sequences upstream of DEGs.

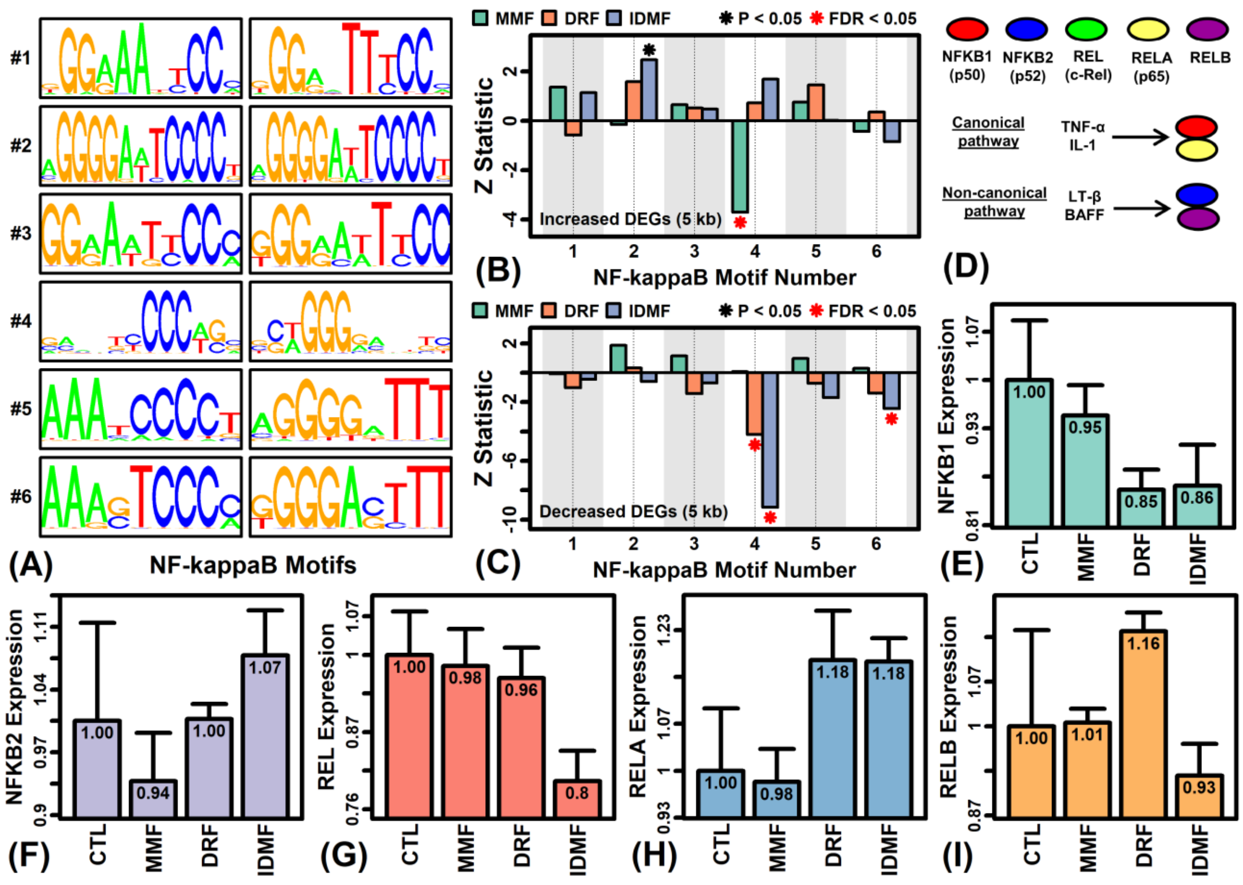

Figure S3. NF-κB motif enrichment.

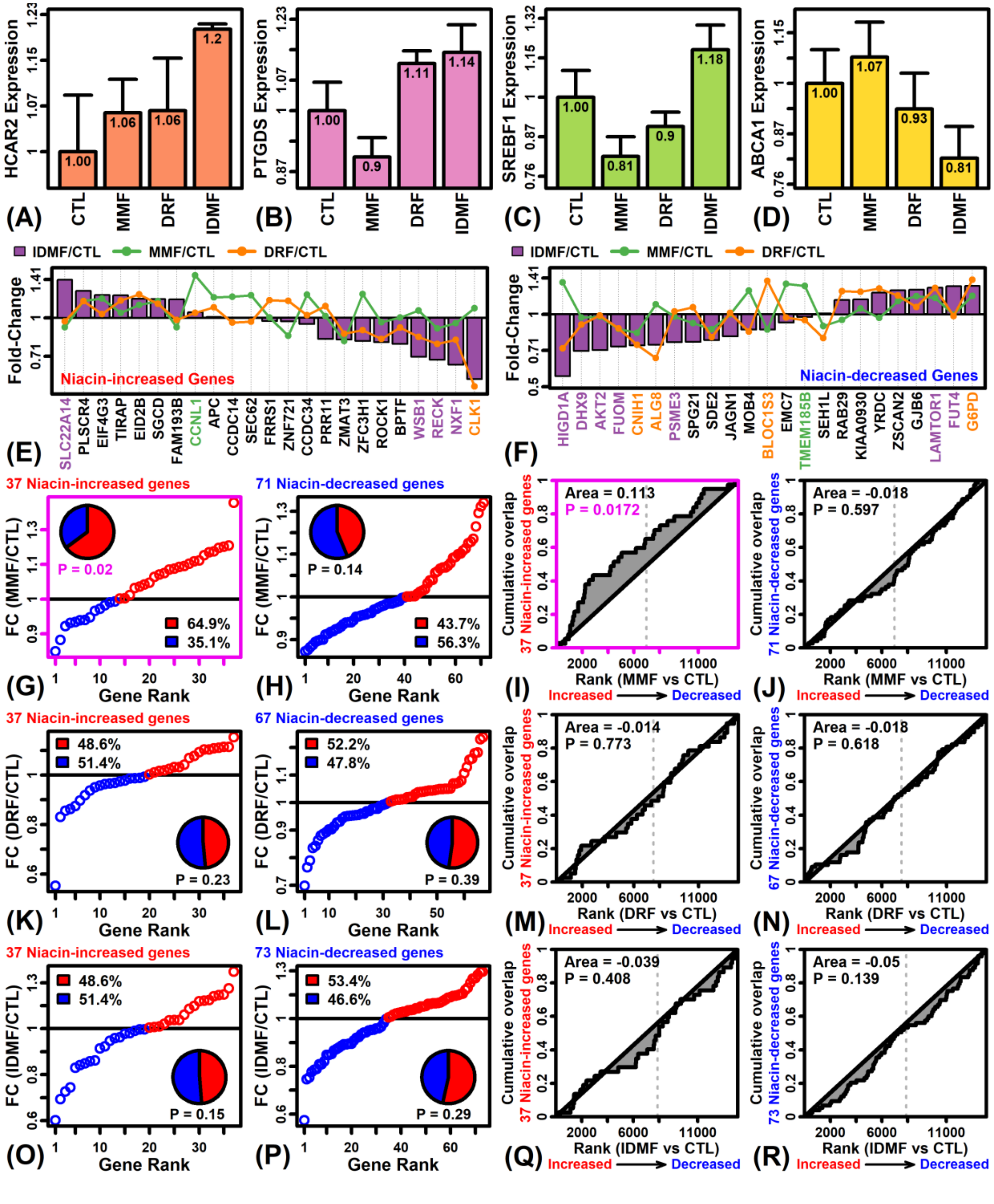

Figure S4. Niacin-HCAR2 pathway genes.

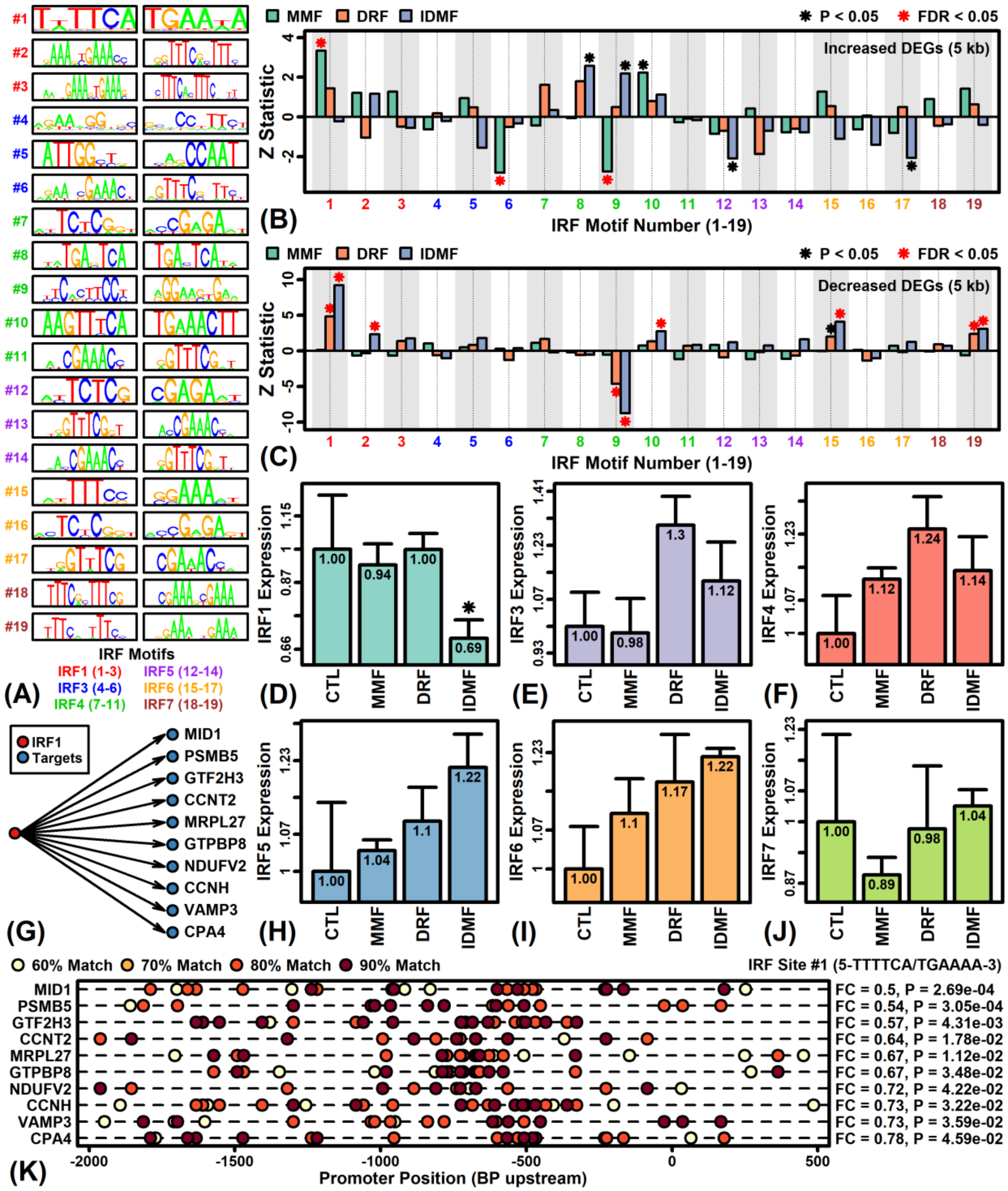

Figure S5. Interferon regulatory factor (IRF) motif enrichment.

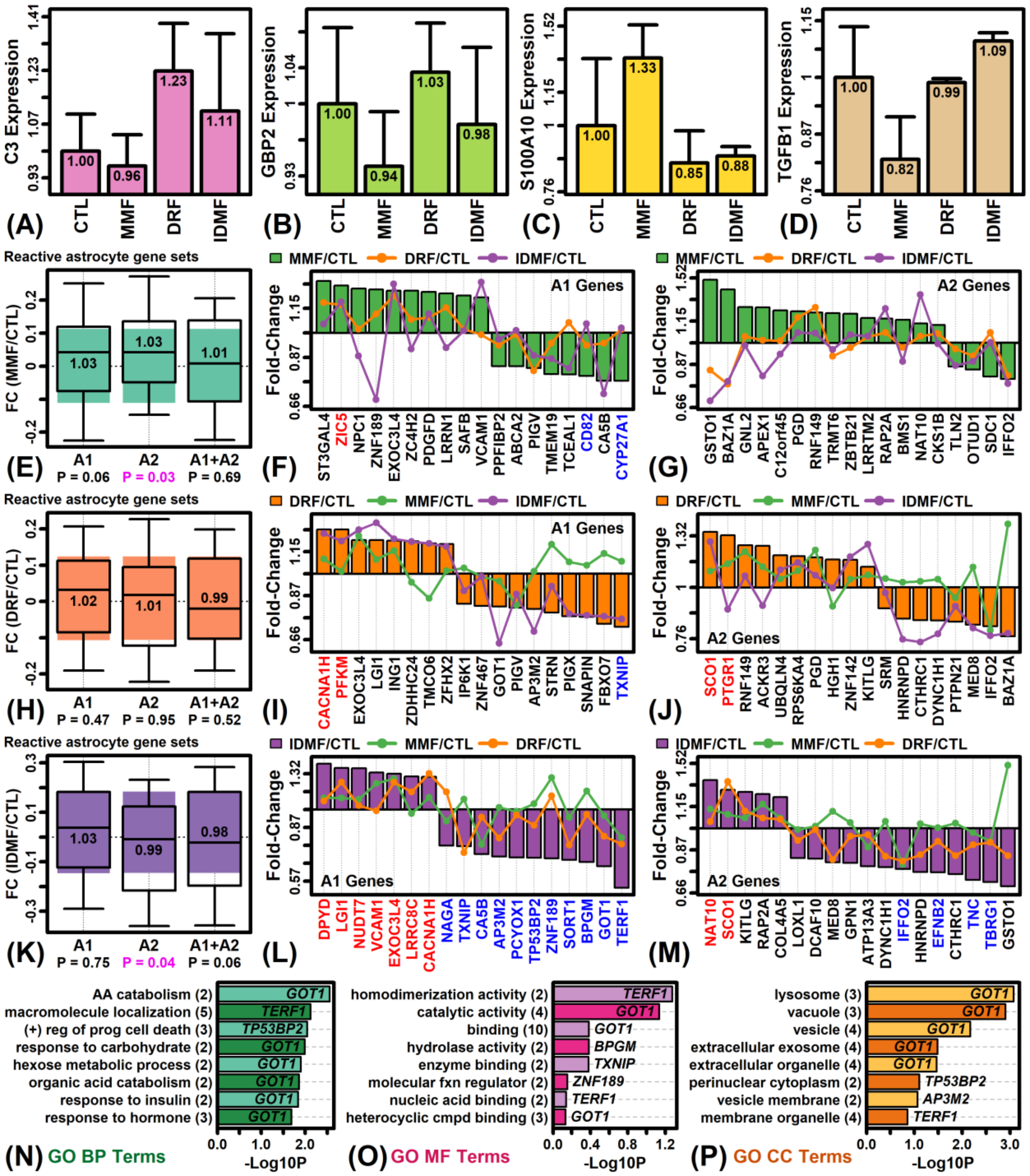

Figure S6. Genes associated with A1/A2 reactive astrocyte polarization.

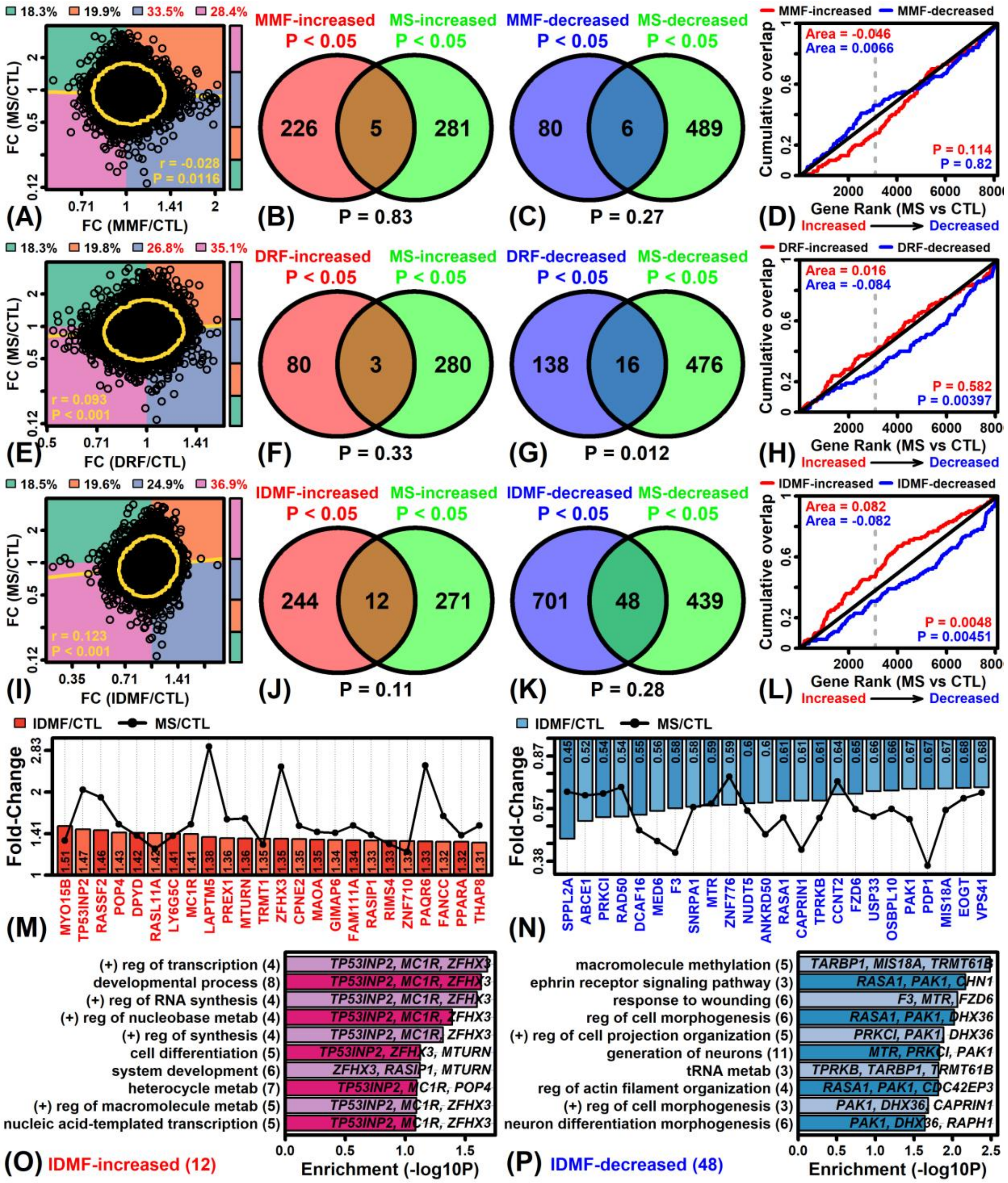

Figure S7. Genes altered in MS patient astrocytes (GSE83670) and their overlap with DEGs.

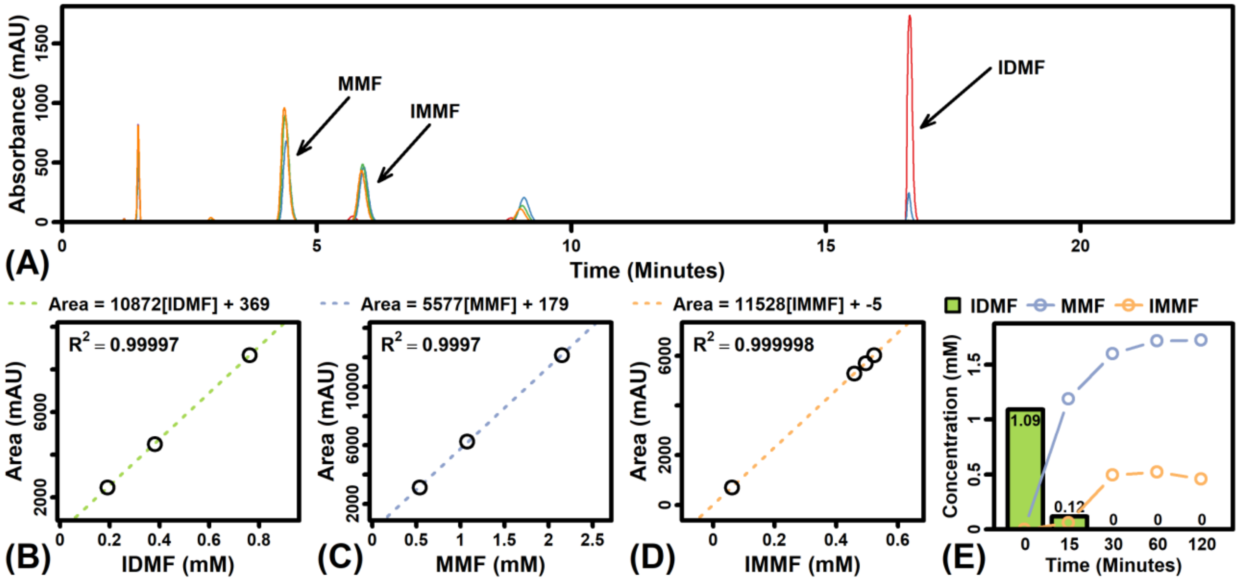

Figure S8. ***In vitro*** **kinetics of IDMF hydrolysis by carboxylesterase-2.**

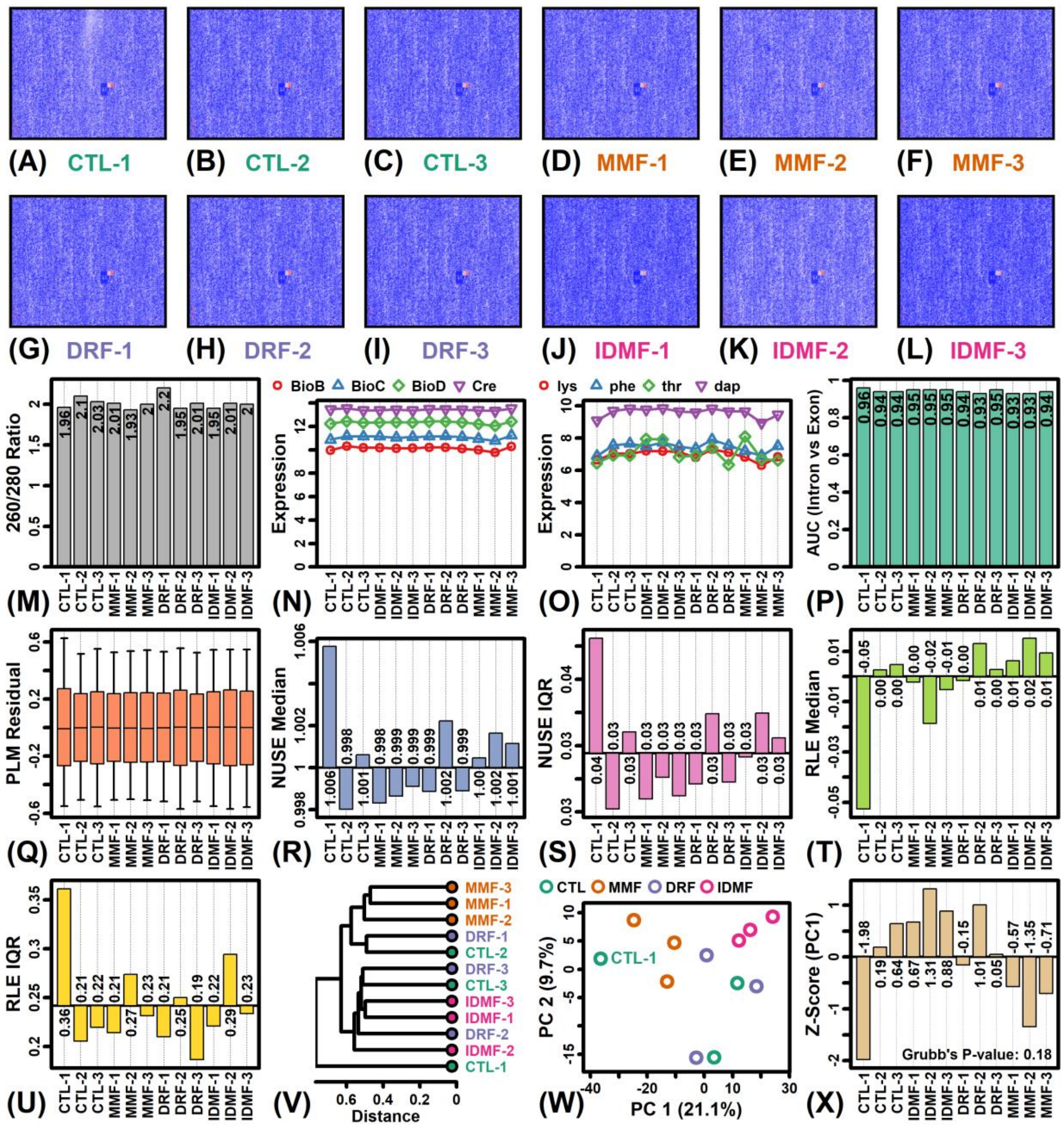

Figure S9. Microarray quality control.

Table S1. MMF-increased genes (P < 0.05 with FC > 1.25). The table lists 308 MMF-increased genes ranked by estimated fold-change (FC) (MMF/CTL). The gene symbol and description are shown. The raw p-value is listed in the final column (generalized least square linear models with moderated t-statistics; *n* = 3 samples per group).

Symbol	Description	FC	P-value
BSCL2	Berardinelli-Seip congenital lipodystrophy 2 (seipin)	2.07	0.000143
C1orf54	chromosome 1 open reading frame 54	2.03	0.0000967
PSMA3	proteasome subunit alpha 3	2.02	0.0204
COA5	cytochrome c oxidase assembly factor 5	2.01	0.0123
PROSER1	proline and serine rich 1	2.00	0.00211
NEFM	neurofilament, medium polypeptide	1.97	0.000135
LSAMP	limbic system-associated membrane protein	1.97	0.000873
PARN	poly(A)-specific ribonuclease	1.94	0.00384
UPF3B	UPF3 regulator of nonsense transcripts homolog B (yeast)	1.93	0.00309
NTPCR	nucleoside-triphosphatase, cancer-related	1.91	0.00363
ZNF106	zinc finger protein 106	1.90	0.0013
SCPEP1	serine carboxypeptidase 1	1.88	0.0000603
CNIH4	cornichon family AMPA receptor auxiliary protein 4	1.87	0.00665
FOSB	FBJ murine osteosarcoma viral oncogene homolog B	1.85	0.000523
ATF3	activating transcription factor 3	1.84	0.000103
NFATC2	nuclear factor of activated T-cells, cytoplasmic, calcineurin-dependent 2	1.83	0.0000509
HADH	hydroxyacyl-CoA dehydrogenase	1.82	0.0291
LPCAT3	lysophosphatidylcholine acyltransferase 3	1.80	0.00394
CD47	CD47 molecule	1.78	0.035
SRPRB	signal recognition particle receptor, B subunit	1.77	0.00173
EEF1D	eukaryotic translation elongation factor 1 delta (guanine nucleotide exchange	1.72	0.0275
NDUFAF5	NADH dehydrogenase (ubiquinone) complex I, assembly factor 5	1.69	0.0092
RRAGC	Ras-related GTP binding C	1.68	0.0354
MED31	mediator complex subunit 31	1.68	0.00612
VWA8	von Willebrand factor A domain containing 8	1.67	0.017
VPS18	VPS18 CORVET/HOPS core subunit	1.66	0.00201
PLAA	phospholipase A2-activating protein	1.66	0.0234
PHLDB1	pleckstrin homology-like domain, family B, member 1	1.65	0.00822
PIK3C3	phosphatidylinositol 3-kinase, catalytic subunit type 3	1.65	0.016
RBMS1	RNA binding motif, single stranded interacting protein 1	1.64	0.00499
LAMTOR5	late endosomal/lysosomal adaptor, MAPK and MTOR activator 5	1.64	0.0185
CENPP	centromere protein P	1.63	0.00096
NDUFB3	NADH dehydrogenase (ubiquinone) 1 beta subcomplex, 3, 12kDa	1.63	0.00846
MRPS16	mitochondrial ribosomal protein S16	1.63	0.00893
TTLL7	tubulin tyrosine ligase-like family member 7	1.62	0.0129
PIN4	peptidylprolyl cis/trans isomerase, NIMA-interacting 4	1.62	0.00668
SRSF7	serine/arginine-rich splicing factor 7	1.60	0.0373
ZNF385D	zinc finger protein 385D	1.59	0.000949
DCAF17	DDB1 and CUL4 associated factor 17	1.58	0.00125
EIF4A3	eukaryotic translation initiation factor 4A3	1.58	0.0193
RPS13	ribosomal protein S13	1.58	0.0307
ZC3H12C	zinc finger CCCH-type containing 12C	1.57	0.00215
SLC50A1	solute carrier family 50 (sugar efflux transporter), member 1	1.55	0.00534
AHSA1	AHA1, activator of heat shock 90kDa protein ATPase homolog 1 (yeast)	1.55	0.0365
TEN1	TEN1 CST complex subunit	1.55	0.00264
KLHL15	kelch-like family member 15	1.54	0.00228
NCAM1	neural cell adhesion molecule 1	1.54	0.00441
ALG5	ALG5, dolichyl-phosphate beta-glucosyltransferase	1.54	0.0217
NDUFA5	NADH dehydrogenase (ubiquinone) 1 alpha subcomplex, 5	1.53	0.00837
MRPL3	mitochondrial ribosomal protein L3	1.53	0.0156
RRAD	Ras-related associated with diabetes	1.53	0.00202
DEPDC1	DEP domain containing 1	1.52	0.00215
SMDT1	single-pass membrane protein with aspartate-rich tail 1	1.52	0.00901
SDF2	stromal cell-derived factor 2	1.52	0.0188
NR4A2	nuclear receptor subfamily 4, group A, member 2	1.51	0.00759

BAIAP2	BAI1-associated protein 2	1.51	0.013
SLC1A3	solute carrier family 1 (glial high affinity glutamate transporter), member 3	1.51	0.0019
ERP29	endoplasmic reticulum protein 29	1.51	0.00239
PSMD9	proteasome 26S subunit, non-ATPase 9	1.51	0.0148
SOX5	SRY box 5	1.51	0.00191
THEM4	thioesterase superfamily member 4	1.51	0.0181
LARP7	La ribonucleoprotein domain family, member 7	1.51	0.0356
NQO1	NAD(P)H dehydrogenase, quinone 1	1.51	0.00473
ANKRD28	ankyrin repeat domain 28	1.50	0.0208
RSPH3	radial spoke 3 homolog (Chlamydomonas)	1.50	0.00968
STRADB	STE20-related kinase adaptor beta	1.50	0.0278
CERS2	ceramide synthase 2	1.50	0.0164
CLEC2D	C-type lectin domain family 2, member D	1.50	0.00198
MED15	mediator complex subunit 15	1.49	0.0408
TLCD1	TLC domain containing 1	1.49	0.00956
MRPL14	mitochondrial ribosomal protein L14	1.48	0.0233
PSMB5	proteasome subunit beta 5	1.48	0.025
SNRPE	small nuclear ribonucleoprotein polypeptide E	1.48	0.0444
MAP4K5	mitogen-activated protein kinase kinase kinase kinase 5	1.48	0.0197
NFIA	nuclear factor I/A	1.47	0.00413
ERI3	ERI1 exoribonuclease family member 3	1.47	0.0118
CTPS2	CTP synthase 2	1.47	0.00692
PPP6C	protein phosphatase 6, catalytic subunit	1.47	0.0383
PPP3CC	protein phosphatase 3, catalytic subunit, gamma isozyme	1.47	0.00471
CCNL1	cyclin L1	1.47	0.0207
TRAPPC2	trafficking protein particle complex 2	1.47	0.0103
MTIF2	mitochondrial translational initiation factor 2	1.47	0.0113
MRPL33	mitochondrial ribosomal protein L33	1.47	0.0187
AFF2	AF4/FMR2 family, member 2	1.46	0.00892
CYB5A	cytochrome b5 type A (microsomal)	1.46	0.0189
ZSWIM7	zinc finger, SWIM-type containing 7	1.46	0.0389
ZNF280B	zinc finger protein 280B	1.46	0.0175
SCML1	sex comb on midleg-like 1 (Drosophila)	1.46	0.0144
FAM200B	family with sequence similarity 200, member B	1.46	0.0152
ABCD3	ATP binding cassette subfamily D member 3	1.45	0.0155
PLPP1	phospholipid phosphatase 1	1.45	0.00896
TIGD5	tigger transposable element derived 5	1.45	0.0204
PRPF40A	PRP40 pre-mRNA processing factor 40 homolog A	1.45	0.0305
VPS33A	vacuolar protein sorting 33 homolog A (S. cerevisiae)	1.45	0.00916
TMEM126A	transmembrane protein 126A	1.45	0.0364
LUM	lumican	1.45	0.0349
OSGEP	O-sialoglycoprotein endopeptidase	1.44	0.0175
SNRPF	small nuclear ribonucleoprotein polypeptide F	1.44	0.0251
NDUFA4	NDUFA4, mitochondrial complex associated	1.44	0.0295
SETDB1	SET domain, bifurcated 1	1.44	0.0215
SOD1	superoxide dismutase 1, soluble	1.44	0.0188
SDHA	succinate dehydrogenase complex subunit A, flavoprotein (Fp)	1.43	0.023
MOXD1	monooxygenase, DBH-like 1	1.43	0.00694
ZCCHC7	zinc finger, CCHC domain containing 7	1.43	0.0104
KLHL2	kelch-like family member 2	1.43	0.0168
ANKHD1	ankyrin repeat and KH domain containing 1	1.43	0.0213
IGFBP7	insulin like growth factor binding protein 7	1.42	0.0248
TMED5	transmembrane p24 trafficking protein 5	1.42	0.00845
PPP1CC	protein phosphatase 1, catalytic subunit, gamma isozyme	1.42	0.0217
ORC5	origin recognition complex subunit 5	1.42	0.0145
MTHFS	5,10-methenyltetrahydrofolate synthetase (5-formyltetrahydrofolate cyclo-ligase)	1.42	0.0266
FBXW2	F-box and WD repeat domain containing 2	1.42	0.00852
ITPA	inosine triphosphatase (nucleoside triphosphate pyrophosphatase)	1.42	0.0257
TFPI	tissue factor pathway inhibitor (lipoprotein-associated coagulation inhibitor)	1.42	0.0102

SLC3A2	solute carrier family 3 (amino acid transporter heavy chain), member 2	1.41	0.0112
LATS1	large tumor suppressor kinase 1	1.41	0.0335
EPB41L5	erythrocyte membrane protein band 4.1 like 5	1.41	0.00772
ALDH1L2	aldehyde dehydrogenase 1 family, member L2	1.41	0.0498
YARS2	tyrosyl-tRNA synthetase 2, mitochondrial	1.41	0.0301
EIF3L	eukaryotic translation initiation factor 3, subunit L	1.41	0.0167
MCM6	minichromosome maintenance complex component 6	1.41	0.00692
KNDC1	Transcript Identified by AceView, Entrez Gene ID(s) 85442	1.41	0.0485
ZNF451	zinc finger protein 451	1.40	0.0448
FBXW7	F-box and WD repeat domain containing 7, E3 ubiquitin protein ligase	1.40	0.0123
NPLOC4	NPL4 homolog, ubiquitin recognition factor	1.40	0.0161
ZNF267	zinc finger protein 267	1.40	0.0149
HTATSF1	HIV-1 Tat specific factor 1	1.40	0.0135
ACSF2	acyl-CoA synthetase family member 2	1.40	0.0134
TTLL4	tubulin tyrosine ligase-like family member 4	1.40	0.0297
EIF2B3	eukaryotic translation initiation factor 2B, subunit 3 gamma, 58kDa	1.40	0.0163
RSRC1	arginine/serine-rich coiled-coil 1	1.40	0.0103
TSPAN4	tetraspanin 4	1.40	0.0106
RBM27	RNA binding motif protein 27	1.40	0.033
VPS26B	VPS26 retromer complex component B	1.40	0.0144
DDX56	DEAD (Asp-Glu-Ala-Asp) box helicase 56	1.40	0.0301
TACC1	transforming, acidic coiled-coil containing protein 1	1.39	0.0268
RPS19BP1	ribosomal protein S19 binding protein 1	1.39	0.00984
TYW3	tRNA-yW synthesizing protein 3 homolog (S. cerevisiae)	1.39	0.016
TECR	trans-2,3-enoyl-CoA reductase	1.39	0.0134
RNASET2	ribonuclease T2	1.39	0.012
TPD52L1	tumor protein D52-like 1	1.39	0.0242
LMO3	LIM domain only 3 (rhombotin-like 2)	1.39	0.0175
PRDX2	peroxiredoxin 2	1.38	0.0231
TBXAS1	thromboxane A synthase 1 (platelet)	1.38	0.0221
ZDHHC15	zinc finger, DHHC-type containing 15	1.38	0.0181
SAP130	Sin3A associated protein 130kDa	1.38	0.0458
POPDC3	popeye domain containing 3	1.38	0.0215
TMX3	thioredoxin-related transmembrane protein 3	1.38	0.0252
OGFOD3	2-oxoglutarate and iron-dependent oxygenase domain containing 3	1.38	0.0123
PFN2	profilin 2	1.38	0.0136
MGLL	monoglyceride lipase	1.38	0.0155
RAB31	RAB31, member RAS oncogene family	1.38	0.0377
ARL3	ADP-ribosylation factor like GTPase 3	1.38	0.0247
IFT27	intraflagellar transport 27	1.38	0.0109
LDAH	lipid droplet associated hydrolase	1.38	0.0196
RNF24	ring finger protein 24	1.38	0.0133
KIZ	kizuna centrosomal protein	1.37	0.0428
FXR1	fragile X mental retardation, autosomal homolog 1	1.37	0.0188
ANAPC16	anaphase promoting complex subunit 16	1.37	0.0272
LRRC27	leucine rich repeat containing 27	1.37	0.00979
TEX10	testis expressed 10	1.37	0.0293
CPEB1	cytoplasmic polyadenylation element binding protein 1	1.36	0.0179
POP4	POP4 homolog, ribonuclease P/MRP subunit	1.36	0.0402
FAXDC2	fatty acid hydroxylase domain containing 2	1.36	0.0131
TRMT10C	tRNA methyltransferase 10C, mitochondrial RNase P subunit	1.36	0.0192
ZNF101	zinc finger protein 101	1.36	0.0183
VPS52	vacuolar protein sorting 52 homolog (S. cerevisiae)	1.36	0.0186
GSTM3	glutathione S-transferase mu 3 (brain)	1.36	0.0167
RCN3	reticulocalbin 3, EF-hand calcium binding domain	1.36	0.0177
AOX1	aldehyde oxidase 1	1.36	0.0306
KCMF1	potassium channel modulatory factor 1	1.36	0.0144
PRKAG2	protein kinase, AMP-activated, gamma 2 non-catalytic subunit	1.36	0.0189
B3GAT2	beta-1,3-glucuronyltransferase 2	1.36	0.0294

MAPK6	mitogen-activated protein kinase 6	1.36	0.0231
INAFM2	InaF-motif containing 2	1.36	0.0384
INIP	INTS3 and NABP interacting protein	1.36	0.0226
SREBF2	sterol regulatory element binding transcription factor 2	1.35	0.0139
PPP4R1	protein phosphatase 4, regulatory subunit 1	1.35	0.0438
EIF2D	eukaryotic translation initiation factor 2D	1.35	0.0477
ISCA2	iron-sulfur cluster assembly 2	1.35	0.0391
FNIP2	folliculin interacting protein 2	1.35	0.0241
UROD	uroporphyrinogen decarboxylase	1.35	0.0477
LZIC	leucine zipper and CTNNBIP1 domain containing	1.35	0.0354
VWA5A	von Willebrand factor A domain containing 5A	1.35	0.036
TAF15	TATA box binding protein associated factor 15	1.35	0.0141
RPAIN	RPA interacting protein	1.35	0.0489
HSD17B4	hydroxysteroid (17-beta) dehydrogenase 4	1.35	0.0463
GSTT2	glutathione S-transferase theta 2 (gene/pseudogene)	1.35	0.0151
INPP5A	inositol polyphosphate-5-phosphatase A	1.35	0.0205
PRPSAP2	phosphoribosyl pyrophosphate synthetase-associated protein 2	1.35	0.046
KLHL3	kelch-like family member 3	1.35	0.0329
NPRL2	NPR2-like, GATOR1 complex subunit	1.35	0.0166
JUN	jun proto-oncogene	1.34	0.0265
CCDC12	coiled-coil domain containing 12	1.34	0.0261
CXCL14	chemokine (C-X-C motif) ligand 14	1.34	0.0205
DUSP10	dual specificity phosphatase 10	1.34	0.0151
ZNF366	zinc finger protein 366	1.34	0.0172
PSTK	phosphoseryl-tRNA kinase	1.34	0.0193
ERGIC2	ERGIC and golgi 2	1.34	0.0248
RNF7	ring finger protein 7	1.34	0.0443
MAP3K12	mitogen-activated protein kinase kinase kinase 12	1.34	0.0201
S100A7A	S100 calcium binding protein A7A	1.34	0.0335
TMEM97	transmembrane protein 97	1.34	0.0284
YME1L1	YME1-like 1 ATPase	1.34	0.0339
EGR3	early growth response 3	1.34	0.0225
KIAA1328	KIAA1328	1.34	0.0288
PFDN4	prefoldin subunit 4	1.34	0.0476
CCNDBP1	cyclin D-type binding-protein 1	1.34	0.0239
STEAP1	six transmembrane epithelial antigen of the prostate 1	1.34	0.0387
PREB	prolactin regulatory element binding	1.34	0.0245
CDK10	cyclin-dependent kinase 10	1.34	0.0368
PSMD8	proteasome 26S subunit, non-ATPase 8	1.33	0.0229
LRRC69	leucine rich repeat containing 69	1.33	0.0443
GNAI1	guanine nucleotide binding protein (G protein), alpha inhibiting activity polypeptide	1.33	0.0264
EMC4	ER membrane protein complex subunit 4	1.33	0.0468
CHCHD4	coiled-coil-helix-coiled-coil-helix domain containing 4	1.33	0.0259
VAV3	vav 3 guanine nucleotide exchange factor	1.33	0.0238
STPG1	sperm-tail PG-rich repeat containing 1	1.33	0.0309
FAM50A	family with sequence similarity 50, member A	1.33	0.0381
GTPBP10	GTP-binding protein 10 (putative)	1.33	0.0375
PDE3A	phosphodiesterase 3A, cGMP-inhibited	1.33	0.0346
CMTM4	CKLF-like MARVEL transmembrane domain containing 4	1.33	0.0201
BNC1	basonuclin 1	1.33	0.0208
MPEG1	macrophage expressed 1	1.33	0.0396
OSCP1	organic solute carrier partner 1	1.33	0.0253
FBXO27	F-box protein 27	1.33	0.0353
JMJD1C	jumonji domain containing 1C	1.33	0.0223
ZNF583	zinc finger protein 583	1.33	0.0336
KIF3B	kinesin family member 3B	1.33	0.042
RPEL1	ribulose-5-phosphate-3-epimerase-like 1	1.33	0.0288
KDM2B	lysine (K)-specific demethylase 2B	1.33	0.0462
ATAD2B	ATPase family, AAA domain containing 2B	1.33	0.0394

TANGO6	transport and golgi organization 6 homolog	1.33	0.0227
COMMD3-	COMMD3-BMI1 readthrough	1.33	0.0322
PDE8A	phosphodiesterase 8A	1.33	0.0422
RBBP6	retinoblastoma binding protein 6	1.33	0.0412
EXD2	exonuclease 3-5 domain containing 2	1.33	0.0212
URM1	ubiquitin related modifier 1	1.33	0.0444
NSF	N-ethylmaleimide-sensitive factor	1.33	0.0476
OLFM4	olfactomedin 4	1.33	0.0338
TMEM184C	transmembrane protein 184C	1.33	0.0478
GPBP1	GC-rich promoter binding protein 1	1.32	0.0429
STEAP1B	STEAP family member 1B	1.32	0.0369
RNF170	ring finger protein 170	1.32	0.0241
COQ7	coenzyme Q7 homolog, ubiquinone (yeast)	1.32	0.0321
ELOVL6	ELOVL fatty acid elongase 6	1.32	0.0372
ARL15	ADP-ribosylation factor like GTPase 15	1.32	0.0223
PRRX1	paired related homeobox 1	1.32	0.0481
LMAN2	lectin, mannose-binding 2	1.32	0.0421
SEMA6C	sema domain, transmembrane domain (TM), and cytoplasmic domain, (semaphorin)	1.32	0.049
UNC119B	unc-119 lipid binding chaperone B	1.32	0.0435
HK1	hexokinase 1	1.32	0.028
NRP1	neuropilin 1	1.31	0.0484
FAM3D	family with sequence similarity 3, member D	1.31	0.0231
C11orf68	chromosome 11 open reading frame 68	1.31	0.0262
PSMB4	proteasome subunit beta 4	1.31	0.0491
ADI1	acireductone dioxygenase 1	1.31	0.0418
TMEM185B	transmembrane protein 185B	1.31	0.0275
SH3BGRL2	SH3 domain binding glutamate-rich protein like 2	1.31	0.047
RNASE13	ribonuclease, RNase A family, 13 (non-active)	1.31	0.023
SIN3A	SIN3 transcription regulator family member A	1.31	0.0263
MSRA	methionine sulfoxide reductase A	1.31	0.0414
SLC24A1	solute carrier family 24 (sodium/potassium/calcium exchanger), member 1	1.31	0.0326
BTBD3	BTB (POZ) domain containing 3	1.31	0.035
PPP4R3B	protein phosphatase 4, regulatory subunit 3B	1.31	0.0344
HDHD2	haloacid dehalogenase-like hydrolase domain containing 2	1.31	0.0306
ZIC5	Zic family member 5	1.31	0.0292
CCDC151	coiled-coil domain containing 151	1.31	0.0479
ICE1	interactor of little elongation complex ELL subunit 1	1.30	0.043
F2RL2	coagulation factor II (thrombin) receptor-like 2	1.30	0.0346
SART3	squamous cell carcinoma antigen recognized by T-cells 3	1.30	0.0464
GPRC5A	G protein-coupled receptor, class C, group 5, member A	1.30	0.0426
CES3	carboxylesterase 3	1.30	0.0367
RAB8A	RAB8A, member RAS oncogene family	1.30	0.0487
UBIAD1	UbiA prenyltransferase domain containing 1	1.30	0.0361
TOMM40L	translocase of outer mitochondrial membrane 40 homolog (yeast)-like	1.30	0.046
ARL6IP5	ADP-ribosylation factor like GTPase 6 interacting protein 5	1.30	0.0359
GFM1	G elongation factor, mitochondrial 1	1.30	0.0477
NETO1	neuropilin (NRP) and tolloid (TLL)-like 1	1.30	0.0451
FMO5	flavin containing monooxygenase 5	1.30	0.0314
PITPNC1	phosphatidylinositol transfer protein, cytoplasmic 1	1.29	0.0389
APIP	APAF1 interacting protein	1.29	0.0424
HSPBP1	HSPA (heat shock 70kDa) binding protein, cytoplasmic cochaperone 1	1.29	0.0304
SGCZ	sarcoglycan zeta	1.29	0.0451
GMPR	guanosine monophosphate reductase	1.29	0.0404
GUF1	GUF1 homolog, GTPase	1.29	0.0482
COG6	component of oligomeric golgi complex 6	1.29	0.048
HDAC5	histone deacetylase 5	1.29	0.0387
STARD3	StAR-related lipid transfer domain containing 3	1.29	0.035
GPR15	G protein-coupled receptor 15	1.29	0.034
MICA	MHC class I polypeptide-related sequence A	1.29	0.0407

SLC25A15	solute carrier family 25 (mitochondrial carrier; ornithine transporter) member 15	1.29	0.0475
PARD3	par-3 family cell polarity regulator	1.29	0.0426
ZFP64	ZFP64 zinc finger protein	1.29	0.0399
FRG2B	FSHD region gene 2 family, member B	1.28	0.0488
OXSM	3-oxoacyl-ACP synthase, mitochondrial	1.28	0.0401
C12orf76	chromosome 12 open reading frame 76	1.28	0.0383
MOV10	Mov10 RISC complex RNA helicase	1.28	0.0385
GLT8D2	glycosyltransferase 8 domain containing 2	1.28	0.0484
BMP6	bone morphogenetic protein 6	1.28	0.0373
ARMCX2	armadillo repeat containing, X-linked 2	1.28	0.05
EPHA1	EPH receptor A1	1.27	0.0484
TOX	thymocyte selection-associated high mobility group box	1.27	0.0436
ZFP82	ZFP82 zinc finger protein	1.27	0.0477
TRIM24	tripartite motif containing 24	1.27	0.0484
NEB	nebulin	1.27	0.0472
KIF20A	kinesin family member 20A	1.26	0.0497
FAM111A	family with sequence similarity 111, member A	1.26	0.0472

Table S2. MMF-decreased genes (P < 0.05 with FC < 0.80). The table lists 142 MMF-decreased genes ranked by estimated fold-change (FC) (MMF/CTL). The gene symbol and description are shown. The raw p-value is listed in the final column (generalized least square linear models with moderated t-statistics; *n* = 3 samples per group).

Symbol	Description	FC	P-value
TP53INP1	tumor protein p53 inducible nuclear protein 1	0.57	0.00853
TCEA1	transcription elongation factor A (SII), 1	0.64	0.0166
F3	coagulation factor III (thromboplastin, tissue factor)	0.65	0.00141
ZC3H4	zinc finger CCCH-type containing 4	0.66	0.00186
SLC20A2	solute carrier family 20 (phosphate transporter), member 2	0.67	0.00457
ATP6V1E2	ATPase, H+ transporting, lysosomal 31kDa, V1 subunit E2	0.67	0.00524
OSBPL10	oxysterol binding protein-like 10	0.68	0.00243
CCDC81	coiled-coil domain containing 81	0.68	0.0051
IRAK4	interleukin 1 receptor associated kinase 4	0.68	0.0231
TSSC4	tumor suppressing subtransferable candidate 4	0.69	0.00458
SFXN1	sideroflexin 1	0.69	0.0064
PIGU	Salzman2013 ALT_ACCEPTOR, ALT_DONOR, coding, INTERNAL,	0.69	0.00843
GPR157	G protein-coupled receptor 157	0.69	0.00439
TOP2A	topoisomerase (DNA) II alpha	0.70	0.0084
GLIS2	GLIS family zinc finger 2	0.70	0.00901
KLHL18	kelch-like family member 18	0.70	0.00659
PHLPP1	PH domain and leucine rich repeat protein phosphatase 1	0.70	0.00874
PGM3	phosphoglucomutase 3	0.70	0.0189
YPEL2	yippee like 2	0.71	0.0241
CCNF	cyclin F	0.71	0.00704
PRR5L	proline rich 5 like	0.71	0.0154
CTSL	cathepsin L	0.71	0.0139
KLHL20	kelch-like family member 20	0.71	0.0108
ATP6V1C2	ATPase, H+ transporting, lysosomal 42kDa, V1 subunit C2	0.71	0.0171
TGDS	TDP-glucose 4,6-dehydratase	0.72	0.00897
IMPACT	impact RWD domain protein	0.72	0.0114
DDR2	discoidin domain receptor tyrosine kinase 2	0.72	0.0085
FES	FES proto-oncogene, tyrosine kinase	0.72	0.028
FCER1G	Fc fragment of IgE, high affinity I, receptor for; gamma polypeptide	0.72	0.0195
SMARCD1	SWI/SNF related, matrix associated, actin dependent regulator of chromatin,	0.72	0.0402
NUTM2G	NUT family member 2G	0.73	0.0258
UBE2W	ubiquitin-conjugating enzyme E2W (putative)	0.73	0.0236
CDKN1B	cyclin-dependent kinase inhibitor 1B (p27, Kip1)	0.73	0.0196
GRIN2C	glutamate receptor, ionotropic, N-methyl D-aspartate 2C	0.73	0.0201
SPTLC1	serine palmitoyltransferase, long chain base subunit 1	0.73	0.0264
KRTAP2-1	keratin associated protein 2-1	0.73	0.0129
C11orf96	chromosome 11 open reading frame 96	0.73	0.0122
ZNF616	zinc finger protein 616	0.73	0.0492
EIF2S3	eukaryotic translation initiation factor 2, subunit 3 gamma, 52kDa	0.73	0.0324
ABHD14A	abhydrolase domain containing 14A	0.73	0.0412
PRSS27	protease, serine 27	0.73	0.0157
ARFGEF2	ADP-ribosylation factor guanine nucleotide-exchange factor 2 (brefeldin A-	0.73	0.0241
RASL10A	RAS-like, family 10, member A	0.74	0.0124
UBR5	ubiquitin protein ligase E3 component n-recognin 5	0.74	0.039
MIB1	mindbomb E3 ubiquitin protein ligase 1	0.74	0.0283
C8G	complement component 8, gamma polypeptide	0.74	0.0151
FAM114A2	family with sequence similarity 114, member A2	0.74	0.0239
SNX11	sorting nexin 11	0.74	0.0273
RALGPS1	Ral GEF with PH domain and SH3 binding motif 1	0.74	0.0145
MTMR2	myotubularin related protein 2	0.74	0.0165
C1QTNF3	C1q and tumor necrosis factor related protein 3	0.74	0.0154
ENHO	energy homeostasis associated	0.74	0.023
PTPRJ	protein tyrosine phosphatase, receptor type, J	0.74	0.0317
LRFN3	leucine rich repeat and fibronectin type III domain containing 3	0.74	0.0186
NUAK1	NUAK family, SNF1-like kinase, 1	0.74	0.026

ZNF548	zinc finger protein 548	0.74	0.0149
MAPK8IP2	mitogen-activated protein kinase 8 interacting protein 2	0.75	0.0174
NKX2-1	NK2 homeobox 1	0.75	0.0242
SIM2	single-minded family bHLH transcription factor 2	0.75	0.0457
CAND2	cullin-associated and neddylation-dissociated 2 (putative)	0.75	0.0166
TBX15	T-box 15	0.75	0.0359
KRTAP2-2	keratin associated protein 2-2	0.75	0.018
ACSL4	acyl-CoA synthetase long-chain family member 4	0.75	0.0239
NOX4	NADPH oxidase 4	0.75	0.0422
KDM8	lysine (K)-specific demethylase 8	0.75	0.0239
LUZP1	leucine zipper protein 1	0.75	0.0268
CDC23	cell division cycle 23	0.75	0.0371
USP25	ubiquitin specific peptidase 25	0.75	0.0394
ANKRD61	ankyrin repeat domain 61	0.75	0.0345
WWC1	WW and C2 domain containing 1	0.76	0.0266
SACM1L	SAC1 suppressor of actin mutations 1-like (yeast)	0.76	0.0284
PLEC	plectin	0.76	0.0369
ANKLE2	ankyrin repeat and LEM domain containing 2	0.76	0.0311
FADS6	fatty acid desaturase 6	0.76	0.0456
CEMIP	cell migration inducing protein, hyaluronan binding	0.76	0.0232
ZNF250	zinc finger protein 250	0.76	0.026
TSPAN13	tetraspanin 13	0.76	0.0448
TMEM177	transmembrane protein 177	0.76	0.0313
XAGE5	X antigen family, member 5	0.76	0.0287
BPIFB6	BPI fold containing family B, member 6	0.76	0.0347
DMWD	dystrophia myotonica, WD repeat containing	0.76	0.0314
CACNA1G	calcium channel, voltage-dependent, T type, alpha 1G subunit	0.76	0.0411
RCAN1	regulator of calcineurin 1	0.76	0.0479
USP21	ubiquitin specific peptidase 21	0.76	0.0483
CYP27A1	cytochrome P450, family 27, subfamily A, polypeptide 1	0.76	0.0341
TNPO1	transportin 1	0.76	0.0244
SMCR8	Smith-Magenis syndrome chromosome region, candidate 8	0.76	0.0324
PELI2	pellino E3 ubiquitin protein ligase family member 2	0.76	0.0367
YAP1	Yes-associated protein 1	0.76	0.0388
DNAJC9	DnaJ (Hsp40) homolog, subfamily C, member 9	0.76	0.0369
SDHB	succinate dehydrogenase complex subunit B, iron sulfur (Ip)	0.76	0.0366
CFAP73	cilia and flagella associated protein 73	0.76	0.0315
MRS2	MRS2 magnesium transporter	0.76	0.0355
SP5	Sp5 transcription factor	0.77	0.0303
METTL15	methyltransferase like 15	0.77	0.0499
GPT	glutamic-pyruvate transaminase (alanine aminotransferase)	0.77	0.0301
CCDC190	coiled-coil domain containing 190	0.77	0.0413
ATP6AP1L	ATPase, H+ transporting, lysosomal accessory protein 1-like	0.77	0.0366
ST6GAL2	ST6 beta-galactosamide alpha-2,6-sialyltranferase 2	0.77	0.0467
FLOT2	Memczak2013 ANTISENSE, coding, INTERNAL, UTR3 best transcript	0.77	0.0303
PARPBP	PARP1 binding protein	0.77	0.0477
RGL3	ral guanine nucleotide dissociation stimulator-like 3	0.77	0.0424
FSTL3	follistatin-like 3 (secreted glycoprotein)	0.77	0.0373
TBC1D3	TBC1 domain family, member 3	0.77	0.0361
SSRP1	structure specific recognition protein 1	0.77	0.0351
KCNH1	potassium channel, voltage gated eag related subfamily H, member 1	0.77	0.0488
AHDC1	AT hook, DNA binding motif, containing 1	0.77	0.0314
CEACAM19	carcinoembryonic antigen-related cell adhesion molecule 19	0.77	0.0473
ZNF816	zinc finger protein 816	0.77	0.0369
DOLPP1	dolichyldiphosphatase 1	0.77	0.0336
SLC44A5	solute carrier family 44, member 5	0.77	0.035
CHST14	carbohydrate (N-acetylgalactosamine 4-0) sulfotransferase 14	0.77	0.036
UPF2	UPF2 regulator of nonsense transcripts homolog (yeast)	0.77	0.03
CREB3L3	cAMP responsive element binding protein 3-like 3	0.77	0.0331

SLC26A11	solute carrier family 26 (anion exchanger), member 11	0.77	0.037
HIBCH	Transcript Identified by AceView, Entrez Gene ID(s) 26275	0.77	0.0499
ERCC6	excision repair cross-complementation group 6	0.77	0.0449
ATF7IP	activating transcription factor 7 interacting protein	0.77	0.0454
STK11IP	serine/threonine kinase 11 interacting protein	0.77	0.0412
ARHGAP22	Rho GTPase activating protein 22	0.77	0.0318
MFSD14A	major facilitator superfamily domain containing 14A	0.78	0.0428
GPR4	G protein-coupled receptor 4	0.78	0.0463
SETD9	SET domain containing 9	0.78	0.0396
NMB	neuromedin B	0.78	0.0401
REPS1	RALBP1 associated Eps domain containing 1	0.78	0.0373
ERCC5	excision repair cross-complementation group 5	0.78	0.0424
C12orf50	chromosome 12 open reading frame 50	0.78	0.0392
PLEKHG3	pleckstrin homology domain containing, family G (with RhoGef domain)	0.78	0.0372
LTB	lymphotoxin beta (TNF superfamily, member 3)	0.78	0.0488
DTWD2	DTW domain containing 2	0.78	0.0466
ZNF439	zinc finger protein 439	0.78	0.0395
GPRIN1	G protein regulated inducer of neurite outgrowth 1	0.78	0.0472
CD82	CD82 molecule	0.78	0.0398
LYN	LYN proto-oncogene, Src family tyrosine kinase	0.78	0.0467
MAP6D1	MAP6 domain containing 1	0.78	0.0474
MPZ	myelin protein zero	0.79	0.046
DCLK2	doublecortin-like kinase 2	0.79	0.0435
NDST2	N-deacetylase/N-sulfotransferase (heparan glucosaminyl) 2	0.79	0.0444
TRNP1	TMF1-regulated nuclear protein 1	0.79	0.0466
MC2R	melanocortin 2 receptor (adrenocorticotropic hormone)	0.79	0.0433
PTP4A2	protein tyrosine phosphatase type IVA, member 2	0.79	0.0439
EMID1	EMI domain containing 1	0.79	0.0484

Table S3. DRF-increased genes (P < 0.05 with FC > 1.25). The table lists 181 DRF-increased genes ranked by estimated fold-change (FC) (DRF/CTL). The gene symbol and description are shown. The raw p-value is listed in the final column (generalized least square linear models with moderated t-statistics; *n* = 3 samples per group).

Symbol	Description	FC	P-value
TIGD5	tigger transposable element derived 5	1.64	0.00427
ABI3BP	ABI family, member 3 (NESH) binding protein	1.64	0.00234
SPATA25	spermatogenesis associated 25	1.60	0.00194
NEFM	neurofilament, medium polypeptide	1.59	0.00353
ZDHHC15	zinc finger, DHHC-type containing 15	1.57	0.0083
NDUFAF6	NADH dehydrogenase (ubiquinone) complex I, assembly factor 6	1.56	0.0141
FAM83D	family with sequence similarity 83, member D	1.53	0.00922
ACSL6	acyl-CoA synthetase long-chain family member 6	1.52	0.00427
TPPP3	tubulin polymerization-promoting protein family member 3	1.49	0.0131
ZNF180	zinc finger protein 180	1.49	0.00439
SCN2A	sodium channel, voltage gated, type II alpha subunit	1.48	0.0384
PSIP1	PC4 and SFRS1 interacting protein 1	1.48	0.00735
SLITRK5	SLIT and NTRK-like family, member 5	1.47	0.00546
TXNRD1	thioredoxin reductase 1	1.47	0.00951
GHRHR	growth hormone releasing hormone receptor	1.46	0.0101
GSAP	gamma-secretase activating protein	1.45	0.00431
CD33	CD33 molecule	1.45	0.00473
ALDH1L1	aldehyde dehydrogenase 1 family, member L1	1.45	0.00857
HLA-DRB5	major histocompatibility complex, class II, DR beta 5	1.45	0.00685
CLRN2	clarin 2	1.45	0.00786
SIX3	SIX homeobox 3	1.44	0.00659
SLC50A1	solute carrier family 50 (sugar efflux transporter), member 1	1.44	0.0101
SLA	Src-like-adaptor	1.44	0.0129
BCDIN3D	BCDIN3 domain containing	1.43	0.0407
RPEL1	ribulose-5-phosphate-3-epimerase-like 1	1.43	0.0125
OR2L3	olfactory receptor, family 2, subfamily L, member 3	1.43	0.0155
ULK3	unc-51 like kinase 3	1.43	0.0165
SCPEP1	serine carboxypeptidase 1	1.43	0.00789
FAM216B	family with sequence similarity 216, member B	1.43	0.00999
GUF1	GUF1 homolog, GTPase	1.43	0.0159
WDR4	WD repeat domain 4	1.42	0.00767
RAP2C	RAP2C, member of RAS oncogene family	1.42	0.0217
FAT2	FAT atypical cadherin 2	1.42	0.0171
GJB7	gap junction protein beta 7	1.42	0.0074
ATF3	activating transcription factor 3	1.42	0.00823
FBXO27	F-box protein 27	1.42	0.0129
METTL25	methyltransferase like 25	1.42	0.026
GP9	glycoprotein IX (platelet)	1.41	0.014
CST9	cystatin 9 (testatin)	1.41	0.0166
AANAT	aralkylamine N-acetyltransferase	1.41	0.00922
OSGIN1	oxidative stress induced growth inhibitor 1	1.41	0.0105
TACC2	transforming, acidic coiled-coil containing protein 2	1.41	0.0203
MCEMP1	mast cell-expressed membrane protein 1	1.41	0.0337
RNF40	ring finger protein 40, E3 ubiquitin protein ligase	1.40	0.0125
STX3	syntaxin 3	1.40	0.0278
PRDM1	PR domain containing 1, with ZNF domain	1.40	0.0103
OR4F17	olfactory receptor, family 4, subfamily F, member 17	1.40	0.0107
RAD18	RAD18 E3 ubiquitin protein ligase	1.40	0.0392
GPAT2	glycerol-3-phosphate acyltransferase 2, mitochondrial	1.40	0.0142
C2orf68	chromosome 2 open reading frame 68	1.40	0.0287
B3GNT4	UDP-GlcNAc:betaGal beta-1,3-N-acetylglucosaminyltransferase 4	1.40	0.0125
FOSB	FBJ murine osteosarcoma viral oncogene homolog B	1.40	0.0194
SHISA6	shisa family member 6	1.40	0.0141
TBXAS1	thromboxane A synthase 1 (platelet)	1.39	0.0189
INAFM1	InaF-motif containing 1	1.39	0.0143

NQO1	NAD(P)H dehydrogenase, quinone 1	1.39	0.0247
G6PD	glucose-6-phosphate dehydrogenase	1.39	0.015
OPRL1	opiate receptor-like 1	1.39	0.0226
PIK3CG	Memczak2013 ANTISENSE, CDS, coding, INTERNAL best transcript	1.39	0.0256
LRFN5	leucine rich repeat and fibronectin type III domain containing 5	1.39	0.0172
PRDM15	PR domain containing 15	1.38	0.0229
TBX2	T-box 2	1.38	0.0142
BMP10	bone morphogenetic protein 10	1.38	0.0279
CENPP	centromere protein P	1.38	0.0227
OR2Z1	olfactory receptor, family 2, subfamily Z, member 1	1.38	0.0465
FAM221A	family with sequence similarity 221, member A	1.38	0.039
UNC93B1	unc-93 homolog B1 (C. elegans)	1.38	0.0233
BCKDHB	branched chain keto acid dehydrogenase E1, beta polypeptide	1.38	0.034
GPR153	G protein-coupled receptor 153	1.38	0.013
BLOC1S3	biogenesis of lysosomal organelles complex-1, subunit 3	1.38	0.0225
SEMA4D	Memczak2013 ANTISENSE, coding, INTERNAL, UTR3 best transcript	1.37	0.033
SLC12A5	solute carrier family 12 (potassium/chloride transporter), member 5	1.37	0.0205
HS3ST2	heparan sulfate (glucosamine) 3-O-sulfotransferase 2	1.37	0.0209
CELA2B	chymotrypsin-like elastase family, member 2B	1.37	0.0243
FOXO6	forkhead box O6	1.37	0.0187
TTLL4	tubulin tyrosine ligase-like family member 4	1.37	0.037
TACC1	transforming, acidic coiled-coil containing protein 1	1.37	0.0417
RAPGEF3	Rap guanine nucleotide exchange factor 3	1.37	0.021
RCBTB1	regulator of chromosome condensation (RCC1) and BTB (POZ) domain	1.37	0.0197
STK4	serine/threonine kinase 4	1.37	0.016
GIPC2	GIPC PDZ domain containing family, member 2	1.37	0.0321
IFT81	intraflagellar transport 81	1.36	0.0234
ZNF366	zinc finger protein 366	1.36	0.0183
NFATC2	nuclear factor of activated T-cells, cytoplasmic, calcineurin-dependent 2	1.36	0.0179
PAPSS2	3-phosphoadenosine 5-phosphosulfate synthase 2	1.36	0.0206
MGST1	microsomal glutathione S-transferase 1	1.36	0.0266
MAN2A2	mannosidase, alpha, class 2A, member 2	1.35	0.0401
CHRNB2	cholinergic receptor, nicotinic beta 2	1.35	0.024
SOX30	SRY box 30	1.35	0.0293
UBL4A	ubiquitin-like 4A	1.35	0.02
LY6G6C	lymphocyte antigen 6 complex, locus G6C	1.35	0.0286
GSR	glutathione reductase	1.35	0.0171
PLAC8L1	PLAC8-like 1	1.35	0.036
ZNF341	zinc finger protein 341	1.35	0.0238
CPXM2	carboxypeptidase X (M14 family), member 2	1.35	0.0194
RHD	Rh blood group, D antigen	1.35	0.0202
GRK6	G protein-coupled receptor kinase 6	1.35	0.0221
DUOXA2	dual oxidase maturation factor 2	1.35	0.0353
NANOG	Nanog homeobox	1.35	0.0182
FAM71A	family with sequence similarity 71, member A	1.35	0.0246
DAGLB	diacylglycerol lipase, beta	1.35	0.0493
EGFR	epidermal growth factor receptor	1.35	0.0403
HCAR3	hydroxycarboxylic acid receptor 3	1.35	0.0219
RNF113A	ring finger protein 113A	1.35	0.0223
FRG2	FSHD region gene 2	1.35	0.0441
SCO1	SCO1 cytochrome c oxidase assembly protein	1.35	0.02
P2RX2	purinergic receptor P2X, ligand gated ion channel, 2	1.35	0.027
PAQR6	progestin and adipoQ receptor family member VI	1.35	0.0382
CNR2	cannabinoid receptor 2	1.35	0.0199
RNF111	ring finger protein 111	1.35	0.0297
PRG3	proteoglycan 3	1.34	0.025
WDFY3	WD repeat and FYVE domain containing 3	1.34	0.0249
ZACN	zinc activated ligand-gated ion channel	1.34	0.0269
NFIA	nuclear factor I/A	1.34	0.0371

TRIM6	tripartite motif containing 6	1.34	0.0469
RPUSD4	RNA pseudouridylate synthase domain containing 4	1.34	0.0424
RNF170	ring finger protein 170	1.34	0.0218
PBRM1	polybromo 1	1.34	0.0331
NLRP12	NLR family, pyrin domain containing 12	1.34	0.0219
CFAP46	cilia and flagella associated protein 46	1.34	0.0242
EPHA8	EPH receptor A8	1.33	0.0382
GCLC	glutamate-cysteine ligase, catalytic subunit	1.33	0.0369
IKZF4	IKAROS family zinc finger 4	1.33	0.029
SYNGR4	synaptogyrin 4	1.33	0.033
CAPN3	calpain 3	1.33	0.0396
GMPR	guanosine monophosphate reductase	1.33	0.0294
EID3	EP300 interacting inhibitor of differentiation 3	1.33	0.0298
CYP11B2	cytochrome P450, family 11, subfamily B, polypeptide 2	1.33	0.0263
ZNF385D	zinc finger protein 385D	1.33	0.0445
APIP	APAF1 interacting protein	1.33	0.0349
CDC42EP4	CDC42 effector protein (Rho GTPase binding) 4	1.33	0.0266
TRIM73	tripartite motif containing 73	1.33	0.0363
PTGR1	prostaglandin reductase 1	1.32	0.04
MYOZ3	myozenin 3	1.32	0.045
TMEM97	transmembrane protein 97	1.32	0.0379
GPR6	G protein-coupled receptor 6	1.32	0.0374
SLC22A8	solute carrier family 22 (organic anion transporter), member 8	1.32	0.0417
PCID2	PCI domain containing 2	1.32	0.0404
CACNA1H	calcium channel, voltage-dependent, T type, alpha 1H subunit	1.32	0.047
GMPR2	guanosine monophosphate reductase 2	1.32	0.0313
PFKM	phosphofructokinase, muscle	1.32	0.035
IL2RG	interleukin 2 receptor, gamma	1.32	0.0392
PIK3R5	phosphoinositide-3-kinase, regulatory subunit 5	1.32	0.0298
FMO5	flavin containing monooxygenase 5	1.32	0.0279
FBXO34	F-box protein 34	1.32	0.0382
TRIOBP	TRIO and F-actin binding protein	1.31	0.0459
PPARA	peroxisome proliferator-activated receptor alpha	1.31	0.0361
C2orf83	chromosome 2 open reading frame 83	1.31	0.041
GSKIP	GSK3B interacting protein	1.31	0.0378
ACTRT3	actin-related protein T3	1.31	0.0449
MYLK4	myosin light chain kinase family member 4	1.31	0.0374
TRIM49D1	tripartite motif containing 49D1	1.31	0.0379
NR1D1	nuclear receptor subfamily 1, group D, member 1	1.31	0.0468
C10orf95	chromosome 10 open reading frame 95	1.31	0.0479
TAS2R10	taste receptor, type 2, member 10	1.31	0.0409
PRAMEF9	---	1.31	0.0425
LCE5A	late cornified envelope 5A	1.31	0.0411
ARL4D	ADP-ribosylation factor like GTPase 4D	1.31	0.0435
ELAC1	elaC ribonuclease Z 1	1.31	0.0468
OR52M1	olfactory receptor, family 52, subfamily M, member 1	1.30	0.0422
TP53INP2	tumor protein p53 inducible nuclear protein 2	1.30	0.0459
ISX	intestine-specific homeobox	1.30	0.0426
AOX1	aldehyde oxidase 1	1.30	0.0414
FETUB	fetuin B	1.30	0.0345
FGF6	fibroblast growth factor 6	1.30	0.0384
SVEP1	sushi, von Willebrand factor type A, EGF and pentraxin domain containing 1	1.30	0.0383
GPR161	G protein-coupled receptor 161	1.30	0.04
AGPAT5	1-acylglycerol-3-phosphate O-acyltransferase 5	1.30	0.0385
C17orf97	chromosome 17 open reading frame 97	1.30	0.0362
ALDH3A2	aldehyde dehydrogenase 3 family, member A2	1.30	0.0389
KIF25	kinesin family member 25	1.30	0.0495
ST3GAL5	ST3 beta-galactoside alpha-2,3-sialyltransferase 5	1.30	0.0441
BEST1	bestrophin 1	1.30	0.0386

BCAS1	breast carcinoma amplified sequence 1	1.29	0.0385
PAFAH2	platelet-activating factor acetylhydrolase 2	1.29	0.0449
CRYGA	crystallin gamma A	1.29	0.0461
ROM1	retinal outer segment membrane protein 1	1.29	0.0468
APOBEC2	apolipoprotein B mRNA editing enzyme, catalytic polypeptide-like 2	1.29	0.0416
SLC1A3	solute carrier family 1 (glial high affinity glutamate transporter), member 3	1.28	0.0497
LIMD1	LIM domains containing 1	1.28	0.0499
RASSF8	Ras association (RalGDS/AF-6) domain family (N-terminal) member 8	1.28	0.0494

Table S4. DRF-decreased genes (P < 0.05 with FC < 0.80). The table lists 198 DRF-decreased genes ranked by estimated fold-change (FC) (DRF/CTL). The gene symbol and description are shown. The raw p-value is listed in the final column (generalized least square linear models with moderated t-statistics; *n* = 3 samples per group).

Symbol	Description	FC	P-value
ACTR3	ARP3 actin-related protein 3 homolog (yeast)	0.52	0.00245
SACM1L	SAC1 suppressor of actin mutations 1-like (yeast)	0.53	0.0000774
NOX4	NADPH oxidase 4	0.53	0.000157
CLK1	CDC like kinase 1	0.54	0.0121
RAP1B	RAP1B, member of RAS oncogene family	0.54	0.00318
TIMM9	translocase of inner mitochondrial membrane 9 homolog (yeast)	0.56	0.00304
MOCS2	molybdenum cofactor synthesis 2	0.57	0.00457
PRKCI	protein kinase C, iota	0.58	0.0255
FCHSD2	FCH and double SH3 domains 2	0.58	0.00106
KLHDC2	kelch domain containing 2	0.58	0.0179
TAF9	TAF9 RNA polymerase II, TATA box binding protein (TBP)-associated	0.59	0.00255
REXO2	RNA exonuclease 2	0.59	0.00941
WDR41	WD repeat domain 41	0.59	0.0369
LYPLAL1	lysophospholipase-like 1	0.59	0.0174
UCHL3	ubiquitin C-terminal hydrolase L3	0.60	0.0398
LNPEP	leucyl/cystinyl aminopeptidase	0.61	0.0193
NAE1	NEDD8 activating enzyme E1 subunit 1	0.61	0.00129
CCT2	chaperonin containing TCP1, subunit 2 (beta)	0.61	0.00926
LSM1	LSM1 homolog, mRNA degradation associated	0.61	0.0284
TANK	TRAF family member-associated NFKB activator	0.63	0.0486
RIOK1	RIO kinase 1	0.63	0.0216
ZNF24	zinc finger protein 24	0.64	0.00344
HACD3	3-hydroxyacyl-CoA dehydratase 3	0.64	0.0218
SFT2D1	SFT2 domain containing 1	0.64	0.0359
C20orf194	chromosome 20 open reading frame 194	0.64	0.0176
UBR5	ubiquitin protein ligase E3 component n-recognin 5	0.64	0.0104
ZZZ3	zinc finger, ZZ-type containing 3	0.64	0.018
RAP1A	RAP1A, member of RAS oncogene family	0.64	0.00542
NUP50	nucleoporin 50kDa	0.64	0.0252
RAD50	RAD50 homolog, double strand break repair protein	0.64	0.00656
RPL22L1	ribosomal protein L22-like 1	0.64	0.0418
METTL8	methyltransferase like 8	0.65	0.00365
CUL2	cullin 2	0.65	0.0378
RC3H1	ring finger and CCCH-type domains 1	0.65	0.011
TMEM59	transmembrane protein 59	0.65	0.0299
PSMG1	proteasome (prosome, macropain) assembly chaperone 1	0.65	0.0494
MFSD14A	major facilitator superfamily domain containing 14A	0.65	0.00637
DHX36	DEAH (Asp-Glu-Ala-His) box polypeptide 36	0.65	0.00403
ANO4	anoctamin 4	0.65	0.00339
TP53INP1	tumor protein p53 inducible nuclear protein 1	0.66	0.0392
ALG8	ALG8, alpha-1,3-glucosyltransferase	0.66	0.0151
CHSY3	chondroitin sulfate synthase 3	0.66	0.0264
ZNF766	zinc finger protein 766	0.66	0.00564
ANXA5	annexin A5	0.66	0.0276
MRPL14	mitochondrial ribosomal protein L14	0.66	0.048
CMC1	C-x(9)-C motif containing 1	0.67	0.0304
OLA1	Obg-like ATPase 1	0.67	0.0239
SENP6	SUMO1/sentrin specific peptidase 6	0.67	0.0285
TRAPPC6B	trafficking protein particle complex 6B	0.67	0.042
CFAP36	cilia and flagella associated protein 36	0.67	0.00751
ARFGEF1	ADP-ribosylation factor guanine nucleotide-exchange factor 1 (brefeldin A-	0.67	0.0208
ZNF567	zinc finger protein 567	0.67	0.00456
MAMLD1	mastermind-like domain containing 1	0.67	0.009
SETD9	SET domain containing 9	0.67	0.00429
ZNF616	zinc finger protein 616	0.67	0.0066

LUM	lumican	0.67	0.0425
COA7	cytochrome c oxidase assembly factor 7 (putative)	0.67	0.0041
CHD1	chromodomain helicase DNA binding protein 1	0.67	0.0276
XRCC4	X-ray repair complementing defective repair in Chinese hamster cells 4	0.68	0.0123
EEA1	early endosome antigen 1	0.68	0.0345
DPP8	dipeptidyl-peptidase 8	0.68	0.00616
GAPVD1	GTPase activating protein and VPS9 domains 1	0.68	0.0355
NDUFA8	NADH dehydrogenase (ubiquinone) 1 alpha subcomplex, 8, 19kDa	0.68	0.034
CDK8	cyclin-dependent kinase 8	0.68	0.0269
DCAF16	DDB1 and CUL4 associated factor 16	0.68	0.0119
PMPCA	peptidase (mitochondrial processing) alpha	0.68	0.0258
UBXN8	UBX domain protein 8	0.68	0.0148
RASA1	RAS p21 protein activator (GTPase activating protein) 1	0.69	0.0111
BIN3	bridging integrator 3	0.69	0.0167
GTPBP8	GTP-binding protein 8 (putative)	0.69	0.0373
RHOT1	ras homolog family member T1	0.69	0.015
ERCC6	excision repair cross-complementation group 6	0.69	0.0115
GRSF1	G-rich RNA sequence binding factor 1	0.69	0.0395
CDC123	cell division cycle 123	0.70	0.02
GBP1	guanylate binding protein 1, interferon-inducible	0.70	0.0282
SEC11C	SEC11 homolog C, signal peptidase complex subunit	0.70	0.0235
IDH1	isocitrate dehydrogenase 1 (NADP+)	0.70	0.0289
PXMP4	peroxisomal membrane protein 4	0.70	0.0134
NSL1	NSL1, MIS12 kinetochore complex component	0.70	0.0153
WDR37	WD repeat domain 37	0.70	0.0217
EOGT	EGF domain-specific O-linked N-acetylglucosamine (GlcNAc) transferase	0.70	0.0198
PPP2R5E	protein phosphatase 2, regulatory subunit B, epsilon isoform	0.70	0.0208
HSD17B7	hydroxysteroid (17-beta) dehydrogenase 7	0.70	0.0218
UPF2	UPF2 regulator of nonsense transcripts homolog (yeast)	0.70	0.00801
MOSPD2	motile sperm domain containing 2	0.70	0.0147
HOXD4	homeobox D4	0.71	0.00645
PIGL	phosphatidylinositol glycan anchor biosynthesis class L	0.71	0.0199
GLRX3	glutaredoxin 3	0.71	0.0253
KDM5B	lysine (K)-specific demethylase 5B	0.71	0.0164
BMPR2	bone morphogenetic protein receptor type II	0.71	0.0403
BMPR1B	bone morphogenetic protein receptor type IB	0.71	0.013
PFDN4	prefoldin subunit 4	0.71	0.0286
HACD2	3-hydroxyacyl-CoA dehydratase 2	0.71	0.0437
CYP51A1	cytochrome P450, family 51, subfamily A, polypeptide 1	0.71	0.0282
MTA3	metastasis associated 1 family member 3	0.71	0.0209
CHSY1	chondroitin sulfate synthase 1	0.71	0.021
PDGFC	platelet derived growth factor C	0.71	0.0265
TXNIP	thioredoxin interacting protein	0.72	0.0194
EFR3A	EFR3 homolog A	0.72	0.0106
ARMC9	armadillo repeat containing 9	0.72	0.0388
TMEM203	transmembrane protein 203	0.72	0.0494
FKTN	fukutin	0.72	0.0313
RHOBTB3	Rho-related BTB domain containing 3	0.72	0.0254
SDHAF1	succinate dehydrogenase complex assembly factor 1	0.72	0.0319
ANKRD1	ankyrin repeat domain 1 (cardiac muscle)	0.72	0.0405
EXT1	exostosin glycosyltransferase 1	0.72	0.0159
CFAP20	cilia and flagella associated protein 20	0.72	0.0225
ZMYM2	zinc finger, MYM-type 2	0.72	0.0474
APPBP2	amyloid beta precursor protein (cytoplasmic tail) binding protein 2	0.72	0.0439
MANSC1	MANSC domain containing 1	0.72	0.0143
GREB1L	growth regulation by estrogen in breast cancer-like	0.72	0.0264
ACYP1	acylphosphatase 1, erythrocyte (common) type	0.72	0.0424
EPPK1	epiplakin 1	0.72	0.0142
PPP3CB	protein phosphatase 3, catalytic subunit, beta isozyme	0.72	0.044

TRIM16	tripartite motif containing 16	0.72	0.0314
IWS1	IWS1 homolog (S. cerevisiae)	0.72	0.0247
UBE2W	ubiquitin-conjugating enzyme E2W (putative)	0.72	0.023
TBC1D19	TBC1 domain family, member 19	0.72	0.035
PARP4	poly(ADP-ribose) polymerase family member 4	0.72	0.0225
OSBPL10	oxysterol binding protein-like 10	0.72	0.0118
GLO1	glyoxalase I	0.72	0.0251
PARP1	poly(ADP-ribose) polymerase 1	0.72	0.0229
PPP1CB	protein phosphatase 1, catalytic subunit, beta isozyme	0.72	0.0469
HCN1	hyperpolarization activated cyclic nucleotide gated potassium channel 1	0.72	0.0194
GPNMB	glycoprotein (transmembrane) nmb	0.72	0.034
TRUB1	TruB pseudouridine (psi) synthase family member 1	0.73	0.0221
TOP2A	topoisomerase (DNA) II alpha	0.73	0.0379
KIF9	kinesin family member 9	0.73	0.0247
PARP14	poly(ADP-ribose) polymerase family member 14	0.73	0.0403
UCP2	uncoupling protein 2 (mitochondrial, proton carrier)	0.73	0.0234
RAD51AP1	RAD51 associated protein 1	0.73	0.0165
FAM216A	family with sequence similarity 216, member A	0.73	0.031
MPRIP	myosin phosphatase Rho interacting protein	0.73	0.0293
PCDH12	protocadherin 12	0.73	0.0311
PPID	peptidylprolyl isomerase D	0.73	0.0177
HDAC1	histone deacetylase 1	0.73	0.021
LZTR1	leucine-zipper-like transcription regulator 1	0.73	0.0295
TAF7	TAF7 RNA polymerase II, TATA box binding protein (TBP)-associated	0.73	0.03
TNNI3K	TNNI3 interacting kinase	0.73	0.0258
ACTC1	actin, alpha, cardiac muscle 1	0.73	0.0264
VAMP3	vesicle associated membrane protein 3	0.73	0.0254
ARPC4	actin related protein 2/3 complex subunit 4	0.74	0.0322
TPRKB	TP53RK binding protein	0.74	0.0246
IAH1	isoamyl acetate-hydrolyzing esterase 1 homolog	0.74	0.0483
CCT3	chaperonin containing TCP1, subunit 3 (gamma)	0.74	0.0331
GPC4	glypican 4	0.74	0.0234
ARL4A	ADP-ribosylation factor like GTPase 4A	0.74	0.0437
LMO1	LIM domain only 1 (rhombotin 1)	0.74	0.0262
RNF13	ring finger protein 13	0.74	0.0472
CCNL2	cyclin L2	0.74	0.0303
IFNAR2	interferon (alpha, beta and omega) receptor 2	0.74	0.0385
STK38	serine/threonine kinase 38	0.74	0.0228
C2orf49	chromosome 2 open reading frame 49	0.74	0.0458
NEO1	neogenin 1	0.74	0.0402
SNX27	sorting nexin family member 27	0.74	0.031
ING5	inhibitor of growth family member 5	0.74	0.0364
SERGEF	secretion regulating guanine nucleotide exchange factor	0.74	0.049
TRIM52	tripartite motif containing 52	0.74	0.0449
ANKRD42	ankyrin repeat domain 42	0.74	0.0365
SIMC1	SUMO-interacting motifs containing 1	0.75	0.0408
CNIH1	cornichon family AMPA receptor auxiliary protein 1	0.75	0.0453
PPP1R2	protein phosphatase 1, regulatory (inhibitor) subunit 2	0.75	0.0382
TGDS	TDP-glucose 4,6-dehydratase	0.75	0.0251
TM2D1	TM2 domain containing 1	0.75	0.0348
PRPF18	pre-mRNA processing factor 18	0.75	0.0237
SNCAIP	synuclein alpha interacting protein	0.75	0.0434
JMY	junction mediating and regulatory protein, p53 cofactor	0.75	0.0263
DIRAS3	DIRAS family, GTP-binding RAS-like 3	0.75	0.0493
INPP1	inositol polyphosphate-1-phosphatase	0.75	0.0471
C1orf116	chromosome 1 open reading frame 116	0.75	0.0333
CEMIP	cell migration inducing protein, hyaluronan binding	0.75	0.0382
ARL8B	ADP-ribosylation factor like GTPase 8B	0.75	0.0382
PTPRG	protein tyrosine phosphatase, receptor type, G	0.75	0.0359

ZRANB1	zinc finger, RAN-binding domain containing 1	0.76	0.0329
SEPHS2	selenophosphate synthetase 2	0.76	0.0373
CISD2	CDGSH iron sulfur domain 2	0.76	0.0396
RIPK1	receptor (TNFRSF)-interacting serine-threonine kinase 1	0.76	0.0377
ASCC3	activating signal cointegrator 1 complex subunit 3	0.76	0.0326
DMWD	dystrophia myotonica, WD repeat containing	0.76	0.0366
NRIP3	nuclear receptor interacting protein 3	0.76	0.0315
PHGDH	phosphoglycerate dehydrogenase	0.76	0.0279
NSD1	nuclear receptor binding SET domain protein 1	0.76	0.0487
ELMO2	engulfment and cell motility 2	0.76	0.0365
DIXDC1	DIX domain containing 1	0.76	0.0373
ATP6V1C2	ATPase, H+ transporting, lysosomal 42kDa, V1 subunit C2	0.76	0.0466
VTI1B	vesicle transport through interaction with t-SNAREs 1B	0.76	0.0484
METTL2B	methyltransferase like 2B	0.76	0.0407
OCRL	oculocerebrorenal syndrome of Lowe	0.77	0.0384
PHLPP2	PH domain and leucine rich repeat protein phosphatase 2	0.77	0.0415
EDA2R	ectodysplasin A2 receptor	0.77	0.0446
B3GALT4	UDP-Gal:betaGlcNAc beta 1,3-galactosyltransferase 4	0.77	0.0338
TMEM25	transmembrane protein 25	0.77	0.0351
ZNF880	zinc finger protein 880	0.77	0.0404
PHLDB2	pleckstrin homology-like domain, family B, member 2	0.77	0.0403
TUBAL3	tubulin, alpha-like 3	0.77	0.0422
PTPN3	protein tyrosine phosphatase, non-receptor type 3	0.77	0.0408
SKIL	SKI-like proto-oncogene	0.78	0.0497
PHF6	PHD finger protein 6	0.78	0.0416

Table S5. IDMF-increased genes (P < 0.05 with FC > 1.25). The table lists 673 IDMF-increased genes ranked by estimated fold-change (FC) (IDMF/CTL). The gene symbol and description are shown. The raw p-value is listed in the final column (generalized least square linear models with moderated t-statistics; *n* = 3 samples per group; *FDR < 0.10).

Symbol	Description	FC	P-value
ALDH1L1	aldehyde dehydrogenase 1 family, member L1	2.37	1.93e-05*
C9orf24	chromosome 9 open reading frame 24	1.91	8.14e-05*
OSGIN1	oxidative stress induced growth inhibitor 1	1.88	3.46e-05*
ZDHHC11B	zinc finger, DHHC-type containing 11B	1.85	8.42e-05*
OIT3	oncoprotein induced transcript 3	1.81	6.88e-05*
LPAR2	lysophosphatidic acid receptor 2	1.79	0.000161*
CDRT1	CMT1A duplicated region transcript 1	1.76	0.000131*
OR2M7	olfactory receptor, family 2, subfamily M, member 7	1.71	0.000324
LY6G6C	lymphocyte antigen 6 complex, locus G6C	1.70	0.000394
ALG1	ALG1, chitobiosyldiphosphodolichol beta-mannosyltransferase	1.69	0.000793
C9orf131	chromosome 9 open reading frame 131	1.65	0.000398
SMIM6	small integral membrane protein 6	1.63	0.00235
ATF3	activating transcription factor 3	1.62	0.000446
KNDC1	Transcript Identified by AceView, Entrez Gene ID(s) 85442	1.60	0.00765
HCAR3	hydroxycarboxylic acid receptor 3	1.60	0.00216
ULK3	unc-51 like kinase 3	1.60	0.00113
P4HTM	prolyl 4-hydroxylase, transmembrane (endoplasmic reticulum)	1.60	0.00227
TYR	tyrosinase	1.60	0.00171
MCEMP1	mast cell-expressed membrane protein 1	1.59	0.000766
SPATA25	spermatogenesis associated 25	1.59	0.0018
LSMEM1	leucine-rich single-pass membrane protein 1	1.59	0.000802
ALPI	alkaline phosphatase, intestinal	1.59	0.00108
MXD1	MAX dimerization protein 1	1.58	0.00104
TLX1	T-cell leukemia homeobox 1	1.58	0.0145
TMEM174	transmembrane protein 174	1.58	0.00195
ISX	intestine-specific homeobox	1.58	0.0122
CCL16	chemokine (C-C motif) ligand 16	1.58	0.00133
NLRP9	NLR family, pyrin domain containing 9	1.57	0.0017
LRRC46	leucine rich repeat containing 46	1.57	0.00222
DOK4	docking protein 4	1.57	0.00185
GATA4	GATA binding protein 4	1.57	0.000767
TRIM73	tripartite motif containing 73	1.57	0.000989
TMEM39B	transmembrane protein 39B	1.56	0.00181
KCTD21	potassium channel tetramerization domain containing 21	1.55	0.00177
BEST1	bestrophin 1	1.55	0.00152
SLC5A5	solute carrier family 5 (sodium/iodide cotransporter), member 5	1.55	0.00454
JPH3	junctophilin 3	1.54	0.00311
DMRTC1	DMRT-like family C1	1.54	0.00479
CYP17A1	cytochrome P450, family 17, subfamily A, polypeptide 1	1.53	0.00187
HMOX1	heme oxygenase 1	1.53	0.0014
CD33	CD33 molecule	1.53	0.00116
ISG20	interferon stimulated exonuclease gene 20kDa	1.53	0.0043
CST9	cystatin 9 (testatin)	1.53	0.00217
RASL10B	RAS-like, family 10, member B	1.52	0.00329
ARHGEF38	Rho guanine nucleotide exchange factor 38	1.52	0.00141
SEMA4D	Memczak2013 ANTISENSE, coding, INTERNAL, UTR3 best transcript	1.52	0.00429
OR9A2	olfactory receptor, family 9, subfamily A, member 2	1.52	0.00289
SH2B2	Memczak2013 ANTISENSE, CDS, coding, INTERNAL best transcript	1.51	0.0286
MYO15B	myosin XVB	1.51	0.00219
SIRT7	sirtuin 7	1.51	0.00185
FAM89A	family with sequence similarity 89, member A	1.51	0.00318
ALDH4A1	aldehyde dehydrogenase 4 family, member A1	1.51	0.00586
RBM39	RNA binding motif protein 39	1.51	0.00429
USP17L21	ubiquitin specific peptidase 17-like family member 21	1.50	0.00242

GSR	glutathione reductase	1.50	0.00203
PTHLH	parathyroid hormone-like hormone	1.50	0.00336
NBL1	neuroblastoma 1, DAN family BMP antagonist	1.50	0.00457
TGFBR3L	transforming growth factor beta receptor III like	1.50	0.00458
CENPP	centromere protein P	1.50	0.00674
SHCBP1	SHC SH2-domain binding protein 1	1.49	0.00307
MCM4	minichromosome maintenance complex component 4	1.49	0.0102
NEURL2	neuralized E3 ubiquitin protein ligase 2	1.49	0.00547
WFDC6	WAP four-disulfide core domain 6	1.49	0.00498
LRRC38	leucine rich repeat containing 38	1.49	0.00498
SLC39A2	solute carrier family 39 (zinc transporter), member 2	1.49	0.00769
NDUFAF6	NADH dehydrogenase (ubiquinone) complex I, assembly factor 6	1.48	0.0159
OR10R2	olfactory receptor, family 10, subfamily R, member 2	1.48	0.00387
GHRHR	growth hormone releasing hormone receptor	1.48	0.00324
SLC7A11	solute carrier family 7 (anionic amino acid transporter light chain, xc- system),	1.48	0.00305
CFAP46	cilia and flagella associated protein 46	1.48	0.00556
MEGF6	multiple EGF-like-domains 6	1.48	0.00417
CPEB1	cytoplasmic polyadenylation element binding protein 1	1.48	0.00754
SHANK2	SH3 and multiple ankyrin repeat domains 2	1.48	0.00644
TMED9	transmembrane p24 trafficking protein 9	1.48	0.012
PRSS58	protease, serine, 58	1.48	0.00286
FAM156A	family with sequence similarity 156, member A	1.48	0.00416
SMNDC1	survival motor neuron domain containing 1	1.48	0.00739
MCCC1	methylcrotonoyl-CoA carboxylase 1	1.48	0.0115
CMTM2	CKLF-like MARVEL transmembrane domain containing 2	1.47	0.00761
CDK2	cyclin-dependent kinase 2	1.47	0.0338
POTEB	POTE ankyrin domain family, member B	1.47	0.0103
MYL3	myosin, light chain 3, alkali; ventricular, skeletal, slow	1.47	0.00477
MICAL1	Jeck2013 ANTISENSE, coding, INTERNAL, intronic best transcript	1.47	0.00776
TRIM49C	tripartite motif containing 49C	1.47	0.00283
PPM1G	protein phosphatase, Mg2+/Mn2+ dependent, 1G	1.47	0.0101
RNF40	ring finger protein 40, E3 ubiquitin protein ligase	1.47	0.00307
SVEP1	sushi, von Willebrand factor type A, EGF and pentraxin domain containing 1	1.47	0.00279
NCS1	neuronal calcium sensor 1	1.47	0.00664
TP53INP2	tumor protein p53 inducible nuclear protein 2	1.47	0.00724
USP17L24	ubiquitin specific peptidase 17-like family member 24	1.46	0.00608
USP17L25	ubiquitin specific peptidase 17-like family member 25	1.46	0.00608
GUCY1A2	guanylate cyclase 1, soluble, alpha 2	1.46	0.00774
HAPLN3	hyaluronan and proteoglycan link protein 3	1.46	0.00677
L3MBTL4	l(3)mbt-like 4 (Drosophila)	1.46	0.0155
COLGALT2	collagen beta(1-O)galactosyltransferase 2	1.46	0.00676
RASSF2	Memczak2013 ANTISENSE, coding, INTERNAL, UTR3 best transcript	1.46	0.00643
LRRC43	leucine rich repeat containing 43	1.46	0.0197
DOC2B	double C2-like domains, beta	1.46	0.0179
OR2L2	olfactory receptor, family 2, subfamily L, member 2	1.46	0.00782
UBL4A	ubiquitin-like 4A	1.45	0.00477
IGSF8	immunoglobulin superfamily, member 8	1.45	0.00833
AFF2	AF4/FMR2 family, member 2	1.45	0.0203
PLA2G2D	phospholipase A2, group IID	1.45	0.00478
CST7	cystatin F (leukocystatin)	1.45	0.00512
OR52D1	olfactory receptor, family 52, subfamily D, member 1	1.45	0.00863
FER1L5	fer-1-like family member 5	1.45	0.00704
OR1B1	olfactory receptor, family 1, subfamily B, member 1 (gene/pseudogene)	1.45	0.00785
MTFP1	mitochondrial fission process 1	1.45	0.00902
SNRNP70	small nuclear ribonucleoprotein, U1 70kDa subunit	1.45	0.00435
RPEL1	ribulose-5-phosphate-3-epimerase-like 1	1.45	0.0217
ITGA9	integrin alpha 9	1.45	0.0108
POLE	polymerase (DNA directed), epsilon, catalytic subunit	1.45	0.00577
TNP1	transition protein 1 (during histone to protamine replacement)	1.44	0.0249

KIAA1614	KIAA1614	1.44	0.00539
SLC6A6	solute carrier family 6 (neurotransmitter transporter), member 6	1.44	0.0053
SCYL3	SCY1-like, kinase-like 3	1.44	0.00711
TMEM175	transmembrane protein 175	1.44	0.00566
CDH18	cadherin 18, type 2	1.44	0.00983
ACSL6	acyl-CoA synthetase long-chain family member 6	1.44	0.011
SERPINE3	serpin peptidase inhibitor, clade E (nexin, plasminogen activator inhibitor type 1),	1.44	0.00791
TRIOBP	TRIO and F-actin binding protein	1.44	0.0113
EGR1	early growth response 1	1.44	0.00911
KIF26A	kinesin family member 26A	1.44	0.0087
GPR101	G protein-coupled receptor 101	1.44	0.0111
USP17L27	ubiquitin specific peptidase 17-like family member 27	1.43	0.00939
USP17L29	ubiquitin specific peptidase 17-like family member 29	1.43	0.00939
HLA-DRB5	major histocompatibility complex, class II, DR beta 5	1.43	0.00696
CAV3	caveolin 3	1.43	0.0246
BMP7	bone morphogenetic protein 7	1.43	0.0113
TRPV6	transient receptor potential cation channel, subfamily V, member 6	1.43	0.00579
PPP1R10	protein phosphatase 1, regulatory subunit 10	1.43	0.00599
DYRK1A	dual specificity tyrosine-(Y)-phosphorylation regulated kinase 1A	1.43	0.00797
POP4	POP4 homolog, ribonuclease P/MRP subunit	1.43	0.0142
FAT2	FAT atypical cadherin 2	1.43	0.0106
BTNL2	butyrophilin-like 2	1.43	0.0158
ZNF778	zinc finger protein 778	1.43	0.0164
RFFL	ring finger and FYVE-like domain containing E3 ubiquitin protein ligase	1.43	0.0113
DPYD	dihydropyrimidine dehydrogenase	1.42	0.0103
PKNOX2	PBX/knotted 1 homeobox 2	1.42	0.00893
FOXC2	forkhead box C2	1.42	0.0175
APOBEC2	apolipoprotein B mRNA editing enzyme, catalytic polypeptide-like 2	1.42	0.00709
TLCD2	TLC domain containing 2	1.42	0.0159
CCL3	chemokine (C-C motif) ligand 3	1.42	0.0172
EVPL	envoplakin	1.42	0.00828
WFDC12	WAP four-disulfide core domain 12	1.42	0.00653
FRG2B	FSHD region gene 2 family, member B	1.42	0.00842
GP9	glycoprotein IX (platelet)	1.42	0.0107
ABCA4	ATP binding cassette subfamily A member 4	1.42	0.013
SAMD4A	sterile alpha motif domain containing 4A	1.42	0.0119
RASL11A	RAS-like, family 11, member A	1.42	0.00866
OR4K14	olfactory receptor, family 4, subfamily K, member 14	1.42	0.0143
DMRTC1B	DMRT-like family C1B	1.42	0.0185
KLHL33	kelch-like family member 33	1.42	0.00636
DNLZ	DNL-type zinc finger	1.42	0.00762
C10orf95	chromosome 10 open reading frame 95	1.42	0.0121
SNAPC4	Zhang2013 ALT_ACCEPTOR, ALT_DONOR, coding, INTERNAL, intronic	1.42	0.0161
CD14	CD14 molecule	1.41	0.0249
GAGE4	G antigen 4	1.41	0.0158
ZFP62	ZFP62 zinc finger protein	1.41	0.0155
TCP11X2	t-complex 11 family, X-linked 2	1.41	0.0106
TIGD5	tigger transposable element derived 5	1.41	0.0227
ADAP1	ArfGAP with dual PH domains 1	1.41	0.00872
ZNF671	zinc finger protein 671	1.41	0.0121
SLC5A2	solute carrier family 5 (sodium/glucose cotransporter), member 2	1.41	0.0133
LY6G5C	lymphocyte antigen 6 complex, locus G5C	1.41	0.0118
LMX1A	LIM homeobox transcription factor 1, alpha	1.41	0.00677
TRIM49B	tripartite motif containing 49B	1.41	0.0135
TMIE	transmembrane inner ear	1.41	0.0164
SLC22A14	solute carrier family 22, member 14	1.41	0.0177
APOC2	apolipoprotein C-II	1.41	0.0124
HSPBP1	HSPA (heat shock 70kDa) binding protein, cytoplasmic cochaperone 1	1.41	0.00832
RPRML	reprimo-like	1.41	0.0073

DCX	doublecortin	1.41	0.00841
OR52M1	olfactory receptor, family 52, subfamily M, member 1	1.41	0.0146
MC1R	melanocortin 1 receptor (alpha melanocyte stimulating hormone receptor)	1.41	0.0128
SLC24A1	solute carrier family 24 (sodium/potassium/calcium exchanger), member 1	1.41	0.00838
FGF10	fibroblast growth factor 10	1.41	0.00839
CLDN18	claudin 18	1.41	0.0151
CNPPD1	cyclin Pas1/PHO80 domain containing 1	1.41	0.00864
CES1	carboxylesterase 1	1.41	0.0218
FAM13A	family with sequence similarity 13, member A	1.41	0.00736
TBX5	T-box 5	1.41	0.00905
GTSF1L	gametocyte specific factor 1-like	1.41	0.0098
SLC39A7	solute carrier family 39 (zinc transporter), member 7	1.41	0.00901
C1QA	complement component 1, q subcomponent, A chain	1.41	0.0129
CARD14	caspase recruitment domain family, member 14	1.41	0.0189
TAZ	tafazzin	1.41	0.00645
KRTAP20-1	keratin associated protein 20-1	1.40	0.0133
COA1	cytochrome c oxidase assembly factor 1 homolog	1.40	0.0386
RBM26	Memczak2013 ANTISENSE, CDS, coding, INTERNAL best transcript	1.40	0.028
BCAT2	branched chain amino-acid transaminase 2, mitochondrial	1.40	0.0087
MYCL	v-myc avian myelocytomatosis viral oncogene lung carcinoma derived homolog	1.40	0.012
KRTAP23-1	keratin associated protein 23-1	1.40	0.021
CDK5R1	cyclin-dependent kinase 5, regulatory subunit 1 (p35)	1.40	0.0192
NUP35	nucleoporin 35kDa	1.40	0.012
ZNF366	zinc finger protein 366	1.40	0.014
NR1D1	nuclear receptor subfamily 1, group D, member 1	1.40	0.011
CADM4	cell adhesion molecule 4	1.40	0.0126
TRIM74	tripartite motif containing 74	1.40	0.0098
SNX8	sorting nexin 8	1.40	0.0147
HES3	hes family bHLH transcription factor 3	1.40	0.0183
FOXI1	forkhead box I1	1.40	0.0217
SLC29A4	solute carrier family 29 (equilibrative nucleoside transporter), member 4	1.40	0.023
AQP7	aquaporin 7	1.40	0.0125
KDM4E	lysine (K)-specific demethylase 4E	1.40	0.0103
C3orf35	chromosome 3 open reading frame 35	1.40	0.0242
BMP10	bone morphogenetic protein 10	1.40	0.0154
ETV2	ets variant 2	1.40	0.0161
PRB1	proline-rich protein BstNI subfamily 1	1.39	0.0184
PTPRN	protein tyrosine phosphatase, receptor type, N	1.39	0.0256
SLC7A6	solute carrier family 7 (amino acid transporter light chain, y+L system), member	1.39	0.0115
HNRNPF	heterogeneous nuclear ribonucleoprotein F	1.39	0.0162
ADAM22	ADAM metallopeptidase domain 22	1.39	0.0293
ESRRG	estrogen-related receptor gamma	1.39	0.00989
SPNS1	spinster homolog 1 (Drosophila)	1.39	0.0119
OLFM4	olfactomedin 4	1.39	0.0104
DNASE1	deoxyribonuclease I	1.39	0.0123
CHST5	carbohydrate (N-acetylglucosamine 6-O) sulfotransferase 5	1.39	0.014
PLAC8L1	PLAC8-like 1	1.39	0.0198
CNP	Memczak2013 ANTISENSE, CDS, coding, INTERNAL best transcript	1.39	0.0139
SLC1A2	solute carrier family 1 (glial high affinity glutamate transporter), member 2	1.39	0.0105
SRXN1	sulfiredoxin 1	1.39	0.0143
SMCO2	single-pass membrane protein with coiled-coil domains 2	1.39	0.0213
FAM166A	family with sequence similarity 166, member A	1.39	0.0167
MC2R	melanocortin 2 receptor (adrenocorticotropic hormone)	1.39	0.0143
SLC17A6	solute carrier family 17 (vesicular glutamate transporter), member 6	1.39	0.0122
PRRG2	proline rich Gla (G-carboxyglutamic acid) 2	1.39	0.0363
KLHL3	kelch-like family member 3	1.39	0.0139
PDE3A	phosphodiesterase 3A, cGMP-inhibited	1.39	0.018
LMO3	LIM domain only 3 (rhombotin-like 2)	1.39	0.0143
PIK3R5	phosphoinositide-3-kinase, regulatory subunit 5	1.39	0.0238

DUX4	double homeobox 4	1.39	0.0217
BEAN1	brain expressed, associated with NEDD4, 1	1.39	0.0255
SCAF1	SR-related CTD-associated factor 1	1.39	0.0179
MDC1	mediator of DNA-damage checkpoint 1	1.39	0.0204
CBFA2T3	core-binding factor, runt domain, alpha subunit 2; translocated to, 3	1.39	0.0162
TKT	transketolase	1.39	0.00914
APOBEC3H	apolipoprotein B mRNA editing enzyme, catalytic polypeptide-like 3H	1.38	0.016
LOC730183	uncharacterized LOC730183	1.38	0.0312
CSAD	cysteine sulfinic acid decarboxylase	1.38	0.0224
C8orf34	chromosome 8 open reading frame 34	1.38	0.0147
OSBP2	oxysterol binding protein 2	1.38	0.0292
CHRNA1	cholinergic receptor, nicotinic alpha 1	1.38	0.0226
C1QTNF8	C1q and tumor necrosis factor related protein 8	1.38	0.0104
ALKBH4	alkB homolog 4, lysine demthylase	1.38	0.0461
MRRF	mitochondrial ribosome recycling factor	1.38	0.0118
MST1L	macrophage stimulating 1-like	1.38	0.0141
PLA2G5	phospholipase A2, group V	1.38	0.0149
LGI1	leucine-rich, glioma inactivated 1	1.38	0.0161
FAM71F1	family with sequence similarity 71, member F1	1.38	0.0234
PTOV1	prostate tumor overexpressed 1	1.38	0.0233
ZNF233	zinc finger protein 233	1.38	0.0173
AVPI1	arginine vasopressin-induced 1	1.38	0.0138
SLC6A4	solute carrier family 6 (neurotransmitter transporter), member 4	1.38	0.0144
F10	coagulation factor X	1.38	0.0278
CST2	cystatin SA	1.38	0.0163
SYT4	synaptotagmin IV	1.38	0.0215
SELPLG	selectin P ligand	1.38	0.0183
RAB3IL1	RAB3A interacting protein (rabin3)-like 1	1.38	0.0128
CKMT1A	creatine kinase, mitochondrial 1A	1.38	0.0329
C1orf53	chromosome 1 open reading frame 53	1.38	0.0126
HSFX2	heat shock transcription factor family, X-linked 2	1.38	0.0104
LAPTM5	lysosomal protein transmembrane 5	1.38	0.0123
ST6GALNAC1	ST6 (alpha-N-acetyl-neuraminyl-2,3-beta-galactosyl-1,3)-N-	1.38	0.0137
AOC2	amine oxidase, copper containing 2 (retina-specific)	1.38	0.0144
NUDT7	nudix hydrolase 7	1.38	0.0114
OR4A5	olfactory receptor, family 4, subfamily A, member 5	1.37	0.022
TSPAN7	tetraspanin 7	1.37	0.0161
SPRR2E	small proline-rich protein 2E	1.37	0.019
PBXIP1	pre-B-cell leukemia homeobox interacting protein 1	1.37	0.0133
XAGE1B	X antigen family, member 1B	1.37	0.0131
ALAD	aminolevulinate dehydratase	1.37	0.019
DAPL1	death associated protein like 1	1.37	0.0203
IDH3A	isocitrate dehydrogenase 3 (NAD+) alpha	1.37	0.0241
IGSF22	immunoglobulin superfamily, member 22	1.37	0.0294
TPPP3	tubulin polymerization-promoting protein family member 3	1.37	0.0452
MUC16	mucin 16, cell surface associated	1.37	0.0227
OTOL1	otolin 1	1.37	0.0163
ACVR1	activin A receptor type I	1.37	0.0185
SCGB1D4	secretoglobin, family 1D, member 4	1.37	0.0212
ELOVL5	ELOVL fatty acid elongase 5	1.37	0.0162
SPP1	secreted phosphoprotein 1	1.37	0.0123
USP17L28	ubiquitin specific peptidase 17-like family member 28	1.37	0.0216
LDHC	lactate dehydrogenase C	1.37	0.0261
FAM219A	family with sequence similarity 219, member A	1.37	0.0322
TEX9	testis expressed 9	1.37	0.0159
ADPRHL1	ADP-ribosylhydrolase like 1	1.37	0.0119
POLR2D	polymerase (RNA) II (DNA directed) polypeptide D	1.37	0.0253
FGF8	fibroblast growth factor 8 (androgen-induced)	1.37	0.0304
TMEM128	transmembrane protein 128	1.37	0.0236

OR2T27	olfactory receptor, family 2, subfamily T, member 27	1.37	0.0172
MAGEB10	MAGE family member B10	1.37	0.0155
LY6D	lymphocyte antigen 6 complex, locus D	1.37	0.018
USP17L26	ubiquitin specific peptidase 17-like family member 26	1.37	0.0214
SRD5A1	steroid-5-alpha-reductase, alpha polypeptide 1 (3-oxo-5 alpha-steroid delta 4-	1.37	0.0395
DEGS2	delta(4)-desaturase, sphingolipid 2	1.36	0.0168
MYLK3	myosin light chain kinase 3	1.36	0.018
FABP6	fatty acid binding protein 6, ileal	1.36	0.0178
MLLT11	myeloid/lymphoid or mixed-lineage leukemia; translocated to, 11	1.36	0.0185
ACSM2B	acyl-CoA synthetase medium-chain family member 2B	1.36	0.0248
RIBC1	RIB43A domain with coiled-coils 1	1.36	0.0143
IL1R2	interleukin 1 receptor, type II	1.36	0.0214
GPR6	G protein-coupled receptor 6	1.36	0.0196
ELAC1	elaC ribonuclease Z 1	1.36	0.029
PCSK6	proprotein convertase subtilisin/kexin type 6	1.36	0.0414
CCDC154	coiled-coil domain containing 154	1.36	0.0159
OR52E8	olfactory receptor, family 52, subfamily E, member 8	1.36	0.0149
ISL1	Transcript Identified by AceView, Entrez Gene ID(s) 3670	1.36	0.022
ARHGEF39	Rho guanine nucleotide exchange factor 39	1.36	0.0187
FOSB	FBJ murine osteosarcoma viral oncogene homolog B	1.36	0.0263
CBY3	chibby homolog 3 (Drosophila)	1.36	0.021
PREX1	Memczak2013 ALT_ACCEPTOR, ALT_DONOR, coding, INTERNAL, intronic	1.36	0.0298
NAT10	N-acetyltransferase 10 (GCN5-related)	1.36	0.0199
DISC1	disrupted in schizophrenia 1	1.36	0.0233
DEFA3	defensin, alpha 3, neutrophil-specific	1.36	0.0426
HNRNPCL2	heterogeneous nuclear ribonucleoprotein C-like 2	1.36	0.0175
PGS1	phosphatidylglycerophosphate synthase 1	1.36	0.0258
RARRES2	retinoic acid receptor responder (tazarotene induced) 2	1.36	0.0224
CSF3	colony stimulating factor 3	1.36	0.0278
COQ2	coenzyme Q2 4-hydroxybenzoate polyprenyltransferase	1.36	0.0387
SLC4A8	solute carrier family 4, sodium bicarbonate cotransporter, member 8	1.36	0.0218
RAPGEF3	Rap guanine nucleotide exchange factor 3	1.36	0.0176
AVPR1B	arginine vasopressin receptor 1B	1.36	0.0235
SERTAD3	SERTA domain containing 3	1.36	0.0236
DEFB106A	defensin, beta 106A	1.36	0.0364
KRT38	keratin 38, type I	1.36	0.0273
MTURN	maturin, neural progenitor differentiation regulator homolog (Xenopus)	1.36	0.0286
PCP2	Purkinje cell protein 2	1.36	0.0318
CEP170B	centrosomal protein 170B	1.36	0.0224
ADGRE2	adhesion G protein-coupled receptor E2	1.36	0.0153
ASAP3	ArfGAP with SH3 domain, ankyrin repeat and PH domain 3	1.36	0.0354
ANGPTL4	angiopoietin like 4	1.36	0.0222
TRMT1	tRNA methyltransferase 1	1.35	0.0449
LCE5A	late cornified envelope 5A	1.35	0.0203
HDAC10	histone deacetylase 10	1.35	0.0246
ZFHX3	zinc finger homeobox 3	1.35	0.0185
NUBP2	nucleotide binding protein 2	1.35	0.016
USP17L22	ubiquitin specific peptidase 17-like family member 22	1.35	0.0214
EPHA10	EPH receptor A10	1.35	0.0183
SLC28A2	solute carrier family 28 (concentrative nucleoside transporter), member 2	1.35	0.0185
HNRNPA1L2	heterogeneous nuclear ribonucleoprotein A1-like 2	1.35	0.0185
IL20RB	interleukin 20 receptor beta	1.35	0.0213
DAND5	DAN domain family member 5, BMP antagonist	1.35	0.0217
CNR2	cannabinoid receptor 2	1.35	0.0153
DTX2	deltex 2, E3 ubiquitin ligase	1.35	0.0303
PROP1	PROP paired-like homeobox 1	1.35	0.0253
OR7D2	olfactory receptor, family 7, subfamily D, member 2	1.35	0.0275
ENO4	enolase family member 4	1.35	0.0178
FGB	fibrinogen beta chain	1.35	0.0198

SPACA5B	sperm acrosome associated 5B	1.35	0.0311
AKNA	AT-hook transcription factor	1.35	0.0471
CPNE2	copine II	1.35	0.0258
LRP1B	LDL receptor related protein 1B	1.35	0.0475
VSIG8	V-set and immunoglobulin domain containing 8	1.35	0.027
OPA3	optic atrophy 3 (autosomal recessive, with chorea and spastic paraplegia)	1.35	0.0284
GMPR	guanosine monophosphate reductase	1.35	0.02
CNIH2	cornichon family AMPA receptor auxiliary protein 2	1.35	0.0369
XYLT2	xylosyltransferase II	1.35	0.0212
LCE3B	late cornified envelope 3B	1.35	0.0345
ITLN1	intelectin 1 (galactofuranose binding)	1.35	0.0306
GNG8	guanine nucleotide binding protein (G protein), gamma 8	1.35	0.0344
GREB1	growth regulation by estrogen in breast cancer 1	1.35	0.034
ZNF582	zinc finger protein 582	1.35	0.0162
OR4K13	olfactory receptor, family 4, subfamily K, member 13	1.35	0.019
PLA2G10	phospholipase A2, group X	1.35	0.0352
SLC22A9	solute carrier family 22 (organic anion transporter), member 9	1.35	0.0308
MAOA	monoamine oxidase A	1.35	0.0355
LIMCH1	LIM and calponin homology domains 1	1.35	0.0431
FAM71A	family with sequence similarity 71, member A	1.35	0.0432
ARID1B	Memczak2013 ANTISENSE, CDS, coding, INTERNAL best transcript	1.35	0.0499
KRTAP3-2	keratin associated protein 3-2	1.35	0.0451
IGFN1	immunoglobulin-like and fibronectin type III domain containing 1	1.35	0.0242
FUT9	fucosyltransferase 9 (alpha (1,3) fucosyltransferase)	1.35	0.0288
FOLH1	folate hydrolase (prostate-specific membrane antigen) 1	1.35	0.028
SLC35F3	solute carrier family 35, member F3	1.35	0.0393
CCDC42	coiled-coil domain containing 42	1.35	0.0409
ZNF831	zinc finger protein 831	1.34	0.039
GRK7	G protein-coupled receptor kinase 7	1.34	0.0211
DPH5	diphthamide biosynthesis 5	1.34	0.0278
TBXAS1	thromboxane A synthase 1 (platelet)	1.34	0.0243
ULBP2	UL16 binding protein 2	1.34	0.0193
MEX3B	mex-3 RNA binding family member B	1.34	0.0169
OR6B2	olfactory receptor, family 6, subfamily B, member 2	1.34	0.0353
CREG2	cellular repressor of E1A-stimulated genes 2	1.34	0.0261
PTMS	parathymosin	1.34	0.0259
ZPBP2	zona pellucida binding protein 2	1.34	0.0247
MLN	motilin	1.34	0.032
ARNT	aryl hydrocarbon receptor nuclear translocator	1.34	0.0278
MTMR11	myotubularin related protein 11	1.34	0.0265
GIMAP6	GTPase, IMAP family member 6	1.34	0.0471
FANCA	Fanconi anemia complementation group A	1.34	0.0186
HAP1	huntingtin-associated protein 1	1.34	0.0332
DPEP2	dipeptidase 2	1.34	0.0286
TMBIM1	transmembrane BAX inhibitor motif containing 1	1.34	0.0299
CAPN12	calpain 12	1.34	0.0237
WDR7	WD repeat domain 7	1.34	0.0266
TBATA	thymus, brain and testes associated	1.34	0.0209
ZNF284	zinc finger protein 284	1.34	0.0295
ZNF214	zinc finger protein 214	1.34	0.0324
ADGRF3	adhesion G protein-coupled receptor F3	1.34	0.0319
AANAT	aralkylamine N-acetyltransferase	1.34	0.0175
MED14	mediator complex subunit 14	1.34	0.0437
FXYD7	FXYD domain containing ion transport regulator 7	1.34	0.0237
PNPLA1	patatin-like phospholipase domain containing 1	1.34	0.047
ACTR5	ARP5 actin-related protein 5 homolog (yeast)	1.34	0.0231
SOX30	SRY box 30	1.34	0.0326
OMP	olfactory marker protein	1.34	0.0197
ZBED9	zinc finger, BED-type containing 9	1.34	0.0286

GAGE1	G antigen 1	1.34	0.0427
CFC1B	cripto, FRL-1, cryptic family 1B	1.34	0.026
FAM111A	family with sequence similarity 111, member A	1.34	0.0203
PC	Memczak2013 ALT_ACCEPTOR, ALT_DONOR, coding, INTERNAL, intronic	1.34	0.0304
ORAI1	ORAI calcium release-activated calcium modulator 1	1.34	0.0256
ANKMY1	ankyrin repeat and MYND domain containing 1	1.34	0.0262
IL19	interleukin 19	1.34	0.0255
FAAH	fatty acid amide hydrolase	1.34	0.0264
MIXL1	Mix paired-like homeobox	1.34	0.0311
C4orf47	chromosome 4 open reading frame 47	1.34	0.0493
DEFB106B	defensin, beta 106B	1.34	0.0303
MSH6	mutS homolog 6	1.34	0.0289
GRK6	G protein-coupled receptor kinase 6	1.34	0.0275
OXER1	oxoeicosanoid (OXE) receptor 1	1.33	0.0323
IQCH	IQ motif containing H	1.33	0.0257
ACSM6	acyl-CoA synthetase medium-chain family member 6	1.33	0.0235
PHF24	PHD finger protein 24	1.33	0.0394
KANSL1	KAT8 regulatory NSL complex subunit 1	1.33	0.0303
SCNN1A	sodium channel, non voltage gated 1 alpha subunit	1.33	0.0288
RASIP1	Ras interacting protein 1	1.33	0.0239
UBN2	ubinuclein 2	1.33	0.0288
DEFB132	defensin, beta 132	1.33	0.0275
DMBX1	diencephalon/mesencephalon homeobox 1	1.33	0.0218
ITIH4	inter-alpha-trypsin inhibitor heavy chain family, member 4	1.33	0.0227
C1QL1	complement component 1, q subcomponent-like 1	1.33	0.023
S100A3	S100 calcium binding protein A3	1.33	0.0199
KLK3	kallikrein related peptidase 3	1.33	0.0358
MALL	mal, T-cell differentiation protein-like	1.33	0.0382
SUPT20HL2	SPT20 homolog, SAGA complex component-like 2	1.33	0.0418
CYP3A7	cytochrome P450, family 3, subfamily A, polypeptide 7	1.33	0.0256
RIMS4	regulating synaptic membrane exocytosis 4	1.33	0.0349
KRTAP19-1	keratin associated protein 19-1	1.33	0.0242
C6	complement component 6	1.33	0.0283
POU6F1	POU class 6 homeobox 1	1.33	0.0307
VCAM1	vascular cell adhesion molecule 1	1.33	0.0232
OR1S2	olfactory receptor, family 1, subfamily S, member 2	1.33	0.0324
C19orf33	chromosome 19 open reading frame 33	1.33	0.0341
EYA1	EYA transcriptional coactivator and phosphatase 1	1.33	0.0345
OR2G2	olfactory receptor, family 2, subfamily G, member 2	1.33	0.0423
OR4F5	olfactory receptor, family 4, subfamily F, member 5	1.33	0.0392
C19orf84	chromosome 19 open reading frame 84	1.33	0.0429
VCY	variable charge, Y-linked	1.33	0.0299
NTF4	neurotrophin 4	1.33	0.0234
SLC22A13	solute carrier family 22 (organic anion/urate transporter), member 13	1.33	0.0224
MSMP	microseminoprotein, prostate associated	1.33	0.0296
IPO7	importin 7	1.33	0.0313
ZNF710	zinc finger protein 710	1.33	0.0434
FEV	FEV (ETS oncogene family)	1.33	0.0251
REG3G	regenerating islet-derived 3 gamma	1.33	0.0302
OTP	orthopedia homeobox	1.33	0.0334
NTSR1	neurotensin receptor 1 (high affinity)	1.33	0.0418
TPRX1	tetra-peptide repeat homeobox 1	1.33	0.0388
CDH9	cadherin 9, type 2 (T1-cadherin)	1.33	0.0372
MTHFSD	methenyltetrahydrofolate synthetase domain containing	1.33	0.0285
PELP1	proline, glutamate and leucine rich protein 1	1.33	0.0433
BCL11B	B-cell CLL/lymphoma 11B (zinc finger protein)	1.33	0.0347
SPEM1	spermatid maturation 1	1.33	0.0255
GPR15	G protein-coupled receptor 15	1.33	0.0415
SPACA5	sperm acrosome associated 5	1.33	0.0321

ANGPTL7	angiopoietin like 7	1.33	0.0272
ZNF561	zinc finger protein 561	1.33	0.0238
YLPM1	YLP motif containing 1	1.33	0.0342
ANKRD23	ankyrin repeat domain 23	1.33	0.0474
TMEM145	transmembrane protein 145	1.33	0.0239
PAQR6	progestin and adipoQ receptor family member VI	1.33	0.0477
LYG1	lysozyme G-like 1	1.32	0.0309
PRSS53	protease, serine 53	1.32	0.0262
ACTRT2	actin-related protein T2	1.32	0.0371
MLANA	melan-A	1.32	0.0259
HOXD8	homeobox D8	1.32	0.0342
ZSCAN25	zinc finger and SCAN domain containing 25	1.32	0.0265
EID3	EP300 interacting inhibitor of differentiation 3	1.32	0.0339
SPANXA2	SPANX family, member A2	1.32	0.0428
ADGRG6	adhesion G protein-coupled receptor G6	1.32	0.0275
GM2A	GM2 ganglioside activator	1.32	0.0408
TUBA4B	tubulin, alpha 4b	1.32	0.0337
CDC42BPG	CDC42 binding protein kinase gamma (DMPK-like)	1.32	0.024
TRPM2	transient receptor potential cation channel, subfamily M, member 2	1.32	0.0454
LRRC27	leucine rich repeat containing 27	1.32	0.0408
FANCC	Fanconi anemia complementation group C	1.32	0.0294
VWA5B1	von Willebrand factor A domain containing 5B1	1.32	0.027
GPR137C	G protein-coupled receptor 137C	1.32	0.0253
SMIM24	small integral membrane protein 24	1.32	0.0447
TSSK1B	testis-specific serine kinase 1B	1.32	0.0473
OR7A17	olfactory receptor, family 7, subfamily A, member 17	1.32	0.0315
CELA2A	chymotrypsin-like elastase family, member 2A	1.32	0.0382
TNFSF12-	TNFSF12-TNFSF13 readthrough	1.32	0.0257
PPARA	peroxisome proliferator-activated receptor alpha	1.32	0.0374
KRTAP9-1	keratin associated protein 9-1	1.32	0.0261
MAPT	microtubule associated protein tau	1.32	0.038
GDF2	growth differentiation factor 2	1.32	0.0353
EPHX1	epoxide hydrolase 1, microsomal (xenobiotic)	1.32	0.0411
ZNF358	zinc finger protein 358	1.32	0.0233
RINL	Ras and Rab interactor like	1.32	0.0298
ZNF324B	zinc finger protein 324B	1.32	0.0289
PLB1	phospholipase B1	1.32	0.0495
SCNN1D	sodium channel, non voltage gated 1 delta subunit	1.32	0.0271
PRAMEF2	PRAME family member 2	1.32	0.0444
EXOC3L4	exocyst complex component 3-like 4	1.32	0.0429
ADRA2A	adrenoceptor alpha 2A	1.32	0.0312
SCPEP1	serine carboxypeptidase 1	1.32	0.0309
TMEM100	transmembrane protein 100	1.32	0.0298
CD207	CD207 molecule, langerin	1.32	0.0313
UVSSA	UV stimulated scaffold protein A	1.32	0.0309
AIMP1	aminoacyl tRNA synthetase complex-interacting multifunctional protein 1	1.32	0.0316
GJC2	gap junction protein gamma 2	1.32	0.0283
ZNF530	zinc finger protein 530	1.32	0.0324
LACTBL1	lactamase, beta-like 1	1.32	0.0431
LMNTD2	lamin tail domain containing 2	1.32	0.0378
KLHL15	kelch-like family member 15	1.32	0.044
SLIT1	slit guidance ligand 1	1.32	0.0414
C11orf95	chromosome 11 open reading frame 95	1.32	0.0389
C10orf82	chromosome 10 open reading frame 82	1.32	0.0288
KNCN	kinocilin	1.32	0.0407
IL15RA	interleukin 15 receptor, alpha	1.32	0.0342
SLC22A11	solute carrier family 22 (organic anion/urate transporter), member 11	1.32	0.0446
CYTH2	cytohesin 2	1.32	0.0388
ZNF546	zinc finger protein 546	1.32	0.033

B4GALNT2	beta-1,4-N-acetyl-galactosaminyl transferase 2	1.31	0.042
FAM102B	family with sequence similarity 102, member B	1.31	0.0336
B3GALT5	UDP-Gal:betaGlcNAc beta 1,3-galactosyltransferase 5	1.31	0.0332
TSC22D4	TSC22 domain family, member 4	1.31	0.0279
MARS2	methionyl-tRNA synthetase 2, mitochondrial	1.31	0.0327
C9orf50	chromosome 9 open reading frame 50	1.31	0.0351
G6PD	glucose-6-phosphate dehydrogenase	1.31	0.0353
DUSP5	dual specificity phosphatase 5	1.31	0.0325
GLE1	GLE1 RNA export mediator	1.31	0.0488
HAL	histidine ammonia-lyase	1.31	0.0413
LGALS4	lectin, galactoside-binding, soluble, 4	1.31	0.0483
KRT28	keratin 28, type I	1.31	0.0327
GRID1	glutamate receptor, ionotropic, delta 1	1.31	0.0341
NLRX1	NLR family member X1	1.31	0.043
CLDN23	claudin 23	1.31	0.0407
TMEM239	transmembrane protein 239	1.31	0.045
THRA	thyroid hormone receptor, alpha	1.31	0.0489
THAP8	THAP domain containing 8	1.31	0.0458
SH3GL2	SH3-domain GRB2-like 2	1.31	0.0383
RHBDL1	rhomboid, veinlet-like 1 (Drosophila)	1.31	0.0478
PRAM1	PML-RARA regulated adaptor molecule 1	1.31	0.0349
CDC42EP4	CDC42 effector protein (Rho GTPase binding) 4	1.31	0.0301
USP7	ubiquitin specific peptidase 7 (herpes virus-associated)	1.31	0.0485
TMPRSS11D	transmembrane protease, serine 11D	1.31	0.0358
BHLHA15	basic helix-loop-helix family, member a15	1.31	0.0469
ZNF32	zinc finger protein 32	1.31	0.0432
FUT4	fucosyltransferase 4 (alpha (1,3) fucosyltransferase, myeloid-specific)	1.31	0.0357
GYS2	glycogen synthase 2 (liver)	1.31	0.0295
BCKDHB	branched chain keto acid dehydrogenase E1, beta polypeptide	1.31	0.0466
GSDMB	gasdermin B	1.31	0.0366
CSAG1	chondrosarcoma associated gene 1	1.31	0.0398
STXBP6	syntaxin binding protein 6 (amisyn)	1.31	0.0412
RWDD2A	RWD domain containing 2A	1.31	0.0448
DMRT1	doublesex and mab-3 related transcription factor 1	1.31	0.0306
PDLIM7	PDZ and LIM domain 7 (enigma)	1.31	0.0464
CCHCR1	coiled-coil alpha-helical rod protein 1	1.31	0.0364
BCL2L2-	BCL2L2-PABPN1 readthrough	1.31	0.0466
IFNA4	interferon, alpha 4	1.31	0.0499
LGI4	leucine-rich repeat LGI family, member 4	1.31	0.0346
HSD11B1L	hydroxysteroid (11-beta) dehydrogenase 1-like	1.31	0.0481
LZTS3	leucine zipper, putative tumor suppressor family member 3	1.31	0.047
MPP7	membrane protein, palmitoylated 7	1.31	0.0394
FNDC9	fibronectin type III domain containing 9	1.31	0.0366
ROM1	retinal outer segment membrane protein 1	1.31	0.0353
SPAG11A	sperm associated antigen 11A	1.31	0.039
MVP	major vault protein	1.31	0.046
SEC31B	SEC31 homolog B, COPII coat complex component	1.30	0.0306
ZFP82	ZFP82 zinc finger protein	1.30	0.0456
FUT11	fucosyltransferase 11 (alpha (1,3) fucosyltransferase)	1.30	0.037
RNF213	ring finger protein 213	1.30	0.0407
SLC23A3	solute carrier family 23, member 3	1.30	0.0365
PTPN18	protein tyrosine phosphatase, non-receptor type 18 (brain-derived)	1.30	0.04
GABPB2	GA binding protein transcription factor, beta subunit 2	1.30	0.0305
EPN1	epsin 1	1.30	0.0352
ART5	ADP-ribosyltransferase 5	1.30	0.0412
SERP2	stress-associated endoplasmic reticulum protein family member 2	1.30	0.0429
ADM	adrenomedullin	1.30	0.0386
CTRB1	chymotrypsinogen B1	1.30	0.049
MROH5	maestro heat-like repeat family member 5	1.30	0.0336

UBTD1	ubiquitin domain containing 1	1.30	0.0429
B3GALNT2	beta-1,3-N-acetylgalactosaminyltransferase 2	1.30	0.0489
CD19	CD19 molecule	1.30	0.0314
NFIA	nuclear factor I/A	1.30	0.0424
MFSD9	major facilitator superfamily domain containing 9	1.30	0.0423
BPIFB1	BPI fold containing family B, member 1	1.30	0.0468
KIF25	kinesin family member 25	1.30	0.0437
ZNF619	zinc finger protein 619	1.30	0.0458
ARID3B	AT rich interactive domain 3B (BRIGHT-like)	1.30	0.0316
SLC52A1	solute carrier family 52 (riboflavin transporter), member 1	1.30	0.0371
KIF2C	Jeck2013 ALT_DONOR, coding, INTERNAL, intronic best transcript	1.30	0.0342
PLA2G4D	phospholipase A2, group IVD (cytosolic)	1.30	0.0439
DMRTB1	DMRT-like family B with proline-rich C-terminal, 1	1.30	0.042
TPD52L3	tumor protein D52-like 3	1.30	0.0385
LAT2	linker for activation of T-cells family member 2	1.30	0.0416
MEN1	Jeck2013 ANTISENSE, CDS, coding, INTERNAL, OVCODE, OVEXON best	1.30	0.0497
OR4M2	Homo sapiens olfactory receptor, family 4, subfamily M, member 2 (OR4M2),	1.30	0.0364
LITAF	lipopolysaccharide-induced TNF factor	1.30	0.0439
LRRN4	leucine rich repeat neuronal 4	1.30	0.0387
FKBP9	FK506 binding protein 9	1.30	0.0434
NOTCH4	notch 4	1.30	0.0425
RBFOX1	Memczak2013 ALT_ACCEPTOR, ALT_DONOR, coding, INTERNAL, intronic	1.30	0.0445
ST6GALNAC6	ST6 (alpha-N-acetyl-neuraminyl-2,3-beta-galactosyl-1,3)-N-	1.30	0.0477
CLDN4	claudin 4	1.30	0.0426
RASSF4	Ras association (RalGDS/AF-6) domain family member 4	1.30	0.041
CFAP52	cilia and flagella associated protein 52	1.30	0.0346
TTLL8	tubulin tyrosine ligase-like family member 8	1.30	0.0432
BRINP2	bone morphogenetic protein/retinoic acid inducible neural-specific 2	1.30	0.0394
GDAP1L1	ganglioside induced differentiation associated protein 1-like 1	1.30	0.0427
C19orf38	chromosome 19 open reading frame 38	1.30	0.0376
RIC3	RIC3 acetylcholine receptor chaperone	1.29	0.0436
IL9R	interleukin 9 receptor	1.29	0.0457
WDR97	WD repeat domain 97	1.29	0.0422
SEL1L	sel-1 suppressor of lin-12-like (C. elegans)	1.29	0.0427
ABO	ABO blood group (transferase A, alpha 1-3-N-acetylgalactosaminyltransferase;	1.29	0.0465
RAPSN	receptor-associated protein of the synapse	1.29	0.0408
DMRT3	doublesex and mab-3 related transcription factor 3	1.29	0.0487
OR2V1	olfactory receptor, family 2, subfamily V, member 1	1.29	0.0471
HR	hair growth associated	1.29	0.0479
GEM	GTP binding protein overexpressed in skeletal muscle	1.29	0.049
GP2	glycoprotein 2 (zymogen granule membrane)	1.29	0.0426
PPP1R15A	protein phosphatase 1, regulatory subunit 15A	1.29	0.0462
ARMCX2	armadillo repeat containing, X-linked 2	1.29	0.0384
NPRL2	NPR2-like, GATOR1 complex subunit	1.29	0.0393
NCAN	neurocan	1.29	0.0443
NR1H3	nuclear receptor subfamily 1, group H, member 3	1.29	0.0474
SFTPA1	surfactant protein A1	1.29	0.038
KCNA7	potassium channel, voltage gated shaker related subfamily A, member 7	1.29	0.0449
FTL	ferritin, light polypeptide	1.29	0.0419
LRRC8C	leucine rich repeat containing 8 family, member C	1.29	0.0429
NAIF1	nuclear apoptosis inducing factor 1	1.29	0.0414
AK8	adenylate kinase 8	1.29	0.0414
GGT1	gamma-glutamyltransferase 1	1.29	0.0466
ITPKA	inositol-trisphosphate 3-kinase A	1.29	0.0469
FAM199X	family with sequence similarity 199, X-linked	1.29	0.0395
CD1B	CD1b molecule	1.29	0.0433
LAMTOR1	late endosomal/lysosomal adaptor, MAPK and MTOR activator 1	1.29	0.0445
NIPA1	non imprinted in Prader-Willi/Angelman syndrome 1	1.29	0.0488
CACNA1H	calcium channel, voltage-dependent, T type, alpha 1H subunit	1.29	0.0471

MEGF11	multiple EGF-like-domains 11	1.29	0.0452
TRPM8	transient receptor potential cation channel, subfamily M, member 8	1.29	0.0453
AFAP1L1	actin filament associated protein 1-like 1	1.29	0.047
OR10G7	olfactory receptor, family 10, subfamily G, member 7	1.29	0.0485
FLI1	Fli-1 proto-oncogene, ETS transcription factor	1.28	0.0423
GYPE	glycophorin E (MNS blood group)	1.28	0.0436
SLC6A15	solute carrier family 6 (neutral amino acid transporter), member 15	1.28	0.0448
ITIH1	inter-alpha-trypsin inhibitor heavy chain 1	1.28	0.0494
SLC2A4RG	SLC2A4 regulator	1.28	0.0428
SLC26A6	solute carrier family 26 (anion exchanger), member 6	1.28	0.0481
CTNS	cystinosin, lysosomal cystine transporter	1.28	0.0424
IFNA10	interferon, alpha 10	1.28	0.0462
CGA	glycoprotein hormones, alpha polypeptide	1.28	0.0475
ST6GALNAC2	ST6 (alpha-N-acetyl-neuraminyl-2,3-beta-galactosyl-1,3)-N-	1.28	0.0495
FAM78B	family with sequence similarity 78, member B	1.28	0.0435
MBD3L3	methyl-CpG binding domain protein 3-like 3	1.28	0.0491
PIK3CG	Memczak2013 ANTISENSE, CDS, coding, INTERNAL best transcript	1.28	0.0442
SCO1	SCO1 cytochrome c oxidase assembly protein	1.28	0.0475
CLDN16	claudin 16	1.28	0.0434
PRAMEF7	PRAME family member 7	1.28	0.0489
ZNF341	zinc finger protein 341	1.28	0.0448
NGFR	nerve growth factor receptor	1.28	0.0483
ELFN2	extracellular leucine-rich repeat and fibronectin type III domain containing 2	1.28	0.0496
DGCR8	DGCR8 microprocessor complex subunit	1.28	0.0437
GJB7	gap junction protein beta 7	1.28	0.0447
BCAS1	breast carcinoma amplified sequence 1	1.28	0.0474
EML5	echinoderm microtubule associated protein like 5	1.27	0.0487
FBXO36	Transcript Identified by AceView, Entrez Gene ID(s) 130888	1.27	0.0444
GPX8	glutathione peroxidase 8 (putative)	1.27	0.0478

Table S6. IDMF-decreased genes (P < 0.05 with FC < 0.80). The table lists 903 IDMF-decreased genes ranked by estimated fold-change (FC) (IDMF/CTL). The gene symbol and description are shown. The raw p-value is listed in the final column (generalized least square linear models with moderated t-statistics; *n* = 3 samples per group; *FDR < 0.10).

Symbol	Description	FC	P-value
ACTR3	ARP3 actin-related protein 3 homolog (yeast)	0.28	2.22e-07*
KPNB1	karyopherin (importin) beta 1	0.30	1.65e-05*
RAP1B	RAP1B, member of RAS oncogene family	0.32	2.58e-06*
TNFAIP3	tumor necrosis factor, alpha-induced protein 3	0.34	7.89e-07*
COPA	coatomer protein complex subunit alpha	0.44	2.03e-05*
WDR41	WD repeat domain 41	0.44	0.00075
SPPL2A	signal peptide peptidase like 2A	0.45	0.00198
TP53INP1	tumor protein p53 inducible nuclear protein 1	0.46	0.000133*
TMEM138	transmembrane protein 138	0.49	0.000517
METTL9	methyltransferase like 9	0.49	0.000113*
MID1	midline 1	0.50	0.000269
PSMB1	proteasome subunit beta 1	0.50	0.00276
EFEMP1	EGF containing fibulin-like extracellular matrix protein 1	0.51	9.98e-05*
SMURF2	SMAD specific E3 ubiquitin protein ligase 2	0.52	0.00369
CSF1	colony stimulating factor 1 (macrophage)	0.52	3.52e-05*
ABCE1	ATP binding cassette subfamily E member 1	0.52	0.000479
ARFGEF1	ADP-ribosylation factor guanine nucleotide-exchange factor 1 (brefeldin A-	0.52	8.62e-05*
PARL	presenilin associated, rhomboid-like	0.53	0.00536
EXOC1	exocyst complex component 1	0.53	0.00192
SLC30A9	solute carrier family 30 (zinc transporter), member 9	0.53	0.000355
MAPK8	mitogen-activated protein kinase 8	0.53	0.00477
NABP1	nucleic acid binding protein 1	0.53	9.89e-05*
PSMD12	proteasome 26S subunit, non-ATPase 12	0.53	0.000864
UCHL3	ubiquitin C-terminal hydrolase L3	0.53	0.00942
MRPS28	mitochondrial ribosomal protein S28	0.54	0.00691
PSMB5	proteasome subunit beta 5	0.54	0.000305
PRKCI	protein kinase C, iota	0.54	0.00562
RAD50	RAD50 homolog, double strand break repair protein	0.54	0.000363
PACSIN2	protein kinase C and casein kinase substrate in neurons 2	0.54	9.17e-05*
DICER1	dicer 1, ribonuclease type III	0.54	0.000986
SNAP23	synaptosome associated protein 23kDa	0.54	0.00169
TERF1	telomeric repeat binding factor (NIMA-interacting) 1	0.55	0.00515
UBR5	ubiquitin protein ligase E3 component n-recognin 5	0.55	0.000334
GTF3C6	general transcription factor IIIC subunit 6	0.55	0.0313
DCAF16	DDB1 and CUL4 associated factor 16	0.55	0.000159*
WDR61	WD repeat domain 61	0.55	0.00064
CORO1C	coronin, actin binding protein, 1C	0.55	0.00187
PDZD8	PDZ domain containing 8	0.55	0.00126
NUP50	nucleoporin 50kDa	0.55	0.00359
ACTC1	actin, alpha, cardiac muscle 1	0.55	0.000128*
ANKRD13A	ankyrin repeat domain 13A	0.55	0.00663
KCTD10	potassium channel tetramerization domain containing 10	0.55	0.00519
SFT2D1	SFT2 domain containing 1	0.55	0.00619
HIGD1A	HIG1 hypoxia inducible domain family, member 1A	0.55	0.0114
PGRMC2	progesterone receptor membrane component 2	0.56	0.00164
ENY2	enhancer of yellow 2 homolog (Drosophila)	0.56	0.00346
SYNRG	synergin, gamma	0.56	0.00317
IFNAR2	interferon (alpha, beta and omega) receptor 2	0.56	0.000452
GSPT1	G1 to S phase transition 1	0.56	0.0109
APBB2	amyloid beta (A4) precursor protein-binding, family B, member 2	0.56	0.00233
ITGAE	integrin alpha E	0.56	0.000965
CNDP2	CNDP dipeptidase 2 (metallopeptidase M20 family)	0.56	0.00143
TAPT1	transmembrane anterior posterior transformation 1	0.56	0.0038
MED6	mediator complex subunit 6	0.56	0.000154*

TANK	TRAF family member-associated NFKB activator	0.57	0.016
LNPEP	leucyl/cystinyl aminopeptidase	0.57	0.00662
NDUFB5	NADH dehydrogenase (ubiquinone) 1 beta subcomplex, 5, 16kDa	0.57	0.0119
GBP1	guanylate binding protein 1, interferon-inducible	0.57	0.00133
CPEB4	cytoplasmic polyadenylation element binding protein 4	0.57	0.000303
NTM	neurotrimin	0.57	0.0134
DBNL	drebrin-like	0.57	0.000746
ARV1	ARV1 homolog, fatty acid homeostasis modulator	0.57	0.00157
GTF2H3	general transcription factor IIH subunit 3	0.57	0.00431
ANK3	ankyrin 3, node of Ranvier (ankyrin G)	0.57	0.00147
RCN2	reticulocalbin 2, EF-hand calcium binding domain	0.57	0.00566
LTBP1	latent transforming growth factor beta binding protein 1	0.57	0.00542
MRPL46	mitochondrial ribosomal protein L46	0.57	0.00523
APLP2	amyloid beta (A4) precursor-like protein 2	0.57	0.00527
MFSD14B	major facilitator superfamily domain containing 14B	0.58	0.00286
CLK1	CDC like kinase 1	0.58	0.0256
F3	coagulation factor III (thromboplastin, tissue factor)	0.58	0.000108*
PTP4A1	protein tyrosine phosphatase type IVA, member 1	0.58	0.0018
TIMM9	translocase of inner mitochondrial membrane 9 homolog (yeast)	0.58	0.0013
MED20	mediator complex subunit 20	0.58	0.000308
LUM	lumican	0.58	0.00367
KCTD4	potassium channel tetramerization domain containing 4	0.58	0.00331
DUSP11	dual specificity phosphatase 11	0.58	0.00625
TWF1	twinfilin actin binding protein 1	0.58	0.000796
ENC1	ectodermal-neural cortex 1 (with BTB domain)	0.58	0.000268
NEDD4L	neural precursor cell expressed, developmentally down-regulated 4-like, E3	0.58	0.0066
AMD1	adenosylmethionine decarboxylase 1	0.58	0.00508
EPB41L3	erythrocyte membrane protein band 4.1-like 3	0.58	0.000336
SNRPA1	small nuclear ribonucleoprotein polypeptide A	0.58	0.0306
NBAS	neuroblastoma amplified sequence	0.58	0.00194
MDH1	malate dehydrogenase 1	0.58	0.000985
EFCAB11	EF-hand calcium binding domain 11	0.58	0.0314
KBTBD2	kelch repeat and BTB (POZ) domain containing 2	0.58	0.00645
SNX5	sorting nexin 5	0.59	0.00888
ZBTB38	zinc finger and BTB domain containing 38	0.59	0.0235
GRSF1	G-rich RNA sequence binding factor 1	0.59	0.00597
METTL23	methyltransferase like 23	0.59	0.00464
MTR	5-methyltetrahydrofolate-homocysteine methyltransferase	0.59	0.000263
MAGI1	membrane associated guanylate kinase, WW and PDZ domain containing 1	0.59	0.00381
ANKRD1	ankyrin repeat domain 1 (cardiac muscle)	0.59	0.00192
PPP2R5C	protein phosphatase 2, regulatory subunit B, gamma	0.59	0.0112
HNRNPA2B1	heterogeneous nuclear ribonucleoprotein A2/B1	0.59	0.0119
EBAG9	estrogen receptor binding site associated, antigen, 9	0.59	0.00335
ZNF776	zinc finger protein 776	0.59	0.00225
FARP2	FERM, ARH/RhoGEF and pleckstrin domain protein 2	0.59	0.00359
TARDBP	TAR DNA binding protein	0.59	0.0037
IFT46	intraflagellar transport 46	0.59	0.0051
P3H2	prolyl 3-hydroxylase 2	0.59	0.00128
COX4I1	cytochrome c oxidase subunit IV isoform 1	0.59	0.00326
PRKACB	protein kinase, cAMP-dependent, catalytic, beta	0.60	0.00459
TMEM59	transmembrane protein 59	0.60	0.0053
LSM5	LSM5 homolog, U6 small nuclear RNA and mRNA degradation associated	0.60	0.00887
PLOD2	procollagen-lysine, 2-oxoglutarate 5-dioxygenase 2	0.60	0.00241
NUDT5	nudix hydrolase 5	0.60	0.00088
BCCIP	BRCA2 and CDKN1A interacting protein	0.60	0.00504
SEC23IP	SEC23 interacting protein	0.60	0.0154
RPS27A	ribosomal protein S27a	0.60	0.00697
ANKRD50	ankyrin repeat domain 50	0.60	0.00175
CCNB1	cyclin B1	0.60	0.00581

SEC11A	SEC11 homolog A, signal peptidase complex subunit	0.60	0.00642
SF3A3	splicing factor 3a subunit 3	0.60	0.00565
ZNF148	zinc finger protein 148	0.60	0.00553
CETN3	centrin 3	0.60	0.0235
XRCC5	X-ray repair complementing defective repair in Chinese hamster cells 5 (double-	0.60	0.0116
PSMC5	proteasome 26S subunit, ATPase 5	0.60	0.0055
TOMM5	translocase of outer mitochondrial membrane 5 homolog (yeast)	0.60	0.0133
NDUFA8	NADH dehydrogenase (ubiquinone) 1 alpha subcomplex, 8, 19kDa	0.61	0.00451
ZSWIM7	zinc finger, SWIM-type containing 7	0.61	0.0055
PDGFC	platelet derived growth factor C	0.61	0.00171
GTF2H2	general transcription factor IIH subunit 2	0.61	0.00118
LYPLAL1	lysophospholipase-like 1	0.61	0.0161
NRDC	nardilysin convertase	0.61	0.0102
MELK	maternal embryonic leucine zipper kinase	0.61	0.00388
RASA1	RAS p21 protein activator (GTPase activating protein) 1	0.61	0.00103
WNT5A	wingless-type MMTV integration site family, member 5A	0.61	0.00107
USP25	ubiquitin specific peptidase 25	0.61	0.00808
VPS72	vacuolar protein sorting 72 homolog (S. cerevisiae)	0.61	0.000476
CAPRIN1	cell cycle associated protein 1	0.61	0.0261
TPRKB	TP53RK binding protein	0.61	0.00127
ATAD1	ATPase family, AAA domain containing 1	0.61	0.00366
ARL4A	ADP-ribosylation factor like GTPase 4A	0.61	0.00224
GORASP2	golgi reassembly stacking protein 2	0.61	0.0034
ARIH1	ariadne RBR E3 ubiquitin protein ligase 1	0.61	0.018
ANO10	anoctamin 10	0.61	0.00281
TM4SF1	transmembrane 4 L six family member 1	0.61	0.000588
TMEM45A	transmembrane protein 45A	0.62	0.0199
ARPC2	actin related protein 2/3 complex subunit 2	0.62	0.00122
ZZZ3	zinc finger, ZZ-type containing 3	0.62	0.00878
TM2D1	TM2 domain containing 1	0.62	0.00409
ANAPC7	anaphase promoting complex subunit 7	0.62	0.00366
AGO3	argonaute RISC catalytic component 3	0.62	0.00724
CBWD3	COBW domain containing 3	0.62	0.0212
NUP155	nucleoporin 155kDa	0.62	0.00194
FCHSD2	FCH and double SH3 domains 2	0.62	0.00604
NFKBIA	nuclear factor of kappa light polypeptide gene enhancer in B-cells inhibitor, alpha	0.62	0.00227
YTHDF2	YTH N(6)-methyladenosine RNA binding protein 2	0.62	0.00369
C18orf21	chromosome 18 open reading frame 21	0.62	0.00196
MLH1	mutL homolog 1	0.62	0.0311
UQCRHL	ubiquinol-cytochrome c reductase hinge protein like	0.62	0.00477
CLDN1	claudin 1	0.62	0.00144
COPZ1	coatomer protein complex subunit zeta 1	0.62	0.0117
MYL12A	myosin light chain 12A	0.62	0.0141
ZNF616	zinc finger protein 616	0.62	0.00194
PSMG1	proteasome (prosome, macropain) assembly chaperone 1	0.62	0.0299
OXCT1	3-oxoacid CoA-transferase 1	0.62	0.013
CBR4	carbonyl reductase 4	0.62	0.00762
SRI	sorcin	0.62	0.0048
PAPOLA	poly(A) polymerase alpha	0.62	0.00393
PCNA	proliferating cell nuclear antigen	0.62	0.00815
TMEM234	transmembrane protein 234	0.63	0.00423
TDRD3	tudor domain containing 3	0.63	0.00178
SENP6	SUMO1/sentrin specific peptidase 6	0.63	0.0159
NOX4	NADPH oxidase 4	0.63	0.00273
MTA3	metastasis associated 1 family member 3	0.63	0.00109
CFAP20	cilia and flagella associated protein 20	0.63	0.0016
PPP1R7	protein phosphatase 1, regulatory subunit 7	0.63	0.00719
LSM1	LSM1 homolog, mRNA degradation associated	0.63	0.0167
POLR2H	polymerase (RNA) II (DNA directed) polypeptide H	0.63	0.00246

CTNNA1	catenin (cadherin-associated protein), alpha 1	0.63	0.00387
SF3B1	splicing factor 3b, subunit 1, 155kDa	0.63	0.0194
OSTF1	osteoclast stimulating factor 1	0.63	0.0265
DEGS1	delta(4)-desaturase, sphingolipid 1	0.63	0.00632
PSMD9	proteasome 26S subunit, non-ATPase 9	0.63	0.00628
UCP2	uncoupling protein 2 (mitochondrial, proton carrier)	0.63	0.000996
EEF1D	eukaryotic translation elongation factor 1 delta (guanine nucleotide exchange	0.63	0.0499
PFDN4	prefoldin subunit 4	0.63	0.00294
CYP51A1	cytochrome P450, family 51, subfamily A, polypeptide 1	0.63	0.00354
NBPF9	neuroblastoma breakpoint family, member 9	0.63	0.00907
MRPS17	mitochondrial ribosomal protein S17	0.63	0.00692
UBLCP1	ubiquitin-like domain containing CTD phosphatase 1	0.63	0.0408
ADNP2	ADNP homeobox 2	0.63	0.0487
ATP2B1	ATPase, Ca++ transporting, plasma membrane 1	0.63	0.00115
BMPR2	bone morphogenetic protein receptor type II	0.63	0.00253
SEC14L1	SEC14-like lipid binding 1	0.63	0.0149
KLHDC2	kelch domain containing 2	0.63	0.0326
PSMG2	proteasome (prosome, macropain) assembly chaperone 2	0.63	0.000872
MFSD1	major facilitator superfamily domain containing 1	0.63	0.00793
BCL6	B-cell CLL/lymphoma 6	0.63	0.00103
ZNF433	zinc finger protein 433	0.63	0.00304
MAPRE1	microtubule-associated protein, RP/EB family, member 1	0.63	0.00906
PDSS1	prenyl (decaprenyl) diphosphate synthase, subunit 1	0.64	0.00275
XPR1	xenotropic and polytropic retrovirus receptor 1	0.64	0.00453
IKZF5	IKAROS family zinc finger 5	0.64	0.0269
NRG1	neuregulin 1	0.64	0.0057
DCAF6	DDB1 and CUL4 associated factor 6	0.64	0.0016
BROX	BRO1 domain and CAAX motif containing	0.64	0.0192
IFNAR1	interferon (alpha, beta and omega) receptor 1	0.64	0.00251
EIF2AK1	eukaryotic translation initiation factor 2-alpha kinase 1	0.64	0.0096
PSMD2	proteasome 26S subunit, non-ATPase 2	0.64	0.00248
SNRNP25	small nuclear ribonucleoprotein, U11/U12 25kDa subunit	0.64	0.00278
ERH	enhancer of rudimentary homolog (Drosophila)	0.64	0.0115
SPTLC2	serine palmitoyltransferase, long chain base subunit 2	0.64	0.00656
FAM229B	family with sequence similarity 229, member B	0.64	0.00343
VEGFA	vascular endothelial growth factor A	0.64	0.00229
CCNT2	cyclin T2	0.64	0.0178
TATDN1	TatD DNase domain containing 1	0.64	0.0154
BAG1	BCL2-associated athanogene	0.64	0.0457
PARP1	poly(ADP-ribose) polymerase 1	0.64	0.00294
PLAA	phospholipase A2-activating protein	0.64	0.038
MORN2	MORN repeat containing 2	0.64	0.0256
GDI1	GDP dissociation inhibitor 1	0.64	0.00157
GOT1	glutamic-oxaloacetic transaminase 1, soluble	0.64	0.00274
CCZ1	CCZ1 homolog, vacuolar protein trafficking and biogenesis associated	0.64	0.00396
FZD6	frizzled class receptor 6	0.65	0.00464
NDUFAB1	NADH dehydrogenase (ubiquinone) 1, alpha/beta subcomplex, 1, 8kDa	0.65	0.0177
CCDC82	coiled-coil domain containing 82	0.65	0.0159
COPS3	COP9 signalosome subunit 3	0.65	0.00904
CFLAR	CASP8 and FADD like apoptosis regulator	0.65	0.00277
GNPNAT1	glucosamine-phosphate N-acetyltransferase 1	0.65	0.0215
ITGAV	integrin alpha V	0.65	0.00818
CCNC	cyclin C	0.65	0.0131
RPF2	ribosome production factor 2 homolog	0.65	0.0385
TMEM50A	transmembrane protein 50A	0.65	0.003
PLCG1	phospholipase C, gamma 1	0.65	0.00742
RNASEH2B	ribonuclease H2, subunit B	0.65	0.0191
CDS2	CDP-diacylglycerol synthase 2	0.65	0.00511
AP3S1	adaptor-related protein complex 3, sigma 1 subunit	0.65	0.0164

CASD1	CAS1 domain containing 1	0.65	0.0161
IAH1	isoamyl acetate-hydrolyzing esterase 1 homolog	0.65	0.00888
MFAP5	microfibrillar associated protein 5	0.65	0.0175
CHEK1	checkpoint kinase 1	0.65	0.0218
KIAA0100	KIAA0100	0.65	0.00297
STRADB	STE20-related kinase adaptor beta	0.65	0.0156
SENP1	SUMO1/sentrin specific peptidase 1	0.65	0.0302
ADCY3	adenylate cyclase 3	0.65	0.0014
SUMO1	small ubiquitin-like modifier 1	0.65	0.0067
RAB23	RAB23, member RAS oncogene family	0.65	0.005
NXF1	nuclear RNA export factor 1	0.65	0.00303
RRM2B	ribonucleotide reductase M2 B (TP53 inducible)	0.65	0.00365
COG7	component of oligomeric golgi complex 7	0.66	0.00422
TMCC1	transmembrane and coiled-coil domain family 1	0.66	0.00531
PIEZO1	piezo-type mechanosensitive ion channel component 1	0.66	0.00226
EDIL3	EGF-like repeats and discoidin I-like domains 3	0.66	0.00652
ERLEC1	endoplasmic reticulum lectin 1	0.66	0.00832
TTC14	tetratricopeptide repeat domain 14	0.66	0.00269
LPCAT2	lysophosphatidylcholine acyltransferase 2	0.66	0.015
MSN	moesin	0.66	0.0104
SCRN1	secernin 1	0.66	0.00393
FGD6	FYVE, RhoGEF and PH domain containing 6	0.66	0.00274
DCAF5	DDB1 and CUL4 associated factor 5	0.66	0.0045
HERPUD1	homocysteine-inducible, endoplasmic reticulum stress-inducible, ubiquitin-like	0.66	0.0077
USP33	ubiquitin specific peptidase 33	0.66	0.0296
STIM2	stromal interaction molecule 2	0.66	0.00615
CASP4	caspase 4	0.66	0.00197
PTAR1	protein prenyltransferase alpha subunit repeat containing 1	0.66	0.0103
REST	RE1-silencing transcription factor	0.66	0.0157
STYXL1	serine/threonine/tyrosine interacting-like 1	0.66	0.0117
PAWR	PRKC, apoptosis, WT1, regulator	0.66	0.00383
VTI1B	vesicle transport through interaction with t-SNAREs 1B	0.66	0.00438
PMS2	PMS1 homolog 2, mismatch repair system component	0.66	0.0124
PRDX4	peroxiredoxin 4	0.66	0.0105
OSBPL10	oxysterol binding protein-like 10	0.66	0.00178
LIMS1	LIM and senescent cell antigen-like domains 1	0.66	0.00275
RTN4	reticulon 4	0.66	0.00573
ANXA5	annexin A5	0.66	0.0198
ERCC8	excision repair cross-complementation group 8	0.66	0.012
CWC27	CWC27 spliceosome-associated protein homolog	0.66	0.0123
IWS1	IWS1 homolog (S. cerevisiae)	0.66	0.0064
HACD3	3-hydroxyacyl-CoA dehydratase 3	0.66	0.0153
POLR2G	polymerase (RNA) II (DNA directed) polypeptide G	0.66	0.00372
PMPCA	peptidase (mitochondrial processing) alpha	0.66	0.00383
IQCJ	IQ motif containing J	0.66	0.0113
FMR1	fragile X mental retardation 1	0.66	0.0142
BTBD3	BTB (POZ) domain containing 3	0.67	0.00362
USP21	ubiquitin specific peptidase 21	0.67	0.00328
RSPRY1	ring finger and SPRY domain containing 1	0.67	0.00747
BPGM	2,3-bisphosphoglycerate mutase	0.67	0.0181
APPBP2	amyloid beta precursor protein (cytoplasmic tail) binding protein 2	0.67	0.0137
EXT1	exostosin glycosyltransferase 1	0.67	0.00298
KANK1	KN motif and ankyrin repeat domains 1	0.67	0.00369
UBR2	ubiquitin protein ligase E3 component n-recognin 2	0.67	0.00818
MRPL27	mitochondrial ribosomal protein L27	0.67	0.0112
OGFRL1	opioid growth factor receptor-like 1	0.67	0.00702
CHMP2B	charged multivesicular body protein 2B	0.67	0.0275
CALM2	calmodulin 2 (phosphorylase kinase, delta)	0.67	0.0248
STAT3	signal transducer and activator of transcription 3 (acute-phase response factor)	0.67	0.00513

MAP4K5	mitogen-activated protein kinase kinase kinase kinase 5	0.67	0.0194
GTPBP8	GTP-binding protein 8 (putative)	0.67	0.0348
PARP4	poly(ADP-ribose) polymerase family member 4	0.67	0.00551
CSTF3	cleavage stimulation factor, 3 pre-RNA, subunit 3	0.67	0.0459
STC1	stanniocalcin 1	0.67	0.0381
TMEM254	transmembrane protein 254	0.67	0.00406
VPS36	vacuolar protein sorting 36 homolog (S. cerevisiae)	0.67	0.00485
PAK1	p21 protein (Cdc42/Rac)-activated kinase 1	0.67	0.00633
RAB34	RAB34, member RAS oncogene family	0.67	0.0275
TAB2	TGF-beta activated kinase 1/MAP3K7 binding protein 2	0.67	0.00938
REXO2	RNA exonuclease 2	0.67	0.0279
SEC11C	SEC11 homolog C, signal peptidase complex subunit	0.67	0.0124
PFDN5	prefoldin subunit 5	0.67	0.00937
RTN4IP1	reticulon 4 interacting protein 1	0.67	0.004
PDP1	pyruvate dehyrogenase phosphatase catalytic subunit 1	0.67	0.00347
FAM122B	family with sequence similarity 122B	0.67	0.00331
TMEM147	transmembrane protein 147	0.67	0.00939
ANO4	anoctamin 4	0.67	0.00538
MIS18A	MIS18 kinetochore protein A	0.67	0.0133
GHITM	growth hormone inducible transmembrane protein	0.67	0.0107
ZNF639	zinc finger protein 639	0.67	0.00404
AP2B1	adaptor-related protein complex 2, beta 1 subunit	0.67	0.00588
MRPL14	mitochondrial ribosomal protein L14	0.68	0.0171
EOGT	EGF domain-specific O-linked N-acetylglucosamine (GlcNAc) transferase	0.68	0.012
TTC37	tetratricopeptide repeat domain 37	0.68	0.045
KDM2A	lysine (K)-specific demethylase 2A	0.68	0.011
SORT1	sortilin 1	0.68	0.0319
EXOC6B	exocyst complex component 6B	0.68	0.00511
RIOK1	RIO kinase 1	0.68	0.0259
CCDC90B	coiled-coil domain containing 90B	0.68	0.0423
WDR43	WD repeat domain 43	0.68	0.0134
PTPRJ	protein tyrosine phosphatase, receptor type, J	0.68	0.00656
CCNJ	cyclin J	0.68	0.00537
CCT3	chaperonin containing TCP1, subunit 3 (gamma)	0.68	0.00943
UPF2	UPF2 regulator of nonsense transcripts homolog (yeast)	0.68	0.00395
UGGT1	UDP-glucose glycoprotein glucosyltransferase 1	0.68	0.00437
MYLK	myosin light chain kinase	0.68	0.0188
VPS41	vacuolar protein sorting 41 homolog (S. cerevisiae)	0.68	0.00737
NOTCH2	notch 2	0.68	0.00605
DSEL	dermatan sulfate epimerase-like	0.68	0.0194
TXNL4B	thioredoxin-like 4B	0.68	0.0172
AZI2	5-azacytidine induced 2	0.68	0.0262
EEA1	early endosome antigen 1	0.68	0.0389
RGS2	regulator of G-protein signaling 2	0.68	0.00357
CAV2	caveolin 2	0.68	0.00411
FBXL20	F-box and leucine-rich repeat protein 20	0.68	0.0211
ADPRM	ADP-ribose/CDP-alcohol diphosphatase, manganese-dependent	0.68	0.00418
TIMM10	translocase of inner mitochondrial membrane 10 homolog (yeast)	0.68	0.00362
LAMP2	lysosomal-associated membrane protein 2	0.68	0.0111
MOCS2	molybdenum cofactor synthesis 2	0.68	0.0465
EML4	echinoderm microtubule associated protein like 4	0.68	0.00817
MGAT2	mannosyl (alpha-1,6-)-glycoprotein beta-1,2-N-acetylglucosaminyltransferase	0.68	0.00797
CASP6	caspase 6	0.68	0.0235
DIABLO	diablo, IAP-binding mitochondrial protein	0.68	0.0329
TRIM16	tripartite motif containing 16	0.68	0.00858
RECK	reversion-inducing-cysteine-rich protein with kazal motifs	0.68	0.00798
HNRNPU	heterogeneous nuclear ribonucleoprotein U (scaffold attachment factor A)	0.68	0.0169
TARBP1	TAR (HIV-1) RNA binding protein 1	0.68	0.00564
TMEM183A	transmembrane protein 183A	0.68	0.00595

TXNL4A	thioredoxin-like 4A	0.68	0.0377
SYNJ2BP-	SYNJ2BP-COX16 readthrough	0.68	0.0195
ZNF189	zinc finger protein 189	0.68	0.0128
LSM8	LSM8 homolog, U6 small nuclear RNA associated	0.68	0.0219
RNF26	ring finger protein 26	0.69	0.00469
BDH2	3-hydroxybutyrate dehydrogenase, type 2	0.69	0.0072
CMC1	C-x(9)-C motif containing 1	0.69	0.0315
GAB3	GRB2-associated binding protein 3	0.69	0.0131
EIF3E	eukaryotic translation initiation factor 3, subunit E	0.69	0.0113
NPIPB4	nuclear pore complex interacting protein family, member B4	0.69	0.0172
RCOR1	REST corepressor 1	0.69	0.0085
TSPAN13	tetraspanin 13	0.69	0.00722
DIXDC1	DIX domain containing 1	0.69	0.00529
RPL5	ribosomal protein L5	0.69	0.0187
C6orf62	chromosome 6 open reading frame 62	0.69	0.0148
MAMLD1	mastermind-like domain containing 1	0.69	0.00751
GALNT1	polypeptide N-acetylgalactosaminyltransferase 1	0.69	0.0111
ID1	inhibitor of DNA binding 1, dominant negative helix-loop-helix protein	0.69	0.0098
NT5DC3	5-nucleotidase domain containing 3	0.69	0.00445
SETD9	SET domain containing 9	0.69	0.00459
GLRX3	glutaredoxin 3	0.69	0.00904
EDN1	endothelin 1	0.69	0.0068
TP53BP2	tumor protein p53 binding protein 2	0.69	0.0055
PCYOX1	prenylcysteine oxidase 1	0.69	0.013
HSD17B10	hydroxysteroid (17-beta) dehydrogenase 10	0.69	0.00735
GLS	glutaminase	0.69	0.0102
TAF9	TAF9 RNA polymerase II, TATA box binding protein (TBP)-associated factor,	0.69	0.0151
BTN3A2	butyrophilin, subfamily 3, member A2	0.69	0.0245
GAS6	growth arrest-specific 6	0.69	0.0165
XPC	xeroderma pigmentosum, complementation group C	0.69	0.0173
MMAA	methylmalonic aciduria (cobalamin deficiency) cblA type	0.69	0.00483
TGM2	transglutaminase 2	0.69	0.00372
PTX3	pentraxin 3, long	0.69	0.00426
RNF128	ring finger protein 128, E3 ubiquitin protein ligase	0.69	0.0453
IRF1	interferon regulatory factor 1	0.69	0.0258
SAP18	Sin3A associated protein 18kDa	0.69	0.0375
DHX36	DEAH (Asp-Glu-Ala-His) box polypeptide 36	0.69	0.00875
EIF2AK4	eukaryotic translation initiation factor 2 alpha kinase 4	0.69	0.0418
COA3	cytochrome c oxidase assembly factor 3	0.69	0.0162
INAFM1	InaF-motif containing 1	0.69	0.024
SIPA1L1	signal-induced proliferation-associated 1 like 1	0.69	0.0131
ZBED5	zinc finger, BED-type containing 5	0.69	0.00724
NAE1	NEDD8 activating enzyme E1 subunit 1	0.69	0.00867
PPP3CB	protein phosphatase 3, catalytic subunit, beta isozyme	0.69	0.0247
SEMA5A	sema domain, seven thrombospondin repeats (type 1 and type 1-like),	0.69	0.00773
HIVEP2	human immunodeficiency virus type I enhancer binding protein 2	0.69	0.00709
SPCS3	signal peptidase complex subunit 3	0.69	0.0116
ACTR8	ARP8 actin-related protein 8 homolog (yeast)	0.69	0.00825
MTDH	metadherin	0.69	0.0446
MATR3	matrin 3	0.69	0.0426
CD24	CD24 molecule	0.69	0.0215
TXNDC5	thioredoxin domain containing 5 (endoplasmic reticulum)	0.69	0.0105
POMGNT1	protein O-linked mannose N-acetylglucosaminyltransferase 1 (beta 1,2-)	0.69	0.0172
MALT1	MALT1 paracaspase	0.69	0.0349
SRSF5	serine/arginine-rich splicing factor 5	0.69	0.00624
MFSD14A	major facilitator superfamily domain containing 14A	0.69	0.00644
DAP3	death associated protein 3	0.69	0.0194
CGGBP1	CGG triplet repeat binding protein 1	0.69	0.0353
AP3M2	adaptor-related protein complex 3, mu 2 subunit	0.69	0.0176

AZIN1	antizyme inhibitor 1	0.69	0.022
SLC35A3	solute carrier family 35 (UDP-N-acetylglucosamine (UDP-GlcNAc) transporter),	0.70	0.0078
MEDAG	mesenteric estrogen-dependent adipogenesis	0.70	0.00658
CCDC96	coiled-coil domain containing 96	0.70	0.00863
AASDHPPT	aminoadipate-semialdehyde dehydrogenase-phosphopantetheinyl transferase	0.70	0.0131
DNAJC13	DnaJ (Hsp40) homolog, subfamily C, member 13	0.70	0.0296
MYL12B	myosin light chain 12B	0.70	0.0417
LY96	lymphocyte antigen 96	0.70	0.00734
MYOF	myoferlin	0.70	0.0112
ICAM1	intercellular adhesion molecule 1	0.70	0.00804
LTA4H	leukotriene A4 hydrolase	0.70	0.0377
SECISBP2L	SECIS binding protein 2-like	0.70	0.0226
SUB1	SUB1 homolog, transcriptional regulator	0.70	0.0227
BIN3	bridging integrator 3	0.70	0.0123
BBX	bobby sox homolog (Drosophila)	0.70	0.0165
CALM1	calmodulin 1 (phosphorylase kinase, delta)	0.70	0.0246
ANAPC1	anaphase promoting complex subunit 1	0.70	0.0286
TGDS	TDP-glucose 4,6-dehydratase	0.70	0.0222
RAP1A	RAP1A, member of RAS oncogene family	0.70	0.0214
BAG5	BCL2-associated athanogene 5	0.70	0.0237
FBXO22	F-box protein 22	0.70	0.0317
RCN1	reticulocalbin 1, EF-hand calcium binding domain	0.70	0.00794
NFU1	NFU1 iron-sulfur cluster scaffold	0.70	0.0202
OGFOD1	2-oxoglutarate and iron-dependent oxygenase domain containing 1	0.70	0.0434
HGSNAT	heparan-alpha-glucosaminide N-acetyltransferase	0.70	0.00593
MCUR1	mitochondrial calcium uniporter regulator 1	0.70	0.0337
ZNF302	zinc finger protein 302	0.70	0.0124
AGO2	argonaute RISC catalytic component 2	0.70	0.00853
USP9X	ubiquitin specific peptidase 9, X-linked	0.70	0.0138
SP100	SP100 nuclear antigen	0.70	0.0252
SNRPB	small nuclear ribonucleoprotein polypeptides B and B1	0.70	0.0094
ATP11C	ATPase, class VI, type 11C	0.70	0.0363
ZNF28	zinc finger protein 28	0.70	0.00694
ZNF532	zinc finger protein 532	0.70	0.0146
VAPA	VAMP associated protein A	0.70	0.0109
GNAZ	guanine nucleotide binding protein (G protein), alpha z polypeptide	0.70	0.0405
DHX15	DEAH (Asp-Glu-Ala-His) box helicase 15	0.70	0.0491
FBXL4	F-box and leucine-rich repeat protein 4	0.70	0.0067
WSB1	WD repeat and SOCS box containing 1	0.70	0.0195
HIF1A	hypoxia inducible factor 1, alpha subunit (basic helix-loop-helix transcription	0.70	0.0179
C1orf21	chromosome 1 open reading frame 21	0.70	0.0293
FYN	FYN proto-oncogene, Src family tyrosine kinase	0.70	0.00669
ZBTB8OS	zinc finger and BTB domain containing 8 opposite strand	0.70	0.043
UTP23	UTP23, small subunit (SSU) processome component, homolog (yeast)	0.70	0.0145
MTF2	metal response element binding transcription factor 2	0.70	0.0165
DGKI	diacylglycerol kinase, iota	0.70	0.0296
KLHL5	kelch-like family member 5	0.70	0.0247
GEMIN7	gem nuclear organelle associated protein 7	0.70	0.0301
DHX9	DEAH (Asp-Glu-Ala-His) box helicase 9	0.70	0.0337
IRAK4	interleukin 1 receptor associated kinase 4	0.70	0.04
CIR1	corepressor interacting with RBPJ, 1	0.70	0.0172
ERRFI1	ERBB receptor feedback inhibitor 1	0.70	0.0146
CDC42BPB	CDC42 binding protein kinase beta (DMPK-like)	0.70	0.017
RBX1	ring-box 1, E3 ubiquitin protein ligase	0.70	0.0238
TRMT112	tRNA methyltransferase 11-2 homolog (S. cerevisiae)	0.70	0.00708
WBP2	WW domain binding protein 2	0.70	0.0257
HIVEP1	human immunodeficiency virus type I enhancer binding protein 1	0.71	0.0145
IL13RA1	interleukin 13 receptor, alpha 1	0.71	0.0101
EID1	EP300 interacting inhibitor of differentiation 1	0.71	0.0312

RAD54L2	RAD54-like 2 (S. cerevisiae)	0.71	0.0107
KCNH1	potassium channel, voltage gated eag related subfamily H, member 1	0.71	0.00787
USP15	ubiquitin specific peptidase 15	0.71	0.0347
STK17A	serine/threonine kinase 17a	0.71	0.033
NCKAP1	NCK-associated protein 1	0.71	0.0177
PGAM1	Homo sapiens phosphoglycerate mutase 1 (brain), mRNA (cDNA clone	0.71	0.0129
ZNF26	zinc finger protein 26	0.71	0.0115
MBTPS1	membrane bound transcription factor peptidase, site 1	0.71	0.00945
OCRL	oculocerebrorenal syndrome of Lowe	0.71	0.0103
NT5DC2	5-nucleotidase domain containing 2	0.71	0.0143
PGK1	phosphoglycerate kinase 1	0.71	0.00833
NTAN1	N-terminal asparagine amidase	0.71	0.0111
TMEM8B	transmembrane protein 8B	0.71	0.0192
CCNK	cyclin K	0.71	0.0139
CA5B	carbonic anhydrase VB, mitochondrial	0.71	0.028
B4GALT3	UDP-Gal:betaGlcNAc beta 1,4- galactosyltransferase, polypeptide 3	0.71	0.0143
PSMD11	proteasome 26S subunit, non-ATPase 11	0.71	0.0254
TBRG1	transforming growth factor beta regulator 1	0.71	0.00925
SP110	SP110 nuclear body protein	0.71	0.0199
CBWD2	COBW domain containing 2	0.71	0.0441
NDUFS5	NADH dehydrogenase (ubiquinone) Fe-S protein 5, 15kDa (NADH-coenzyme Q	0.71	0.0112
ACYP1	acylphosphatase 1, erythrocyte (common) type	0.71	0.0324
ITFG1	integrin alpha FG-GAP repeat containing 1	0.71	0.0127
RAB7A	RAB7A, member RAS oncogene family	0.71	0.0137
DDX3X	DEAD (Asp-Glu-Ala-Asp) box helicase 3, X-linked	0.71	0.01
PPP2CA	protein phosphatase 2, catalytic subunit, alpha isozyme	0.71	0.0319
AKT2	v-akt murine thymoma viral oncogene homolog 2	0.71	0.0114
POLR2C	polymerase (RNA) II (DNA directed) polypeptide C, 33kDa	0.71	0.0242
FUS	FUS RNA binding protein	0.71	0.0157
KIAA0586	KIAA0586	0.71	0.03
ATG9A	autophagy related 9A	0.71	0.00935
MYO5A	myosin VA	0.71	0.0141
TCTN3	tectonic family member 3	0.71	0.00893
JAG1	jagged 1	0.71	0.0263
TNNI3K	TNNI3 interacting kinase	0.71	0.0127
ANKRD12	ankyrin repeat domain 12	0.71	0.0328
MRPL20	mitochondrial ribosomal protein L20	0.71	0.0243
RHOT1	ras homolog family member T1	0.71	0.0253
PIP5K1A	phosphatidylinositol-4-phosphate 5-kinase, type I, alpha	0.71	0.0192
SGTB	small glutamine-rich tetratricopeptide repeat (TPR)-containing, beta	0.71	0.0276
WDR45	WD repeat domain 45	0.71	0.0191
DSN1	DSN1 homolog, MIS12 kinetochore complex component	0.71	0.0171
TPP2	tripeptidyl peptidase II	0.71	0.0245
S100A16	S100 calcium binding protein A16	0.71	0.0323
CXCL8	chemokine (C-X-C motif) ligand 8	0.71	0.0491
MKNK1	MAP kinase interacting serine/threonine kinase 1	0.71	0.0447
ZNF879	zinc finger protein 879	0.71	0.0106
CFAP36	cilia and flagella associated protein 36	0.71	0.0225
ADAMTS12	ADAM metallopeptidase with thrombospondin type 1 motif 12	0.71	0.0105
RBMS1	RNA binding motif, single stranded interacting protein 1	0.71	0.0415
CUL5	cullin 5	0.72	0.023
TRPC1	transient receptor potential cation channel, subfamily C, member 1	0.72	0.019
IBTK	inhibitor of Bruton agammaglobulinemia tyrosine kinase	0.72	0.0371
EIF6	eukaryotic translation initiation factor 6	0.72	0.00836
MSMO1	methylsterol monooxygenase 1	0.72	0.0189
AP3S2	adaptor-related protein complex 3, sigma 2 subunit	0.72	0.0275
ARL3	ADP-ribosylation factor like GTPase 3	0.72	0.0181
ARID2	AT rich interactive domain 2 (ARID, RFX-like)	0.72	0.00889
TCTN1	tectonic family member 1	0.72	0.00903

KRT18	keratin 18, type I	0.72	0.00962
MARVELD1	MARVEL domain containing 1	0.72	0.0136
SLC25A36	solute carrier family 25 (pyrimidine nucleotide carrier), member 36	0.72	0.0342
TOP2A	topoisomerase (DNA) II alpha	0.72	0.0158
SGPL1	sphingosine-1-phosphate lyase 1	0.72	0.0155
NR3C1	nuclear receptor subfamily 3, group C, member 1 (glucocorticoid receptor)	0.72	0.0129
IFT80	intraflagellar transport 80	0.72	0.0114
RAB8B	RAB8B, member RAS oncogene family	0.72	0.0127
DDX17	DEAD (Asp-Glu-Ala-Asp) box helicase 17	0.72	0.0122
PTBP2	polypyrimidine tract binding protein 2	0.72	0.025
FTO	fat mass and obesity associated	0.72	0.0199
MOSPD2	motile sperm domain containing 2	0.72	0.00981
NDUFV2	NADH dehydrogenase (ubiquinone) flavoprotein 2, 24kDa	0.72	0.0422
PARPBP	PARP1 binding protein	0.72	0.0131
CREBBP	CREB binding protein	0.72	0.0212
MOGS	mannosyl-oligosaccharide glucosidase	0.72	0.0111
SYNCRIP	synaptotagmin binding, cytoplasmic RNA interacting protein	0.72	0.0251
POLR2B	polymerase (RNA) II (DNA directed) polypeptide B, 140kDa	0.72	0.024
TNC	tenascin C	0.72	0.0114
FRMD4A	FERM domain containing 4A	0.72	0.0196
CTNNB1	catenin (cadherin-associated protein), beta 1	0.72	0.0184
CYB5A	cytochrome b5 type A (microsomal)	0.72	0.0267
PRPF40A	PRP40 pre-mRNA processing factor 40 homolog A	0.72	0.0462
SUDS3	SDS3 homolog, SIN3A corepressor complex component	0.72	0.0198
NRXN3	neurexin 3	0.72	0.0158
PPP1CA	protein phosphatase 1, catalytic subunit, alpha isozyme	0.72	0.0288
PGM3	phosphoglucomutase 3	0.72	0.0222
TYK2	tyrosine kinase 2	0.72	0.0109
CTBP2	C-terminal binding protein 2	0.72	0.0126
RNMT	RNA (guanine-7-) methyltransferase	0.72	0.0191
NGLY1	N-glycanase 1	0.72	0.0196
PWP1	PWP1 homolog, endonuclein	0.72	0.0234
MYDGF	myeloid-derived growth factor	0.72	0.0175
MTMR10	myotubularin related protein 10	0.72	0.0384
COPB2	coatomer protein complex subunit beta 2 (beta prime)	0.72	0.0145
TRAPPC3	trafficking protein particle complex 3	0.72	0.0153
SMARCAD1	SWI/SNF-related, matrix-associated actin-dependent regulator of chromatin,	0.72	0.0125
BDNF	brain-derived neurotrophic factor	0.72	0.0243
NME1	NME/NM23 nucleoside diphosphate kinase 1	0.72	0.0272
TJP1	tight junction protein 1	0.72	0.015
FDX1	ferredoxin 1	0.72	0.0145
PTPRD	protein tyrosine phosphatase, receptor type, D	0.72	0.0264
USP30	ubiquitin specific peptidase 30	0.72	0.0291
GLO1	glyoxalase I	0.72	0.0314
ACTN1	actinin, alpha 1	0.72	0.0236
ZFR	zinc finger RNA binding protein	0.72	0.0241
BHLHE40	basic helix-loop-helix family, member e40	0.72	0.0109
UBL3	ubiquitin-like 3	0.72	0.0321
EIF2B1	eukaryotic translation initiation factor 2B, subunit 1 alpha, 26kDa	0.72	0.0123
ACBD6	acyl-CoA binding domain containing 6	0.72	0.0315
TUBB2A	tubulin, beta 2A class IIa	0.72	0.0135
DNAJA1	DnaJ (Hsp40) homolog, subfamily A, member 1	0.72	0.0468
IMPACT	impact RWD domain protein	0.72	0.0167
MALSU1	mitochondrial assembly of ribosomal large subunit 1	0.72	0.0346
ARMC1	armadillo repeat containing 1	0.72	0.0249
C20orf194	chromosome 20 open reading frame 194	0.72	0.0456
TRIM9	tripartite motif containing 9	0.72	0.0125
NDUFB2	NADH dehydrogenase (ubiquinone) 1 beta subcomplex, 2, 8kDa	0.72	0.026
TRAPPC1	trafficking protein particle complex 1	0.72	0.0152

MCFD2	multiple coagulation factor deficiency 2	0.72	0.0289
CCDC80	coiled-coil domain containing 80	0.72	0.0303
YPEL2	yippee like 2	0.73	0.0241
FAM120AOS	family with sequence similarity 120A opposite strand	0.73	0.0123
UFC1	ubiquitin-fold modifier conjugating enzyme 1	0.73	0.034
GALNT2	polypeptide N-acetylgalactosaminyltransferase 2	0.73	0.0249
MTMR12	myotubularin related protein 12	0.73	0.0236
PLCB4	phospholipase C, beta 4	0.73	0.0399
SYT17	synaptotagmin XVII	0.73	0.016
GATAD1	GATA zinc finger domain containing 1	0.73	0.0299
MRPL44	mitochondrial ribosomal protein L44	0.73	0.0448
PTPMT1	protein tyrosine phosphatase, mitochondrial 1	0.73	0.0204
LANCL2	LanC lantibiotic synthetase component C-like 2 (bacterial)	0.73	0.0226
CCZ1B	CCZ1 homolog B, vacuolar protein trafficking and biogenesis associated	0.73	0.0172
GANAB	glucosidase, alpha; neutral AB	0.73	0.0138
PIK3C2A	phosphatidylinositol-4-phosphate 3-kinase, catalytic subunit type 2 alpha	0.73	0.0222
MASTL	microtubule associated serine/threonine kinase-like	0.73	0.0109
AAMDC	adipogenesis associated, Mth938 domain containing	0.73	0.0357
REV1	REV1, DNA directed polymerase	0.73	0.0223
AHCYL1	adenosylhomocysteinase like 1	0.73	0.0202
ZNF439	zinc finger protein 439	0.73	0.0115
AP2M1	adaptor-related protein complex 2, mu 1 subunit	0.73	0.0123
SPECC1	sperm antigen with calponin homology and coiled-coil domains 1	0.73	0.0416
GPBP1L1	GC-rich promoter binding protein 1-like 1	0.73	0.0312
RYK	receptor-like tyrosine kinase	0.73	0.0312
ANLN	anillin actin binding protein	0.73	0.0411
PLXDC2	plexin domain containing 2	0.73	0.0211
ATP6V1C1	ATPase, H+ transporting, lysosomal 42kDa, V1 subunit C1	0.73	0.0273
PCYT2	phosphate cytidylyltransferase 2, ethanolamine	0.73	0.0144
SKIL	SKI-like proto-oncogene	0.73	0.0131
PDE1C	phosphodiesterase 1C, calmodulin-dependent 70kDa	0.73	0.0172
DMD	dystrophin	0.73	0.0404
WEE1	WEE1 G2 checkpoint kinase	0.73	0.0194
TBC1D19	TBC1 domain family, member 19	0.73	0.0406
MRPS14	mitochondrial ribosomal protein S14	0.73	0.0143
ZNF644	zinc finger protein 644	0.73	0.0273
TRMT61B	tRNA methyltransferase 61B	0.73	0.0144
RABEPK	Rab9 effector protein with kelch motifs	0.73	0.0449
KHDRBS3	KH domain containing, RNA binding, signal transduction associated 3	0.73	0.0237
CCNH	cyclin H	0.73	0.0322
GPN2	GPN-loop GTPase 2	0.73	0.0142
ASIC1	acid sensing ion channel 1	0.73	0.0235
HDAC1	histone deacetylase 1	0.73	0.0153
GTF2H2C_2	GTF2H2 family member C, copy 2	0.73	0.0366
CHCHD1	coiled-coil-helix-coiled-coil-helix domain containing 1	0.73	0.0203
INSIG2	insulin induced gene 2	0.73	0.0184
SLC30A7	solute carrier family 30 (zinc transporter), member 7	0.73	0.0196
ZDHHC2	zinc finger, DHHC-type containing 2	0.73	0.0248
ZDHHC21	zinc finger, DHHC-type containing 21	0.73	0.019
EXOSC7	exosome component 7	0.73	0.0369
GPR107	G protein-coupled receptor 107	0.73	0.0164
MMADHC	methylmalonic aciduria (cobalamin deficiency) cblD type, with homocystinuria	0.73	0.0487
KIAA2026	KIAA2026	0.73	0.0214
CFI	complement factor I	0.73	0.0398
COX7A2	cytochrome c oxidase subunit VIIa polypeptide 2 (liver)	0.73	0.0204
CLTC	clathrin, heavy chain (Hc)	0.73	0.0379
TAF2	TAF2 RNA polymerase II, TATA box binding protein (TBP)-associated factor,	0.73	0.0412
TRPM7	transient receptor potential cation channel, subfamily M, member 7	0.73	0.0429
VAMP3	vesicle associated membrane protein 3	0.73	0.0359

RTF1	RTF1 homolog, Paf1/RNA polymerase II complex component	0.73	0.0394
TMED2	transmembrane p24 trafficking protein 2	0.73	0.029
FUOM	fucose mutarotase	0.73	0.0147
ZNF558	zinc finger protein 558	0.73	0.0247
GPR137B	G protein-coupled receptor 137B	0.73	0.0206
ZNF24	zinc finger protein 24	0.73	0.0232
NRAS	neuroblastoma RAS viral (v-ras) oncogene homolog	0.73	0.0292
FUCA1	fucosidase, alpha-L- 1, tissue	0.73	0.0393
TMED7-	TMED7-TICAM2 readthrough	0.73	0.037
AREL1	apoptosis resistant E3 ubiquitin protein ligase 1	0.73	0.0347
RAN	RAN, member RAS oncogene family	0.73	0.0364
DDX6	DEAD (Asp-Glu-Ala-Asp) box helicase 6	0.73	0.0309
ARHGEF28	Rho guanine nucleotide exchange factor 28	0.73	0.0483
NREP	neuronal regeneration related protein	0.73	0.0331
BMP2K	BMP2 inducible kinase	0.73	0.0335
FEZ1	fasciculation and elongation protein zeta 1	0.73	0.0356
MLC1	megalencephalic leukoencephalopathy with subcortical cysts 1	0.73	0.0326
RRP36	ribosomal RNA processing 36	0.74	0.0283
CREB3	cAMP responsive element binding protein 3	0.74	0.0202
GLT8D2	glycosyltransferase 8 domain containing 2	0.74	0.019
NUAK1	NUAK family, SNF1-like kinase, 1	0.74	0.0162
CDKN1C	cyclin-dependent kinase inhibitor 1C (p57, Kip2)	0.74	0.0233
PLK2	polo-like kinase 2	0.74	0.0342
DNMBP	dynamin binding protein	0.74	0.0342
OPHN1	oligophrenin 1	0.74	0.0199
SERINC1	serine incorporator 1	0.74	0.023
PDCD2L	programmed cell death 2-like	0.74	0.0393
NDUFA12	NADH dehydrogenase (ubiquinone) 1 alpha subcomplex, 12	0.74	0.0325
KCTD20	potassium channel tetramerization domain containing 20	0.74	0.0141
TIMM23	translocase of inner mitochondrial membrane 23 homolog (yeast)	0.74	0.0305
SCARB2	scavenger receptor class B, member 2	0.74	0.0269
RBFOX2	RNA binding protein, fox-1 homolog (C. elegans) 2	0.74	0.0168
ATIC	5-aminoimidazole-4-carboxamide ribonucleotide formyltransferase/IMP	0.74	0.0453
RPL39	ribosomal protein L39	0.74	0.0377
EIF4A2	eukaryotic translation initiation factor 4A2	0.74	0.0341
PEX12	peroxisomal biogenesis factor 12	0.74	0.0215
NSL1	NSL1, MIS12 kinetochore complex component	0.74	0.0162
ZNF548	zinc finger protein 548	0.74	0.0166
PGAM4	phosphoglycerate mutase family member 4	0.74	0.0452
RNF10	ring finger protein 10	0.74	0.0347
RAPH1	Ras association (RalGDS/AF-6) and pleckstrin homology domains 1	0.74	0.0191
KDM5B	lysine (K)-specific demethylase 5B	0.74	0.0326
MRPS27	mitochondrial ribosomal protein S27	0.74	0.0444
EXOC6	exocyst complex component 6	0.74	0.0254
PHF12	PHD finger protein 12	0.74	0.0322
DGUOK	deoxyguanosine kinase	0.74	0.0388
NAGK	N-acetylglucosamine kinase	0.74	0.0328
MMP1	matrix metallopeptidase 1	0.74	0.0422
GTF2E2	general transcription factor IIE subunit 2	0.74	0.0228
FKBP14	FK506 binding protein 14	0.74	0.0249
ESCO1	establishment of sister chromatid cohesion N-acetyltransferase 1	0.74	0.0241
ZDHHC7	zinc finger, DHHC-type containing 7	0.74	0.0317
UBXN8	UBX domain protein 8	0.74	0.0435
PHACTR4	phosphatase and actin regulator 4	0.74	0.0239
TOMM22	translocase of outer mitochondrial membrane 22 homolog (yeast)	0.74	0.0263
CNIH1	cornichon family AMPA receptor auxiliary protein 1	0.74	0.0479
SAMD9L	sterile alpha motif domain containing 9-like	0.74	0.0245
DDX58	DEAD (Asp-Glu-Ala-Asp) box polypeptide 58	0.74	0.0216
SPAG7	sperm associated antigen 7	0.74	0.0321

LIX1L	limb and CNS expressed 1 like	0.74	0.0307
COQ10B	coenzyme Q10B	0.74	0.0356
CHSY1	chondroitin sulfate synthase 1	0.74	0.0409
CHN1	chimerin 1	0.74	0.0355
MPV17	MpV17 mitochondrial inner membrane protein	0.74	0.019
BNC2	basonuclin 2	0.74	0.0212
SH2D4A	SH2 domain containing 4A	0.74	0.0388
FAM83G	family with sequence similarity 83, member G	0.75	0.0325
CHD2	chromodomain helicase DNA binding protein 2	0.75	0.022
TAF9B	TAF9B RNA polymerase II, TATA box binding protein (TBP)-associated factor,	0.75	0.024
MAGOH	mago homolog, exon junction complex core component	0.75	0.0429
FBN1	fibrillin 1	0.75	0.0356
RNPEP	arginyl aminopeptidase (aminopeptidase B)	0.75	0.0331
XRCC6	X-ray repair complementing defective repair in Chinese hamster cells 6	0.75	0.0257
HADHA	hydroxyacyl-CoA dehydrogenase/3-ketoacyl-CoA thiolase/enoyl-CoA hydratase	0.75	0.0427
HHIP	hedgehog interacting protein	0.75	0.0203
ALG8	ALG8, alpha-1,3-glucosyltransferase	0.75	0.0341
RPP30	ribonuclease P/MRP 30kDa subunit	0.75	0.0249
TSEN2	TSEN2 tRNA splicing endonuclease subunit	0.75	0.0353
TUBGCP2	tubulin, gamma complex associated protein 2	0.75	0.0295
EIF2B2	eukaryotic translation initiation factor 2B, subunit 2 beta, 39kDa	0.75	0.025
DMTF1	cyclin D binding myb-like transcription factor 1	0.75	0.0279
BPHL	biphenyl hydrolase-like (serine hydrolase)	0.75	0.0417
SLC16A9	solute carrier family 16, member 9	0.75	0.0223
UTP18	UTP18 small subunit (SSU) processome component	0.75	0.0375
ZYG11B	zyg-11 family member B, cell cycle regulator	0.75	0.0396
SPOPL	speckle-type POZ protein-like	0.75	0.04
TPX2	TPX2, microtubule-associated	0.75	0.0453
TAF12	TAF12 RNA polymerase II, TATA box binding protein (TBP)-associated factor,	0.75	0.0354
ZFHX4	zinc finger homeobox 4	0.75	0.0325
ARMC9	armadillo repeat containing 9	0.75	0.0404
FAF1	Fas (TNFRSF6) associated factor 1	0.75	0.0299
TUBB2B	tubulin, beta 2B class IIb	0.75	0.0475
ZCCHC9	zinc finger, CCHC domain containing 9	0.75	0.0277
STK32B	serine/threonine kinase 32B	0.75	0.0497
SMAD1	SMAD family member 1	0.75	0.0202
RAB21	RAB21, member RAS oncogene family	0.75	0.031
QSOX1	quiescin Q6 sulfhydryl oxidase 1	0.75	0.0231
TADA1	transcriptional adaptor 1	0.75	0.0413
DCTN2	dynactin 2 (p50)	0.75	0.0275
OTUB1	OTU deubiquitinase, ubiquitin aldehyde binding 1	0.75	0.0208
GALNT5	polypeptide N-acetylgalactosaminyltransferase 5	0.75	0.0447
MRPS18A	mitochondrial ribosomal protein S18A	0.75	0.0252
DENND4C	DENN/MADD domain containing 4C	0.75	0.04
LRP12	LDL receptor related protein 12	0.75	0.0305
GOLPH3	golgi phosphoprotein 3 (coat-protein)	0.75	0.0304
TXNIP	thioredoxin interacting protein	0.75	0.0312
TBC1D1	TBC1 (tre-2/USP6, BUB2, cdc16) domain family, member 1	0.75	0.0379
CDC23	cell division cycle 23	0.75	0.0381
GNPAT	glyceronephosphate O-acyltransferase	0.75	0.0493
GPR89A	G protein-coupled receptor 89A	0.75	0.0362
FBXO32	F-box protein 32	0.75	0.0264
GNAI3	guanine nucleotide binding protein (G protein), alpha inhibiting activity	0.75	0.0494
DERL1	derlin 1	0.75	0.0246
TMEM205	transmembrane protein 205	0.75	0.0321
AP1G1	adaptor-related protein complex 1, gamma 1 subunit	0.75	0.0299
UBE3A	ubiquitin protein ligase E3A	0.75	0.0316
ARPC4	actin related protein 2/3 complex subunit 4	0.75	0.0256
EFNB2	ephrin-B2	0.75	0.0315

ITGA2	integrin, alpha 2 (CD49B, alpha 2 subunit of VLA-2 receptor)	0.75	0.0334
GABRG3	gamma-aminobutyric acid (GABA) A receptor, gamma 3	0.75	0.0282
IDH3B	isocitrate dehydrogenase 3 (NAD+) beta	0.75	0.0321
PPRC1	peroxisome proliferator-activated receptor gamma, coactivator-related 1	0.75	0.0277
ACTR10	actin-related protein 10 homolog (S. cerevisiae)	0.75	0.0372
LRRC8B	leucine rich repeat containing 8 family, member B	0.75	0.034
CEMIP	cell migration inducing protein, hyaluronan binding	0.75	0.0241
IFNGR2	interferon gamma receptor 2 (interferon gamma transducer 1)	0.75	0.0263
TMEM168	transmembrane protein 168	0.76	0.0284
BIRC3	baculoviral IAP repeat containing 3	0.76	0.0253
NSFL1C	NSFL1 (p97) cofactor (p47)	0.76	0.0413
NDUFB8	NADH dehydrogenase (ubiquinone) 1 beta subcomplex, 8, 19kDa	0.76	0.0306
PITPNB	phosphatidylinositol transfer protein, beta	0.76	0.0417
MAP1B	microtubule associated protein 1B	0.76	0.0384
NAGA	N-acetylgalactosaminidase, alpha-	0.76	0.0267
ZNF280B	zinc finger protein 280B	0.76	0.0452
TGS1	trimethylguanosine synthase 1	0.76	0.0425
MAP3K3	mitogen-activated protein kinase kinase kinase 3	0.76	0.0285
FKTN	fukutin	0.76	0.0456
ARHGEF25	Rho guanine nucleotide exchange factor 25	0.76	0.0352
STK38	serine/threonine kinase 38	0.76	0.037
COMMD1	copper metabolism (Murr1) domain containing 1	0.76	0.0448
NCBP2	nuclear cap binding protein subunit 2	0.76	0.0334
MAPKAP1	mitogen-activated protein kinase associated protein 1	0.76	0.0491
RNF214	ring finger protein 214	0.76	0.0307
IRAK2	interleukin 1 receptor associated kinase 2	0.76	0.0379
CCM2	cerebral cavernous malformation 2	0.76	0.039
PDE5A	phosphodiesterase 5A, cGMP-specific	0.76	0.0296
GMDS	GDP-mannose 4,6-dehydratase	0.76	0.027
BCAR3	breast cancer anti-estrogen resistance 3	0.76	0.0393
SRRD	SRR1 domain containing	0.76	0.0289
LMNA	lamin A/C	0.76	0.035
XRN1	5-3 exoribonuclease 1	0.76	0.028
FAM155A	family with sequence similarity 155, member A	0.76	0.034
MAN1A2	mannosidase, alpha, class 1A, member 2	0.76	0.0373
GDE1	glycerophosphodiester phosphodiesterase 1	0.76	0.0419
VAMP1	vesicle associated membrane protein 1	0.76	0.0341
SNRNP35	small nuclear ribonucleoprotein, U11/U12 35kDa subunit	0.76	0.0487
PATZ1	POZ (BTB) and AT hook containing zinc finger 1	0.76	0.0488
DDX39B	DEAD (Asp-Glu-Ala-Asp) box polypeptide 39B	0.76	0.0375
RECQL5	RecQ helicase-like 5	0.76	0.0414
TINF2	TERF1 (TRF1)-interacting nuclear factor 2	0.76	0.0329
RRP8	ribosomal RNA processing 8, methyltransferase, homolog (yeast)	0.76	0.037
MFF	mitochondrial fission factor	0.76	0.0417
PRKG1	Memczak2013 ALT_ACCEPTOR, ALT_DONOR, coding, INTERNAL, intronic	0.76	0.033
FAM120B	family with sequence similarity 120B	0.76	0.0354
POLR2E	polymerase (RNA) II (DNA directed) polypeptide E, 25kDa	0.76	0.0426
SLC25A43	solute carrier family 25, member 43	0.76	0.0264
DHRS1	dehydrogenase/reductase (SDR family) member 1	0.76	0.0378
CCNG1	cyclin G1	0.76	0.0432
SH3RF1	SH3 domain containing ring finger 1	0.76	0.0435
RNF145	ring finger protein 145	0.76	0.0416
ILF3	interleukin enhancer binding factor 3	0.76	0.0348
SHOC2	SHOC2 leucine-rich repeat scaffold protein	0.76	0.0378
UTP4	UTP4 small subunit (SSU) processome component	0.76	0.0389
CMIP	c-Maf inducing protein	0.76	0.0297
TMEM192	transmembrane protein 192	0.76	0.0391
BRAP	BRCA1 associated protein	0.76	0.0392
TUBGCP5	tubulin, gamma complex associated protein 5	0.76	0.0297

EPHA2	EPH receptor A2	0.76	0.0359
SPTAN1	spectrin, alpha, non-erythrocytic 1	0.76	0.0405
N6AMT1	N-6 adenine-specific DNA methyltransferase 1 (putative)	0.76	0.047
IL6	interleukin 6	0.76	0.0374
MEIS1	Meis homeobox 1	0.76	0.0409
MRPL36	mitochondrial ribosomal protein L36	0.76	0.0367
SLC11A2	solute carrier family 11 (proton-coupled divalent metal ion transporter), member	0.77	0.0361
COMMD8	COMM domain containing 8	0.77	0.049
GDF5	growth differentiation factor 5	0.77	0.0498
PDZD11	PDZ domain containing 11	0.77	0.0429
PLPP3	phospholipid phosphatase 3	0.77	0.0474
MON2	MON2 homolog, regulator of endosome-to-Golgi trafficking	0.77	0.044
EDA2R	ectodysplasin A2 receptor	0.77	0.0363
SHB	Src homology 2 domain containing adaptor protein B	0.77	0.0361
RSL1D1	ribosomal L1 domain containing 1	0.77	0.0335
MLXIP	MLX interacting protein	0.77	0.0372
CDC42EP3	CDC42 effector protein (Rho GTPase binding) 3	0.77	0.0324
PXYLP1	2-phosphoxylose phosphatase 1	0.77	0.0353
ATP6V1E2	ATPase, H+ transporting, lysosomal 31kDa, V1 subunit E2	0.77	0.0495
CDC42	cell division cycle 42	0.77	0.0432
LMCD1	LIM and cysteine-rich domains 1	0.77	0.0348
MLF2	myeloid leukemia factor 2	0.77	0.0405
PSME3	proteasome activator subunit 3	0.77	0.0321
RPS4X	ribosomal protein S4, X-linked	0.77	0.0423
POLR3A	polymerase (RNA) III (DNA directed) polypeptide A, 155kDa	0.77	0.0493
NOL11	nucleolar protein 11	0.77	0.0359
PIGG	phosphatidylinositol glycan anchor biosynthesis class G	0.77	0.0338
GID8	GID complex subunit 8	0.77	0.0404
C1D	C1D nuclear receptor corepressor	0.77	0.0431
S100A6	S100 calcium binding protein A6	0.77	0.0491
HP1BP3	heterochromatin protein 1, binding protein 3	0.77	0.0459
NEO1	neogenin 1	0.77	0.0482
ZNF322	zinc finger protein 322	0.77	0.0394
IGF2BP2	insulin-like growth factor 2 mRNA binding protein 2	0.77	0.0429
CDC27	cell division cycle 27	0.77	0.0406
IFFO2	intermediate filament family orphan 2	0.77	0.0404
XIAP	X-linked inhibitor of apoptosis, E3 ubiquitin protein ligase	0.77	0.0454
SUGP2	SURP and G-patch domain containing 2	0.77	0.0411
FAM3C	family with sequence similarity 3, member C	0.77	0.0361
UBE2K	ubiquitin conjugating enzyme E2K	0.77	0.0481
SPAG9	sperm associated antigen 9	0.77	0.0396
UAP1	UDP-N-acetylglucosamine pyrophosphorylase 1	0.77	0.0484
HTRA1	HtrA serine peptidase 1	0.77	0.0393
RAP1GAP2	RAP1 GTPase activating protein 2	0.77	0.0479
ANKFY1	ankyrin repeat and FYVE domain containing 1	0.77	0.0407
FER	fer (fps/fes related) tyrosine kinase	0.77	0.0402
GDI2	GDP dissociation inhibitor 2	0.77	0.0452
BTN2A1	butyrophilin, subfamily 2, member A1	0.77	0.0351
NUAK2	NUAK family, SNF1-like kinase, 2	0.77	0.0489
DAW1	dynein assembly factor with WDR repeat domains 1	0.77	0.0488
SUCO	SUN domain containing ossification factor	0.77	0.0499
RPL10	ribosomal protein L10	0.77	0.0441
ZC3H14	zinc finger CCCH-type containing 14	0.77	0.0446
ZNF398	zinc finger protein 398	0.77	0.0451
FMNL3	formin like 3	0.77	0.0339
CITED2	Cbp/p300-interacting transactivator, with Glu/Asp rich carboxy-terminal domain,	0.77	0.0459
STX8	syntaxin 8	0.78	0.048
PHLDB2	pleckstrin homology-like domain, family B, member 2	0.78	0.0469
IYD	iodotyrosine deiodinase	0.78	0.0438

KCMF1	potassium channel modulatory factor 1	0.78	0.046
FAM114A2	family with sequence similarity 114, member A2	0.78	0.0468
NOL9	nucleolar protein 9	0.78	0.0467
PIGB	phosphatidylinositol glycan anchor biosynthesis class B	0.78	0.0386
CPA4	carboxypeptidase A4	0.78	0.0459
TTLL5	tubulin tyrosine ligase-like family member 5	0.78	0.0433
PRKCA	protein kinase C, alpha	0.78	0.0446
ZNF608	zinc finger protein 608	0.78	0.0408
ZDHHC23	zinc finger, DHHC-type containing 23	0.78	0.0442
APOBEC3C	apolipoprotein B mRNA editing enzyme, catalytic polypeptide-like 3C	0.78	0.0457
ARRDC4	arrestin domain containing 4	0.78	0.0487
TMEM265	transmembrane protein 265	0.78	0.0406
CAPZB	capping protein (actin filament) muscle Z-line, beta	0.78	0.0493
STAU1	staufen double-stranded RNA binding protein 1	0.78	0.0434
SEMA3C	sema domain, immunoglobulin domain (Ig), short basic domain, secreted,	0.78	0.0471
VEZF1	vascular endothelial zinc finger 1	0.78	0.0474
BEX4	brain expressed X-linked 4	0.78	0.0432
LCAT	lecithin-cholesterol acyltransferase	0.78	0.0445
SOCS4	suppressor of cytokine signaling 4	0.78	0.0489
TMBIM4	transmembrane BAX inhibitor motif containing 4	0.78	0.0461
NDST2	N-deacetylase/N-sulfotransferase (heparan glucosaminyl) 2	0.78	0.0467
PCCB	propionyl-CoA carboxylase beta subunit	0.79	0.0448
VCL	vinculin	0.79	0.0464
